# Methods used for successful follow-up in a large scale national cohort study in Thailand

**DOI:** 10.1186/1756-0500-4-166

**Published:** 2011-05-27

**Authors:** Sam-ang Seubsman, Matthew Kelly, Adrian Sleigh, Janya Peungson, Jaruwan Chokkanapitak, Duangkae Vilainerun

**Affiliations:** 1 Thai Health Promotion Centre, Sukhothai Thammathirat Open University, Nonthaburi, Thailand; 2 National Centre for Epidemiology and Population Health, the Australian National University, Canberra, Australia

## Abstract

**Background:**

Ensuring successful follow-up is essential when conducting a prospective cohort study. Most existing literature reviewing methods to ensure a high response rate is based on experience in developed nations.

**Findings:**

We report our 4-year follow-up success for a national cohort study examining the health transition underway in Thailand. We began the cohort study in 2005 with a baseline postal questionnaire sent to all 200,000 Thais enrolled as distance learning students at Sukhothai Thammathirat Open University and residing all over Thailand; 87,134 or 44% of the students responded. Subsequently we used University and national media to inform cohort members of study progress. Also, we prepared a health book with study results and health advice which was distributed to all cohort members. After 4 years we repeated the survey and achieved a 71% response rate. In this paper we report the methods used to achieve this response

The initial follow-up mail-out generated a response rate of about 48% reflecting the extensive preparatory work between baseline and follow-up. After 4 rounds of telephone contact (more than 100,000 phone calls) and 4 related mail-out rounds progressively over 16 months an overall response rate was achieved of just over 71% (n = 60,774). The total cost was US$4.06/respondent - 19% for printing, 21% for postage, 14% for tape measures (included in mail-out), 18% for data processing 22% for prizes and 6% for telephone.

**Conclusions:**

Many of the methods reported as effective for mail questionnaire and cohort response rates held true for Thailand. These included being associated with a university, incentivating cooperation, follow-up contact, providing a second copy of questionnaire where necessary, and assurance of confidentiality. Telephone contact with the cohort and the small prizes given to responders were particularly important in the Thai context as was Thai leadership of the research team.

## Background

When conducting prospective cohort studies one of the most important factors to consider is the ability to retain cohort members. Cohort retention is a particular challenge where the study is being conducted over a long period of time, when cohort members are part of the general population rather than a group defined by a certain characteristic such as belonging to a specific profession, workplace or town, and where all contact with the cohort is made by mail. This is the situation that the Thai Cohort Study faced when its first follow-up was attempted at the four-year point in 2008-9 and strategies adopted are described here.

There is already a large and mature literature dealing with mail-surveys. A meta-analysis of factors increasing mail-survey response rates noted that the best outcomes were associated with connection with a university, pre-notification by letter and telephone and supplying a stamped return envelope[1]. There is also a growing literature on cohort studies, particularly on factors which help contribute to cohort retention,[2-4] and a recent Cochrane Review on increasing responses to postal and electronic questionnaires[5]. Key retention factors include: frequent contact with the cohort members by mail, telephone, or in person; employment of enthusiastic committed staff who allow cohort members to feel connected to the research project; frequent constructive feedback to members on the progress of the research allowing them to bond to the project; providing incentives to participation including for example giving information on personal health status to members; and keeping a database recording all contact history and information on each member. For mail based cohort studies the use of a cover letter setting out confidentiality conditions, providing contact details if more information is needed and generally welcoming participation has been found to be helpful. Also the use of multiple waves of mail-outs combined with pre and post mail-out telephone calls have been effective in minimizing non-responders.

Most previous reports on successful cohort maintenance have been conducted in the USA or other western developed country settings[1-5]. There is little written about application to non-western developing countries. To address this gap, we report on methods employed for successful follow-up in a large mail based national cohort study in Thailand, a middle-income developing country in Asia. Our experiences may be useful for others attempting large scale cohort studies in similar settings.

## Methods and results

### Designing and generating the Thai Cohort

The Thai Health-Risk Transition Project began in 2004 with the aim of studying changes in the health status of the Thai population associated with rapid modernization and industrialization. Part of this study project has involved assembling a cohort of Thais who would be representative of the general population and whose health status could be followed through time along with their risk behaviour and socio-demographic and economic profiles. Our target population was persons studying by correspondence via Sukhothai Thammathirat Open University (STOU). This group was chosen because STOU students live throughout the country and display considerable variation in lifestyle, family structure, socio-economic status, domestic and occupational environment and personal behaviour. For almost all these factors STOU students are similar to the general Thai population[6]. To the best of our knowledge this type of nationwide representative cohort study has not been attempted before in Thailand with previous cohort studies on health risks being limited to specific population groups such as specific occupational groups,[7] sex workers,[8] drug users, [9] or prisoners[10].

In 2005 a questionnaire (Additional files 1, 2 and 3) was mailed to all of the approximately 200,000 students enrolled at STOU. We received back a total of 87,134 (44%) completed questionnaires which were used to gather information on various subjects associated with health, including demography, social networking, work, health services, disease and injury, environment, food and physical activity, smoking, alcohol and transport. Various methods were used to achieve this initial successful response rate. These included making clear our association with STOU by sending out our questionnaire together with other STOU materials as well as promoting ourselves on the STOU website and other University information outlets.

**Figure 1:**
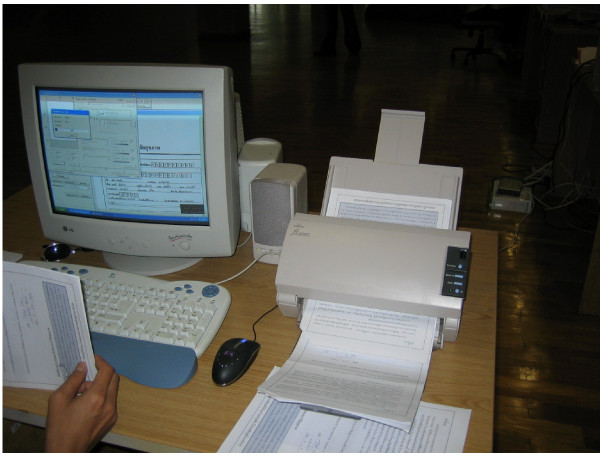
Scanning questionnaires and onscreen editing

When people responded and returned the questionnaire we scanned the data and created a digital data file and linked image file for each completed questionnaire. The scanning was completed using intelligent character recognition and editing software developed in Thailand called Scandevet (Figure 1)[11]. The personal identifying information for each individual record was connected to the digitized response data by an encrypting key with the code available only to the lead investigators in Thailand and the key stored in a locked safe. As well we created an additional SQL database containing the name, sex, birth date, address, telephone numbers, email address, student ID number, Citizen ID number, and Thai Cohort Study identifying number. This name-address database was constructed to enable subsequent changes of name, address or phone numbers as person-time accumulated. Periodically the name-address database file was updated and each individual record contained an update flag variable indicating if name or address had been changed. We conducted a 4-year follow-up study of this cohort in 2008/2009 and we summarize here the procedures used to maintain this contact and ensure a successful follow-up.

## Ethical Issues

Ethics approval was obtained from Sukhothai Thammathirat Open University Research and Development Institute (protocol 0522/10) and the Australian National University Human Research Ethics Committee (protocol 2004344). Informed written consent was obtained from all participants. All participants were free to withdraw from the study at any point and this was made clear in all correspondence. All personal identifying information was kept separately from the data set and was linked by an electronic code key ("dongle") only available to the Thai study director. The identifying data will never be available to data analysts and will only be used to make further contact in the future unless the cohort member has voluntarily withdrawn. All hard copy responses were destroyed by boiling the paper questionnaires after the data were digitized, edited and backed up.

### Maintaining the cohort between baseline and follow-up

#### Media

In the initial period after conducting the first questionnaire in 2005 we used media outlets to inform cohort members of ongoing project activities and research results. The first of these was STOU's quarterly newsletter sent out to all current students. We periodically contributed columns to this newsletter to keep students aware and connected to the project. The second media outlet used was the education supplement of a nationally distributed broad sheet newspaper (entitled *Khom Chad Leuk*). STOU has a fortnightly column in this paper to which our project contributed on a regular basis through 2006. We assumed that a significant proportion of our cohort would read this column as it is aimed at STOU students. Topics addressed included: food safety, maintaining a healthy heart, obesity, birthing and midwifery, the health-risk transition, sexual health among Thai youth and environmental influences on health. The third form of media used was a webpage within the university's website where we published periodic reports on project activities.

#### Linking up with the University's database

In June 2007 we cross checked our cohort database with STOU student records. Around 15% of our cohort members had registered a change of address or other contact details in the period between February 2005 when we began sending out our first questionnaire and June 2007. We then updated our contact details database to reflect these changes. This process was limited by the fact that it was not possible to trace address changes amongst those who were no longer enrolled as students and by May 2008 this affected 40% of the cohort.

#### Health Maintenance Handbook

**Figure 2:**
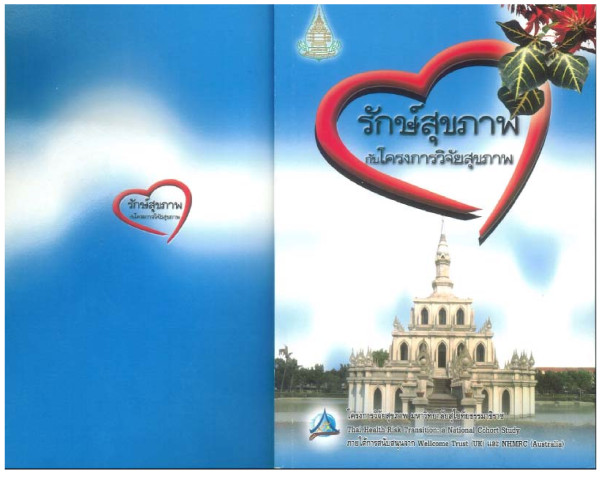
Cover of Health Maintenance Handbook distributed to cohort members

In November 2007 Thai investigators wrote and published a small book which reported the main findings of the 2005 questionnaire as well as giving advice on health maintenance including first aid, healthy diet and exercise regimes. This book was then mailed out to all cohort members. We aimed to give the members an understanding of the process involved in analyzing the data they had provided in order to give a sense of engagement with the project. As well as practical advice on health maintenance we described how the project could benefit the overall health of the Thai population (Figure 2).

We also used this mail out to update our address database. We inserted 2 pre-stamped change of address forms into the book so cohort members could report their current address easily. By April 2008, 5 months after the mail out of the health book we had received 2,256 forms informing us of changes in address, name or telephone number. For those members whose health books were returned as undeliverable we checked with the STOU student database to ascertain whether address had been changed.

#### Short trial questionnaire

In 2007, at the 2-year point of cohort follow-up a 10% random sample of the cohort was mailed a 2 page (single sheet) questionnaire (Additional files 3, 4 and 5), together with the Health Maintenance Handbook discussed above. The short questionnaire sought information on BMI, injury, mental health and occurrence of 25 common diseases. We also asked about email/internet access, change of email address and willingness to respond to the cohort follow up by e-contact. Overall 47% of those sent the short questionnaire mailed back a response; thus this response rate achieved in 2007 resembled that obtained at baseline when the cohort was generated in 2005 (44%). This 2007 response rate gave us some idea of what response rate we might expect for the 2009 follow-up if no additional strategies were deployed. In fact we could expect the 2009 follow-up to be less than the 2007 because of the additional time elapsed.

#### Use of the internet

In 2007 we also set up our project website at http://www.stoucohort.com. This website contains an overview of the project as well as up to date details of member activities. We also set up a system allowing cohort members to log in to the site and change their address and other personal details. We also collected information on use of the internet and email. At baseline (2005) 22% of cohort members had an email address. Two years later on the short questionnaire (2007) 38% had email address and 70% responded positively when asked if they would like electronic contact and follow-up. By April 2008, 5 months after mailing the Health Maintenance Handbook, 115 members had changed their personal details via the website.

#### Linking with death records

We established links with the Ministry of Interior and the Ministry of Public Health (MOPH) as both agencies keep death registers. We sent the Citizen ID numbers of all cohort members who had provided them (99% of the cohort) to the Ministries and they sent back a report of those who were deceased from this list. By August 2008 when the mail-out of the follow-up questionnaire was being prepared we had identified 251 (though we were still missing some data) of our cohort members as being deceased. We modified our database to ensure that deceased cohort members are not mailed follow-up material.

### The 2008-9 follow-up questionnaire

**Figure 3:**
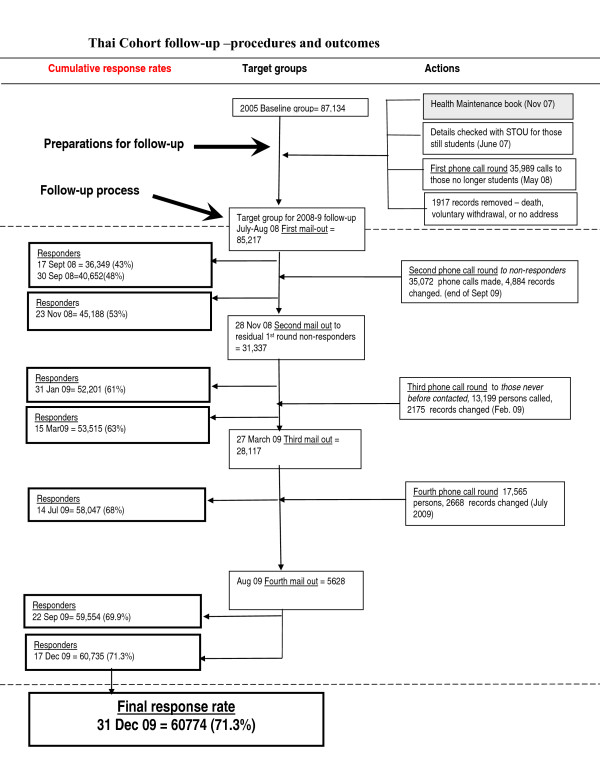
**Recruitment procedures and outcomes of 2008-9 cohort follow-up**.

The mail-out for this first complete cohort follow-up (Additional files 3, 6 and 7) involved 4 postage rounds with efforts made to ensure addresses were correct before each mail-out and to make phone contact with non-responders. This process is outlined below and is also graphically represented in Figure 3 with the left hand column representing our cumulative response rate over time, the central column our target populations for each successive mail-out round and the right hand column the actions taken to encourage further response. The Thai staff required for executing the follow-up study prepared mail-outs, made telephone contact and processed and scanned the bar-coded incoming questionnaires. This involved a core group of 8 full time staff with an additional 7 to 8 staff available for peak periods.

### 1^st ^round of mail-out

#### Preparing for the first round mail-out: 1^st ^round phone call

Our initial step in preparing for the first mail-out round was to call as many cohort members as was feasible before the mail-out to confirm contact details and encourage participation. We focused on those cohort members most likely to have changed address since their most recent STOU database update - those 36,000 people who were no longer currently enrolled as students. To conduct this task several supervised teams were formed and telephonists were trained to be polite, diplomatic, and accurate, following strict guidelines for both speaking and recording. All team members had vocational school or university education. Regional teams included 3 - 10 people located in Bangkok, the northeast (Khon Kaen) and the south (Surat Thani). This initial telephone round took place between May and June 2008 (Table 1).

**Table 1:** Results of phone calls made to cohort members

First phone call round - (those no longer registered as students)		No. of cases	
Unable to contact^1^		19,976	

Able to contact			

- Details correct		11011	

- Details changed		5002	

**Total numbers of contacts attempted**		**35,989**	

			

**Second phone call round - (non-responders)**			

Unable to contact^1^		17344	

Able to contact		17728	

**Had received q'nnaire**	**Had returned q'nnaire**	**Action taken**	**No. of cases**

Yes	Yes	Thank member	3067 (17.3%)

Yes	No	Request cooperation	8634 (48.7%)

No	-	Check address for next mail-out round	4361 (24.6%)

Unsure (but address correct)	-	Add to next mail-out list	1666 (9.4%)

			

**Third phone call round (to those never before contacted)**			

Unable to contact^1^		9154	

Able to contact			

- Details correct		1870	

- Details changed		2175	

**Total number of contacts attempted**		**13199**	

			

**Fourth phone call round (to remaining non-responders)**			

Unable to contact^1^		10207	

Able to contact			

Able to contact			

- Details correct		5040	

- Details changed		2602	

**Total number of contacts attempted**		**17,849**	

^1^Persons unable to be contacted includes those whose numbers were busy, had no answer, who were no longer at that number and those who were deceased.

After these phone calls the cohort database individual records were updated as follows: deceased members (289), duplicates (191), inadequate address (1598), and voluntary withdrawal (20). From the original cohort of 87,134 we were then left with 85,217 individuals to whom we would send the follow-up questionnaire.

#### 1^st ^round mail-out process

**Figure 4:**
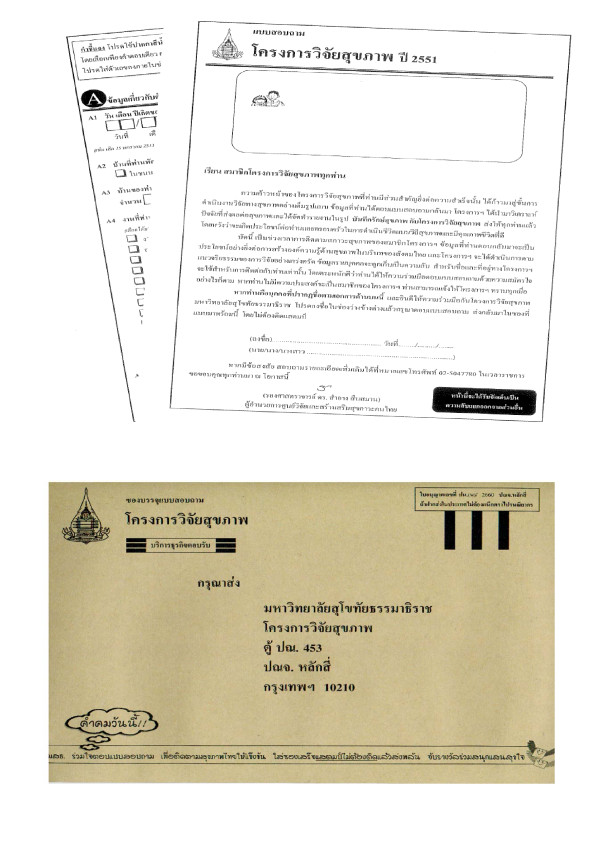
Cover sheet of 2008-9 follow-up questionnaire

Between the 25^th ^of July and 30^th ^of August 2008, 85,217 12-page follow-up questionnaires were mailed out. As well as the questionnaire itself mail out materials included: 1) a stamped pre-addressed return envelope (Figure 4), 2) a message from the President of the University assuring members of STOU's support for the project and encouraging people to respond, 3) a message from the project team (including a team photo) reminding members of some of the background to the project, assuring them of the confidentiality of their responses and giving them contact details if they needed more information, 4) a tape measure to assist members with answering questions on waist and hip measurement, and 5) a coloured flyer advertising prizes including t-shirts, bags and scholarship funds, winners of which would be drawn from among respondents. All questionnaires were individually pre-barcoded and by scanning barcodes on incoming completed questionnaires we had a constantly updated list of follow-up participants (Figure 1).

#### 2^nd ^phone call round

By the end of September 2008 we had received 40,652 completed questionnaires from the first mail-out, a response rate of 48% (Figure 4). We then began a second round of phone calls to confirm details of cohort members who had not responded and who we did not or could not contact on the 1^st ^round of phone calls. In this phone call round we attempted to match telephonists familiar with particular regional dialects with cohort members in those areas. Contacted cohort members were asked if they had received the questionnaire and if so encouraged participation; if not, we checked the accuracy of the address we had for that person and included them with their new address in the next mail-out round. The results of this second phone call round are shown in Table 1.

### 2^nd ^round of mail-out

By November 2008 we had already achieved an overall 53% response rate with 45,188 completed questionnaires from the 1^st ^mail-out round (Figure 4). We had finished updating our records as a result of the phone calls described above, prepared a second mail-out list and had another round of questionnaires printed. This list of 31,337 people was made up of cohort members whose questionnaires were not returned or which were returned by the post office as undeliverable and whose address had been updated by phone call.

In this 2nd mail-out round questionnaires were mailed with different coloured send out and return envelopes from the 1st round to allow us to differentiate between them. It was mailed out between the 26^th ^of November and the 15^th ^December 2008. By the end of January 2009 we had received 52,201 completed questionnaires, an overall response rate of 61% (Figure 4).

#### 3^rd^phone call round

In February 2009 we began another round of phone calls attempting contact with only those non-respondents who had never been contacted by TCS before, including those who had a busy phone signal or who didn't answer on previous contact attempts. This left us with a total of 13,199 persons to contact. The phone call round ended on 15 March 2009 (Table 1).

By the end of March 2009, we had achieved a 63% (53,639) response rate from the 2 rounds of mail-out (Figure 4). After removing those whose questionnaires had been returned as unforwardable (and who we couldn't get a new address for) and those who we knew to be deceased we then prepared for a 3^rd ^mail-out round.

### 3^rd ^round of mail out

By March 2009 a list of potentially contactable non-respondents included 28,117 persons and these were the target of a third mail-out round in March and April 2009. These 3^rd ^round questionnaires were sent and returned in envelopes of a 3^rd ^distinct colour to differentiate from the previous 2 rounds. By July 2009 we had received a total of 58,047 responses, a 68% response (Figure 4).

#### 4^th ^phone call round

As of July 2009 the total number of potentially contactable non-responders who had previously reported at least one phone number was 17,849. We attempted a final phone call (Table 1) and mail-out round.

### Fourth round mail-out

A 4th round mail-out began in August 2009. Questionnaires were only sent in this round to those successfully contacted by phone in the last phone round who reported they had not yet received a questionnaire or had misplaced it. We were able to contact 7,642 persons and needed to send 5,628 4^th ^round questionnaires to those who did not have a copy in their possession.

**Figure 5:**
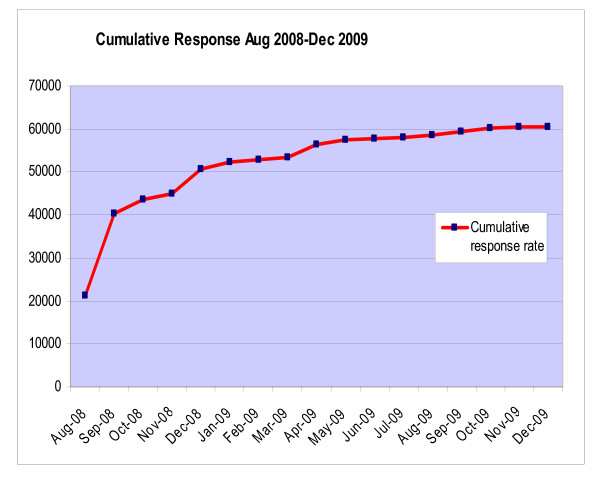
**Cumulative response August 2008-December 2009**.

When the cohort follow-up was finally closed down in December 2009 the total number of completed questionnaires received was 60,774, a response rate of 71% (Figure 4). The overall cost involved in the process described above was around $4 US dollars per returned questionnaire. The largest part of this cost was made up of the gifts/prizes given (21.9%) and the postage (20.8%) (Table 2). It is not possible to know which of the various cost elements was the most beneficial but it is interesting to note that the telephone costs (6%) were relatively much smaller than the other major items. For an overview of the final results we have prepared a cumulative response graph (Figure 5) showing the change over time from beginning to end of the follow-up process.

**Table 2:** Costs of conducting cohort follow-up

Cost category	Cost Thai baht*	% of total cost
Printing	1,657,000	19.2

Postage	1,793,000	20.8

Tape measures (included in mail-out package	1,220,000	14.1

Gifts/prizes	1,894,000	21.9

Data input and barcode scanning	1,546,000	17.9

Phone contact with cohort	529,000	6.1

**Total**	**8,639,000**	**100**

* US$1 = 35 Thai baht

## Discussion

### Follow-up success

As of December 2009 we had achieved an overall response rate of just over 71% for our follow-up questionnaire. This response exceeded the 44% achieved in 2005 when the cohort was generated at baseline from 200,000 Open University students; it was also substantially better than the 47% response achieved with the short trial questionnaire follow-up of a 10% sample of the cohort in 2007. It should be noted that response rates less than 20% to mail out surveys are not uncommon and indeed 18% was the comparable figure noted for the baseline survey for the "45 and Up" cohort study in Australia; this study used Health Insurance Commission contact details, thought to be the best location database for community recruitment [12]. Another famous Australian cohort study is the Australian Longitudinal Study on Women's Health. This study also used the Health Insurance Commission database to recruit their sample of various ages and on 4-year follow-up achieved a 64.3% response rate for women of comparable age to most of those in our Thai cohort [13]. A similar 4-year follow-up response rate (62%) was reported by one of the largest cohort studies ever attempted, the UK's Million Women Study [14]

It should also be noted that cohort studies are very diverse in their design and it is not necessarily informative to rate them according to reported (or unreported) longitudinal attrition. Here we reported a 4-year 2009 outcome of 71% follow-up for the Thai Cohort Study. This was a good follow-up result especially notable given that the cohort respondents were free-living members of the Thai population residing all over the nation. Such a cohort study is especially liable to encounter problems with attrition compared to studies based on a single profession (eg nurses or doctors), one town (eg Framingham, Massachusetts) or one industry (eg Thailand's EGAT study based around an electricity generating plant). Our good 4-year result required planning and sustained input by a large group of experienced Thai and Australian investigators over a long period. In the next section we will review the key features and strategies that led to success.

Despite achieving a 71% response rate a problem remains of non-responder bias. Preliminary analysis indicates a small tendency for non-response to be higher for younger age groups, males and lower income groups - otherwise non-responders to our 2009 survey were remarkably similar to responders on a wide variety of socio-demographic attributes. Other studies have been less fortunate and found both that non-responders can differ markedly from responders and also that participants who are lost to the study may differ more from respondents than those who declined to join the study from the beginning[1,15]. Late responders who responded in the third and fourth mail-out rounds may be shown to be more similar to non-responders than those who responded straight away[16]. To some extent non-response bias can be estimated during data analysis. Of course the best solution to non-response bias is to minimise non-response and that is why we consider this report of our experiences is relevant to those designing and operating cohort studies.

### Features of the Thai Cohort Study

Key features of the Thai Cohort Study include the following: all strategic decisions were led from Thailand and incorporated the Thai cultural and logistical context; several investigators had worked on cohort studies before; the data management involved Thai experts at all times and was conducted in the Thai language; both Thai and Australian teams had bilingual members; adequate funding enabled the baseline and follow-up; the study baseline data led to over 30 scientific papers thus boosting team morale; formal support by STOU has been made apparent to respondents.

### Strategies for follow-up

Important strategies adopted by the study team included the following: introducing individual barcodes for the follow-up questionnaire facilitating real-time monitoring of responses; updating contact details for cohort members through repeated and focused telephone campaigns; enhancing trust by using female telephonists who formally introduce themselves and give a contact landline phone number for the project office at STOU; not annoying cohort members by calling an excessive number of times or at inappropriate times of the day; adapting our cohort postal system to the Thai postal service and STOU postal systems lowering costs because of bulk return delivery to STOU and bulk reply paid fees, minimizing postal losses and building trust among cohort members; maintaining cohort members' interest in the research by reporting frequently; giving incentives for participation (small gifts or small educational grants); sustaining a high professional standard for the quality and content of all communication and questionnaires; appealing to national pride of cohort members by pointing to the national public health benefit of the research.

An enormous effort went into constructing questionnaires, revising through 20+ drafts, pre-testing extensively, and back translating to ensure language validity. As well, the physical appearance of printed materials distributed to cohort members was paid great attention; Thai team members produced a distinctive logo and combined this with humorous cartoons and images which were displayed in all correspondence. This process was also personalized by adding a personal letter and photograph of the chief Thai investigator. At all times cohort members were reminded of the strict confidentiality in which their responses would be kept to encourage response.

## Conclusions

The methods which appear to have been substantially successful in our follow-up questionnaire broadly follow those which have proven successful in other published studies on increasing response rates, especially the focused use of telephone calls for both pre notification and confirmation of contact details and for follow up of non-responders [1-4].

Based on our intense experience of devising and managing this study now entering its 6^th ^year we feel confident that the keys to success rest on the etiquette we have followed. Especially important was our telephone behaviour when updating addresses, our guarantee of confidentiality, our high physical and intellectual standard for all correspondence and questionnaires, our multiple feedback systems and our judicious use of incentives and small rewards. It is possible that other strategies not deployed could have boosted response rates further but we were unable to identify additional affordable strategies beyond those described here. It should be noted here that it is still too early to attempt e-questionnaires on our cohort as less than 10% have ready internet access. Furthermore experience with e-cohorts internationally is still limited and while costs may be lowered response rates have been reported to be below 5%[17].

We display in Figures 3 and 4 the cumulative effect of the processes we followed between the baseline and follow-up waves of our study and during the process of rolling out the follow-up questionnaire. Although our study does not enable us to address in an experimental fashion what would have been the effect of different follow-up methods these figures indicate the effect of the methods we did use. Our experience confirms that some of the key factors related to follow-up success as reviewed at the beginning of this paper were also operating for us [1-5]. Thus we had a university connection, pre-notification letter, frequent contact and feedback, positive staff attitudes, various incentives, informative cover letters, up-to-date contact details and repeated waves of mail-out plus supportive phone calls

In this paper we have summarised our experience and analysed the follow-up process using the available data; we were funded to study the health-risk transition and we were not funded to conduct a methodological study. However, the information we present here would have been useful for us at the start and should assist others planning similar large cohort studies.

## Competing interests

The Authors declare that they have no competing interests.

## Authors' contribution statement

SS, AS, JC, MK, DV and JP have all been fundamentally involved in the conception and design of the cohort study described here as well as the acquisition of data.

DV, JC, SS and JP have been responsible for generating the data.

MK, DV and AS have created the draft of the manuscript and all authors have been involved in revising the manuscript and given final approval for submission.

## Supplementary Material

Additional file 1 **Baseline 2005 Questionnaire (English)**. An English translation of the questionnaire sent to all 200,000 students enrolled at Sukhothai Thammathirat Open University in 2005 Click here for file

Additional file 2 **Baseline 2005 Questionnaire (Thai)**. The actual Thai language questionnaire sent to all 200,000 students enrolled at Sukhothai Thammathirat Open University in 2005 Click here for file

Additional file 3 **Thai fonts**. These fonts are required for all elements of the questionnaires which form additional file 1, 2, 3, 4, 5, 6 to display correctly. These files need to be saved to the font directory of your computer before opening the questionnaire files. Click here for file

Additional file 4 **2007 short follow-up questionnaire (English)**. An English translation of the short 2 year follow-up questionnaire sent to a random 10% sample of respondents to the 2005 baseline questionnaire Click here for file

Additional file 5 **2007 short follow-up questionnaire (Thai)**. The Thai language short 2 year follow-up questionnaire sent to a random 10% sample of respondents to the 2005 baseline questionnaire Click here for file

Additional file 6 **2009 4-year follow up questionnaire (English)**. An English translation of the 4-year follow-up questionnaire mailed out to all cohort members (n = 85,217) in 2009. Click here for file

Additional file 7 **2009 4-year follow-up questionnaire (Thai)**. The Thai language 4-year follow-up questionnaire mailed out to all cohort members (n = 85,217) in 2009. Click here for file

## References

[B1] Fox RJ Crask M Kim J Mail survey response rate: A meta-analysis of selected techniques for inducing response Public Opinion Quarterly 1988 52 467 491 10.1086/269125

[B2] Hunt JR White E Retaining and tracking cohort study members Epidemiologic Reviews 1998 20 1 57 70 9762509 10.1093/oxfordjournals.epirev.a017972

[B3] Marmor JK Oliveria SA Donahue RP Garrahie EJ White MJ Moore LL Ellison RC Factors encouraging cohort maintenance in a longitudinal study Journal of Clinical Epidemiology 1991 44 531 535 10.1016/0895-4356(91)90216-V 2037857

[B4] Given BA Keilman LJ Collins C Given CW Strategies to minimise attrition in longitudinal studies Nursing Research 1990 39 184 186 2342908

[B5] Edwards PJ Roberts I Clarke MJ DiGuiseppi C Wentz R Kwan I Cooper R Felix LM Pratap S Methods to increase response to postal and electronic questionnaires (Review) Cochrane Database of Systematic Reviews 2009 3 10.1002/14651858.MR000008.pub4 PMC8941848 19588449

[B6] Sleigh A Seubsman SA Bain C The Thai Cohort Study Team Cohort Profile: The Thai Cohort of 87 134 Open University Students International Journal of Epidemiology 2008 37 266 272 10.1093/ije/dym161 17911153

[B7] Sritara P Cheepudomwit S Chapman N Woodward M Kositchaiwat C Tunlayadechanont S Sura T Hengprasith B Tanphaichitr V Lochaya S Neal B Tanomsup S Yipintsoi T Twelve-year changes in vascular risk factors and their associations with mortality in a cohort of 3,499 Thais: the Electricity Generating Authority of Thailand study International Journal of Epidemiology 2003 32 461 468 10.1093/ije/dyg105 12777437

[B8] Limpakarnjanarat K Mastro TD Saisorn S Uthaivoravit W Kaewkungwal J Korattana S Young NL Morse SA Scmid DS Weniger BG Nieburg P HIV-1 and other sexually transmitted infections in a cohort of female sex workers in Chiang Rai, Thailand Sexually Transmitted Infections 1999 75 30 35 10.1136/sti.75.1.30 10448339 PMC1758174

[B9] Subbarao S Vanichensi S Hu DJ Kitayaporn D Choopanya K Raktham S Young NL Wasi C Sutthent R Luo CC Ramos A Mastro T Genetic characterization of incident HIV type 1 strains subtype E and B strains from a prospective cohort of injecting drug users in Bangkok, Thailand AIDS Research and Human Retroviruses 2000 16 8 699 707 10.1089/088922200308693 10826476

[B10] Thaisri H Lerwitworapong J Vongsheree s Sawanpanyalert P Chadbanchachai C Rojanawiwat A Kongpromsook W Paungtubtim W Sri-Ngam P Jaisue R HIV infection and risk factors among Bangkok prisoners, Thailand: a prospective cohort study BMC Infectious Diseases 2003 3 25 10.1186/1471-2334-3-25 14580265 PMC270083

[B11] Thinkhamrop B Mongkolchati A Seubsmarn S Vilainerun D Sleigh A Thai Cohort Team. Large Cohort Data: New Thai System for its National Cohort Study Epidemiology & Equity in Health: Methodological Challenges & Strategies for the 21th Century The XVIIth IEA World Congress of Epidemiology, 21-25 August 2005, Bangkok, Thailand21623595

[B12] 45 and Up Study Collaborators Cohort Profile: The 45 and Up Study International Journal of Epidemiology 2008 37 5 941 947 17881411 10.1093/ije/dym184 PMC2557061

[B13] Lee C Dobson AJ Brown WJ Bryson L Byles J Warner-Smith P Young AF Cohort Profile: The Australian Longitudinal Study on Women's Health International Journal of Epidemiology 2005 34 987 991 10.1093/ije/dyi098 15894591

[B14] Banks E Beral V Reeves G Balkwill A Barnes I Fracture incidence in relation to the pattern of use of hormone therapy in post-menopausal women Journal of the American Medical Association 2004 291 18 2212 2220 10.1001/jama.291.18.2212 15138243

[B15] Asch DA Jedrziewski MK Christakis NA Response rates to mail surveys published in medical journals Journal of Clinical Epidemiology 1997 50 10 1129 1136 10.1016/S0895-4356(97)00126-1 9368521

[B16] Armstrong JS Overton TS Estimating non-response bias in mail surveys Journal of Marketing Research 1977 14 396 402 10.2307/3150783

[B17] Turner C Bain C Schluter PJ Yorkston E Bogossian F McClure R Huntington A the Nurses and Midwives e-cohort Group Cohort profile: The Nurses and Midwives eCohort study - a novel electronic longitudinal study International Journal of Epidemiology 2009 38 53 60 18202083 10.1093/ije/dym294

