# Supplementary material for: Methods used for successful follow-up in a large scale national cohort study in Thailand
Source: BMC Res Notes. 2011 May 27;4:166. doi: 10.1186/1756-0500-4-166 (PMC3123220; doi:10.1186/1756-0500-4-166)

Questionnaire

The Thai Health-Risk Transition: A National Cohort Study

Student Identification Number N N N N N N N N N N

**Details about respondent**
Citizen Identification Number N N N N N N N N N N N N N<br>Name..Family name..<br>Address: No.........................Moo Ban..................Soi....................Road..................................<br>Tambol/Kwang............................District/Khet............................Province........................<br>Post code N N N N N<br>Home Tel. ....Office Tel............................Mobile…..<br>e-Mail………………………………………………………………

This page will be separated and treated as confidential

**Other contact person (If we cannot contact you)**
Name..Family name..<br>Address: No.........................Moo Ban..................Soi....................Road..................................<br>Tambol/Kwang............................District/Khet............................Province........................<br>Home Tel. ....Office Tel............................Mobile…......................................<br>.

Questionnaire Code N N N N N N

(Official use only)

****

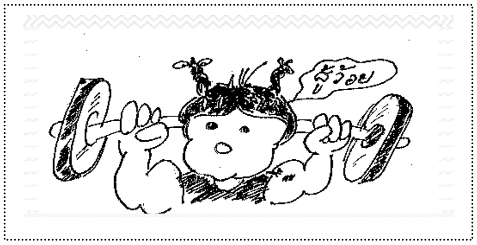

Information

The questionnaire of the Thai Health-Risk Transition Project aims to study the health transition of Thai people in order to understand factors affecting health which will render the healthy public policy recommendation in the future. This study considered the students of Sukhothai Thammathirat Open University as the representatives of the Thai population.

The success of this study depends on co-operation from STOU students in responding to the questionnaire. Participation to the study is voluntary with no influence on academic results at STOU.

All information will remain confidential and will only be used in the research work. None of the information will be disclosed unless permitted by the respondent.

Further information about the project can be obtained or if you have any queries please contact Dr. Sam-ang Seubsman, Thai Health-Risk Transition Office, Room 101 Tri Sorn Building, Sukhothai Thammathirat Open University, Tel/Fax 02 504 7780 or Email tcs@stou.ac.th or visit website: http://www.stou.ac.th/ANU/

If you agree to take part in the Thai Health-Risk Transition Project , please sign in the space below.

I agree to participate in the “Thai Health-Risk Transition : A National Cohort Study”

Name..
( )
date ..../............../...............

Special for Thai Health-Risk Transition Project member

Free!

Within 2 months of returning this questionnaire to STOU, you will be able to access **MyData Base** which will act as your own data base in the internet with free capacity of 20MB for one year. You can create your own website under 5 names and there are also many tools available. You can access by going to **http://www.tcsstou.net** and type in student ID as your Username. As for the Password please type in your birth date which consists of 8 digits (as answer in A1) eg. birth date of March 5, 2512 (B.E.), the Password is 05032512. Please change your Password after first access for confidentiality.

We still have special arrangement for you, please turn to the back cover for information

Please tick (✓)in the b in front of the selected choice to get to this image ☑ Please select only one answer except for those that indicate (please tick all that apply) after the questions. For questions that ask for numeric answers, please write the correspondent number in the box N one by one by using dark colour pen. (black or blue)

A YOU AND YOUR HOME

A1 In what year were you born? (Please use numbers only)

N N / N N / N N N N

Day month year (B.E.)

(Please complete in numeric form for 8 digits which will be used as a password for MyDataBase at http://www.tcsstou.net within 2 months after receiving the questionnaire)

A2 You are:- b Male b Female

A3 How many brothers do you have in total?

(*not include yourself*) N N persons

A4 How many sisters do you have in total?

(*not include yourself*) N N persons

A5 Where did you come in the order of births? N N

A.6 What is your highest level of education (do not include the your current STOU degree)?

- B junior high school or equivalent
- B high school or technical equivalent
- B post-high-school diploma/certificate
- B bachelor or higher university degree

A7 Do you think you have ethnic or cultural links to any of the following groups? *(tick all that apply)*

- B Chinese
- b Mon
- B Central Thai
- b North-eastern Thai
- B Northern Thai
- B Southern Thai
- B Other groups

A8 What is your current status?

- b Single → **go to A11**
- b Living with partner → **go to A11**
- b Married

A9 What is your current marital status? *(tick one only)*

- B Married – first and only marriage
- b Remarried – second or later marriage
- b Separated from someone you have been married to (but not divorced)
- b Divorced
- b Widowed

A10 What age did you first marry? N N year old

What is 'home'? For the next questions, if you:
- have just one home always answer about that home
- are 4 days/month or more at a ***permanent*** *home, answer for that home*
- are less than 4 days/month at a permanent home answer for the ***working*** *home*

Questionnaire Code N N N N N N
(Official used only)

zz

A11 Apart from your permanent family home do you stay at another residence when you are working?

B Yes

b No ⟶ **skip to A13**

A12 how often are you at the permanent family home?

N N days/month (average)

A13 In <u>the last 5 years</u>, have you moved your permanent family home?

B Yes b No ⟶ **go to A15**

A14 Which of the following best describes your last move :

B rural-to-rural

B rural-to-urban

B urban-to-urban

B urban-to-rural

A15 Who is the head of your home?
(choose one answer that fits best)

b I am

b My husband/wife

b My father

b My mother

b My father-in-law

b My mother-in-law

b Relatives/another male

b Relatives/another female

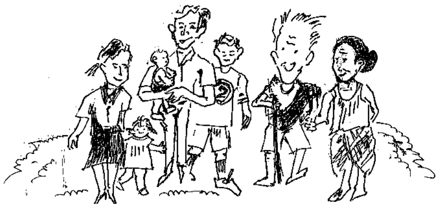

A16 How many people in total live in your home, including yourself? N N

A17 How many people <u>aged 15 years or under</u> live in your home? N N

If no ,pleasefill 0in the box N N *as follow* 0 0

A18 Do any of the following people usually live in your home? *(tick all that apply)*

B Spouse / partner

B Adult son/daughter (aged 16 years or more)

B Adult brother/sister (aged 16 years or more)

B Parent

B Grandparent

B Other relative/son-in-law/daughter-in-law

B Non-relative

A19 What best describes your home?
(choose <u>one</u> that fits best)

B Detached house

B Semi-detached house

B Shop-front or row-house

B Town house

B Dormitory, flat, apartment, condominium

B Other

A20 Is your home...

B Owned by you and/or your spouse?

B Hire purchased (mortgaged)?

B Not owned but rent free? (e.g. living with parents)

B Rented?

B Other?

A21 How much of a problem are the following within 1 km of your home? (Tick ✓ *that fits best in each item)*

	A big problem	A bit of a problem	Not a problem	Do not know
Air pollution				
Water pollution/waste water				
Noise				
Industrial chemicals				
Agrochemicals				
Pesticides/ herbicides				
Rubbish lying around				
Bad odours				
House break ins				
Poor public transport				
Lack of footpaths				
Lack of recreation areas				
Dogs that may bite				

Below we ask about your home now and as you recall from your childhood when you were **about 10–12 years of age**. This allows us to look at transitions and time effects.

A22 your home now and as you recall from your childhood

Where is your current permanent home located now? b Countryside b City/Town

Where was your permanent home when you were a child ? b Countryside b City/ Town

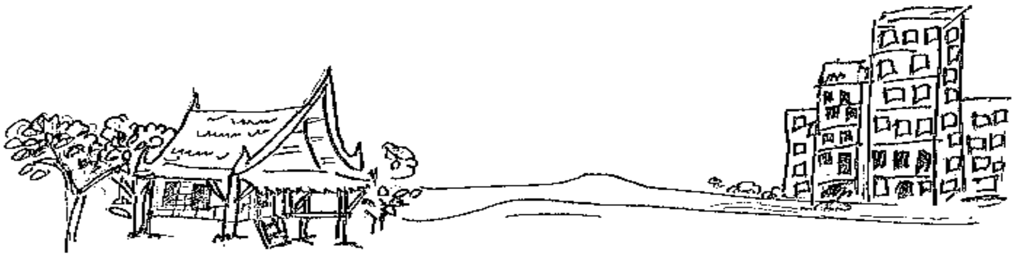

Question about your childhood

A23 Which of the following did your home have when you were **10-12 years-old (Grade 4-6)**?

(tick all that apply)

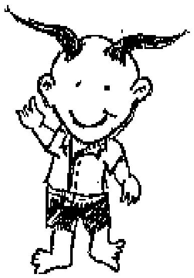

B	Electricity – local generator	B	Radio
B	Electricity – outside line	B	Computer
B	Microwave oven	B	Telephone
B	Refrigerator	B	Mobile phone
B	Electric fan	B	water heater
B	Air conditioning	B	Washing machine
B	Television	B	Mosquito bed-net
B	Video / tape/ CD player	B	Mosquito wire-net
		B	Do not have the above items

A24 What was the main source of your home drinking water when you were **10-12 years-old (Grade 4-6)**? *(whether filtered or not)*

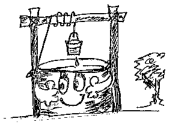

B	Piped supply	B	River, canal, stream, pond or lake
	Well or underground water	B	Bottled water
B	Rain water	B	Water from other source (e.g. commercial dispenser)

Questions about present time

A25 Which of the following does your home have **now**? *(tick all that apply)*

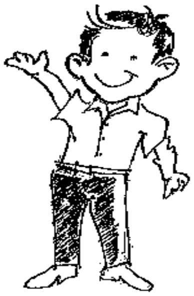

B	Electricity – local generator	B	Radio
B	Electricity – outside line	B	Computer
B	Microwave oven	B	Telephone
B	Refrigerator	B	Mobile phone
B	Electric fan	B	water heater
B	Air conditioning	B	Washing machine
B	Television	B	Mosquito bed-net
B	Video / tape/ CD player	B	Mosquito wire-net
		B	Do not have the above items

A26 What is the main source of your home **drinking water now**? *(whether filtered or not)*

B	Piped supply	B	River, canal, stream, pond or lake
B	Well or underground water	B	Bottled water
B	Rain water	B	Water from commercial dispenser

B INCOME AND WORK

B1 What is your average personal monthly income?

B less than 3,000 Baht   B 3,001-7,000 Baht   B 7,001-10,000 Baht
B 10,001-20,000 Baht   B 20,001-30,000 Baht   B over 30,000 Baht

B2 Do you work for income?

B Yes → **go to B4**   b No

B3 If not working for income at present are you best described as:- *(tick one option that fits best)*

B Homemaker ?
B Unpaid family worker (not homemaker)
B Wholly Retired? (do not work for income)
B Unable to work owing to permanent sickness/ disability ?
B Seeking work for first time?
B Unemployed ?
B Student ? (do not work)
B Other?

For those who do not work for income after answered B3 please go to C1

B4 Which category best fits your main job? *(Please tick one only)*

b Government/state enterprise/Institution employee
b Private employee
b Employer → **go to B 7**
b Self-employed (without employees, not agriculture) → **go to B 7**
b Own farm/ garden → **go to B 7**
b Other → **go to B 7**

B5 If employed as Government/state enterprise/Institution/Private employee for your main income-earning job, are you….

B Permanently employed
b On a fixed length contract (e.g. 1 year contract or more)
b Casually employed
b Other

B6 How many hours do you usually work each week in all paid jobs? N N total hours

B7 Do any paid hours involve shift work at night or weekends?

B Yes　　b No

B8 How secure do you feel about your job or career future in your current occupation?

b Not at all secure　　b Moderately secure
b Secure　　b Extremely secure

B9 How often do you extend your workday to continue after 6pm? (paid or unpaid)

b 5-7 days/week　　b 2-4 days/week
b 1-4 times/month　　b Less often
b Never

B10 In total, on average how long per day do you spend traveling from home to work (one-way)?

B Less than 30 minutes　　b 30 minutes – 1 hour
b 1-2 hours　　b 2-3 hours
b More than 3 hours　　b Do not have to travel

B11 In your main income-earning job, how would you describe yourself? *(you may tick more than one)*

B professional (eg accountant, doctor)　　b skilled worker (eg carpenter, dressmaker)
b senior manager　　b middle manager
b office assistant　　b manual worker
b other

B12 Your work situation. (Please tick ✓ to fit best for each question)

**Working situation**	**Often**	**Sometimes**	**Rarely**	**Never**
I have a good deal of say in decisions about work				
My working time can be flexible				
I have to do the same thing over and over again on a regular basis				
I have enough time to do everything				
I have to work very fast				

B13 During the <u>last 12 months</u>, how often have you experienced at work each of the following? *(Please tick ✓ to fit best for each question)*

**Environment**	Often	Sometimes	Rarely	Never	Don't know
Vibrations from hand tools or machinery					
Noise so loud that you had to raise your voice to talk to people					
High temperatures which make you uncomfortable					
Temperatures too low					
Vapours, fumes, dust, or dangerous substances (such as chemicals, infectious materials)					
Handling or touching dangerous products or substances					

C YOUR HEALTH, INJURIES AND HEALTH SERVICE USE

C1 What is your weight? N N N Kilograms

C2 What is your height without shoes? N N N Cm

C3 Which best describes your size or weight (recalled by relatives) <u>when you were born</u>?

B Small or under weight
b Normal
b Large or over weight
b Do not know → **skip to C5**

C4 My <u>birth-weight</u> was (if known): N N N N grams

C5 Recalled by relatives, when you were an infant were you breast fed ?

b Yes
b No → **skip to C7**
b Do not know → **skip to C7**

C6 <u>If Yes</u>, how long were you breast fed? N N months

C7 Which statement best describes your current eyesight?

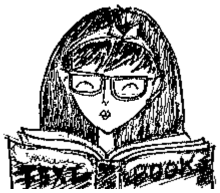

b I do not need glasses
b I need glasses/contact lenses since childhood (<13 years old)
b I need glasses/contact lenses since a teenager (13-19 years old)
b I need glasses/contact lenses since adulthood (20 years old or more)

C8 Do you currently have any sight problems not correctable by glasses/contact lenses? (eg cataract etc.)

b Yes b No

C9 Which statement best describes your current hearing (without a hearing aid)?

B Good
b Some trouble, since childhood (<13 years old)
b Some trouble, since teenage or adult (13 years or more)
b Deaf since childhood (<13 yrs old)
b Deaf since teenage or adult (13 years or more)

C10 Adults can have up to 32 natural teeth. How many of your own teeth do you have?

b None b 1-5
b 6-19 b 20 or more

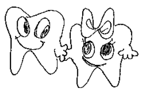

C11 Do your teeth or dentures currently cause you…*(please tick all that apply)*

b discomfort speaking?
b discomfort chewing?
b Pain
b discomfort swallowing?
b Loss of confidence when engaging in social interaction
b None of these

C12 In the past 4 weeks, about how often… (Please tick ✓ to fit best for each question)

	**All of the time**	**Most of the time**	**Some of the time**	**A little of the time**	**None of the time**
Did you feel nervous?					
Did you feel restless or fidgety?					
Did you feel that everything was an effort?					

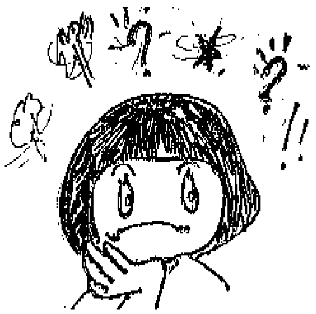

C13 The next questions ask about some health conditions whether you ever been told by a doctor that you have this condition. *(please tick all that apply)*

- B Diabetes (needing insulin)
- B Diabetes (do not need insulin)
- B High cholesterol/high blood lipids
- B High blood pressure
- B Ischemic (coronary) heart disease
- B Cerebrovascular disease (stroke)
- B Liver cancer
- B Lung cancer
- B Cancer of the digestive system
- B Breast cancer
- B Other cancers
- B Goiter / Thyroid abnormality
- B Epilepsy
- B Liver disease (not cancer)
- B Kidney disease
- B Depression / anxiety
- B Arthritis
- B Pneumonia
- B Chronic bronchitis/lung disease
- B Asthma
- B Malaria
- B Dengue fever
- B Tuberculosis
- B Other chronic infection
- B Other diseases not mentioned above
- B Never been to the doctor

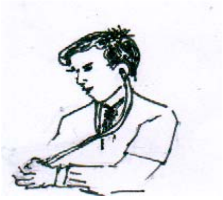

C14 Overall how would you rate your health during the past 4 weeks?

B Excellent B Very good B Good
B Fair B Poor B Very poor

C15 During the past 4 weeks, how much did physical health problems limit your usual physical activities (such as walking or climbing stairs)?

B Not at all B Very little B Somewhat
B Quite a lot B Could not do physical activities

C16 During the past 4 weeks, how much difficulty did you have doing your daily work, both at home and away from home, because of your physical pain?

B None at all B A little bit B Some
B Quite a lot B Could not do daily work

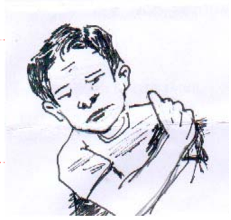

C17 How much bodily pain have you had during the past 4 weeks?

B None B Very mild B Mild
B Moderate B Severe B Very severe

C18 During the past 4 weeks, how much energy did you have?

B Very much B Quite a lot B Some
B A little B None

C19 During the past 4 weeks, how much did your physical health or emotional health problems limit your usual social activities with family or friends?

B Not at all B Very little B Somewhat
B Quite a lot B Could not do social activities

C20 During the past 4 weeks, how much have you been bothered by emotional problems (such as feeling anxious, depressed or irritable)?

B Not at all B Slightly B Moderately
B Quite a lot B Extremely

C21 During the past 4 weeks, how much did personal or emotional problems keep you from doing your usual work, school or other daily activities?

B Not at all B Very little B Somewhat
B Quite a lot B Could not do daily activities

C22 In the last 12 months how many serious injuries have you had that were enough to interfere with daily activities and/or required medical treatment?

B None ⟶ **please go to C28**
B once b twice b 3 times b 4 times or more

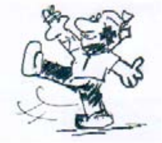

Please answer C23-C27 for your most serious injury during the last 12 months

C23 Where were you when you were seriously injured?

B Home
B Sports facility
B Workplace *(non-agricultural)*
B Road
B Workplace (*agricultural*)
B Other

C24 Was this serious injury related to road traffic?

B Yes B No ⟶ **please go to C26**

C25 If a traffic injury, what were the circumstances?

C25A What was your role?

B Driver B Passenger B Pedestrian → **skip to C25C**

C25B What type of vehicle were you driving or a passenger in?

B Bicycle B Motor-bike B Bus, van, tour coach
B Car/pickup B other eg. train, boat, airplane

C25C Who was the other party in this serious accident?

B Bicycle B Motor-bike B Bus, van, tour coach
B Car/pickup B other eg. train, boat, airplane
B other object that is not vehicle eg. tree, other obstruction

C26 If not a traffic injury, how were you seriously injured? *(please tick one box that fits best)*

b Assault (punch, push or kick) b Gun shot
b Fall (not pushed) b Stab/Cut
b Other blunt force b Fire, heat
b Drowning b Poisoning
b Bite or sting (animal, insect) b Other

C27 Was the serious injury? *(please tick one box that fits best)*

b Accidental b Non-accidental (involving another person)
b Non-accidental (no other person involved)

C28 In the past 12 months, have you personally used any of the following health services? *(please tick all that apply)*

B Government health centre B Community Hospital
B Government clinic B Provincial/other government hospital
B Private clinic B Private hospital
B Traditional healer *(Thai/ Chinese/other)* B Other
B None → **if 'none' go to Question C30**

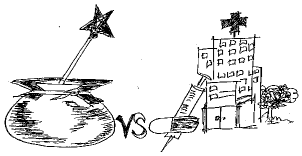

C29 Which of the following did you use in the past 12 months to cover costs of your medical treatment? *(please tick all that apply)*

B Non-government employer scheme B Civil servant/state enterprise benefit scheme
B Private health insurance B 30 Baht scheme
B Self-payment B Other

C30 In the past 12 months was there any occasion when you consider you should have used health services for an illness or injury, but you did not use the service?

B Yes b No ⟶ **if 'No' please go to C32**

C31 If yes, why did you not use the health services? *(please tick all that apply).*

B	Too expensive	B	Too difficult to travel to the service
B	Had to wait too long	B	Not satisfied with services
B	Scared of going	B	Don't like health provider (Doctor, nurse, other staff)
B	Could not get time off work/too busy at work	B	Could not get away from family commitments
B	Other		

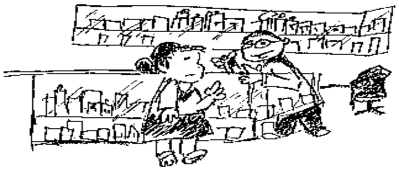

C32 Medication in the past 12 months:-
(Please tick ✓ to fit best for each question)

	Yes	No
Have you taken (eaten, injected, rubbed on etc.) medication prescribed by a medical doctor or other health personnel?		
Have you purchased any medication that did not require a doctor's prescription??		
Have you used traditional and/or herbal medicines		

D SOCIAL NETWORKS AND WELL-BEING

D1 How frequently you do each of the following activities? *(Please tick ✓ to fit best for each question)*

	**Everyday**	**Nearly/ every week**	**1-2 times/month**	**Very few**	**Never**
Spend time with parents or other relatives					
Spend time socially with neighbors					
Spend time socially with colleagues from work or your profession					
Spend time socially with other friends (old friends etc.)					
Spend time with people at your temple, mosque or other place of worship					
Spend time socially with people at recreational clubs or voluntary or service organization					
Participate in political parties, trade unions, environmental groups					

D2 Generally speaking, would you say that most people can be trusted or that you can't be too careful in dealing with people?

B Most people can be trusted   b You must be wary of people all the time

D3 How would you rate the support you are getting from the following:
(Please tick ✓ to fit best for each question)

	Very little support	A little support	Quite a bit of support	A lot of support	Not relevant
Your family?					
neighbours/local people?					
Local government officials?					
Religious groups?					
Friends?					
employer/ boss?					
others in the workplace?					

D4 What is your religion?

B Buddhism B Islam b Christianity b Hinduism b Other b None

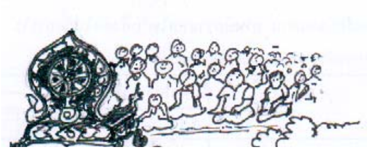

D5 In your life:- (*for each question, tick the box that fits best on the zero-to-ten scale*)

	Not at all ←→ Very much										
	0	1	2	3	4	5	6	7	8	9	10
how important is religion to calm your mind when facing problems?											
how important are spiritual practices *(eg sado clo or praying)*?											
how important is karma?											

D6 Thinking about your own life and personal circumstances, how satisfied are you with...

(tick box on zero-to-ten scale that fits best for each question)

	Completely Dissatisfied ←										→ Completely Satisfied
	0	1	2	3	4	5	6	7	8	9	10
…your standard of living?											
…your health?											
…what you achieve in life?											
…your personal relationships?											
…how safe you feel?											
…feeling part of your community?											
…your future security?											
…the neighbourhood where you live?											
…your religion or spirituality?											
…your life as a whole?											

D7 Have you had to go without things you really needed in the last 12 months because you were short of money?

B Yes, often   b Yes, sometimes   b No, never

E FOOD AND PHYSICAL ACTIVITY

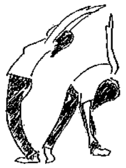

E1 How often, on average, do you eat each of the following foodstuffs?
(Please tick ✓ to fit best for each question)

	Never or less than once a month	1-3 times/ month	1-2 times/ week	3 - 6 times/ week	Once a day or more
Food/dessert with coconut milk					
Deep fried food					
Fermented food...fish/crab					
Roasted/smoked food eg. Thai sausage/roasted chicken					
Uncooked meat or shrimp					
Fermented fruit/vegetable					
Instant food					
Canned food					
Soft drink					
Milk eg. Fresh, carton or powder milk					
Soybean products eg. soya milk, tofu					
Food supplements, vitamins, minerals					

E2 Typically, how often do you eat in (or eat food from) each of the following?
(Please tick ✓ to fit best for each question)

**Source/location of food purchases**	**Never / Less than once a month**	**1-2 times /month**	**Once a week or more**	**Everyday / most days**
Restaurant where you pay more than 200 Baht/ person				
Food shop where you pay less than 100 Baht/ person				
Western-style Fast Food (e.g. burger, pizza)				
Work Canteen				
Street vendor / Market stall				
Home delivery (e.g. pizza)				
Food prepared at home				

E3 How important is each of the following in influencing your choice of foods?
(Please tick ✓ to fit best for each question)

	**Very important**	**Important**	**Of minor importance**	**Unimportant**
Cost – foods I can afford				
Health – try to provide healthy diet				
Taste – foods I like eating				
Convenience – ready to eat				
Limited cooking or storage facility				
Ability to carry and transport foods				
Culture – traditional foods				
Habit – what I usually have to eat				
Religion				
Food safety concerns				

**E4** How many serves of vegetables do you usually eat each day? *(one serve = ½ cup cooked or 1 cup uncooked)*	N N no. of serves
**E5** How many serves of fruit do you usually eat each day? *(one serve, equivalent to 1 cup of diced pieces)*	N N no. of serves

E6 During a typical week (7-day period), how many times on average do you do the following kinds of exercise? *(Enter '0' if you do not do any of that type of exercise)*

	**Times per Week**
Strenuous exercise (heart beats rapidly) for **more than 20 minutes**<br>*e.g. heavy lifting, digging, aerobics or fast bicycling, running, soccer, trakraw*	N N
Moderate exercise (not exhausting but breathe harder than normal) for **more than 20 minutes**<br>*e.g. carrying light loads, cycling at a regular pace,*	N N
Mild exercise (minimal effort) for **more than 20 minutes**<br>*e.g. yoga, Tai-Chi, bowling*	N N
Walking (non-stop) for **at least 10 minutes**<br>*e.g. at work, at home, exercise*	N N

E7 How often do you do household cleaning or gardening work?

b Seldom or Never B 1-3 times/ month

b Once or twice/ week b 3-4 times/ week

b Most days

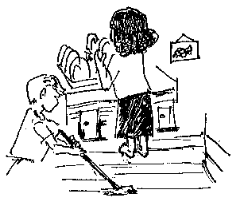

E8 How many hours per day in total do you usually spend:

**activities**	**duration**
Sleeping? (if you regularly sleep during the day include this also)	N N hours/day
Watching TV and/or playing computer games	N N hours/day
Sitting for <u>any</u> purpose *(eg reading, resting, working, thinking)?*	N N hours/day

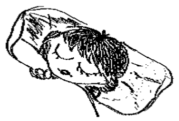

F TOBACCO, ALCOHOL AND TRANSPORT

F1 F1 Have you ever smoked?

B Yes b No ⟶ **If 'no' please go to question F5**

F2 At what age did you **start** smoking N N years

F3 If you have **quit** smoking, at what age did you stop? N N years b still smoking

F4 How many cigarettes do you smoke per day now (if current smoker), or did you smoke per day (if former smoker)? N N number per day

F5 Are you exposed on most days to smoke coming from other people who are smoking?
(Tick all that apply).

b Yes, at home
b Yes, in a recreation place
b Yes, in the workplace
b Yes, at public transport station eg. train/bus station
b Yes, other places
b No

F6 Have you ever drunk alcohol?

b Occasional social drinker
b No, never ⟶ **Please go to question F11**
b Current regular drinker
b Used to drink before, now **stopped**

F7 At what age did you start drinking alcohol? N N years

F8 If you have **quit** drinking, at what age did you stop? N N years b still drinking

F9 How many glasses of alcohol do/did you have in one sitting when you are/were drinking?

B less than 2 glasses
b 2-3 glasses
b 4-5 glasses
b 6 glasses or more

F10 During the last 12 months have you driven a motor vehicle after consuming 3 or more glasses of alcohol?

B Yes
b No
b Do not normally drive

F11 Which of the following do you, or any member of your household, own?
(Please tick all that apply)

B Bicycle
b Motorcycle
b Car/pick-up/van
B Truck
B Boat
B No vehicle

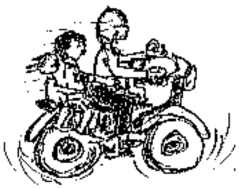

F12 How often do you use a safety belt when driving or sitting in a car?
(Please answer for both front and back seat)

Safety belt	Always wear safety belt	Sometimes wear safety belt	Never choose to wear safety belt	Vehicle doesn't have safety belt
Front Seat				
Back Seat				

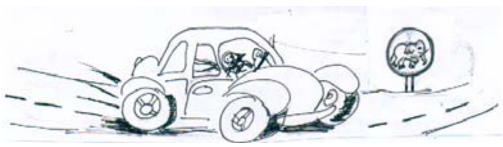

F13 How often do you wear a helmet when you travel on or ride a motorbike?

B Regularly b Sometimes

B Rarely or never b I do not ride motorbikes

F14 How often do you ride in the back of an open truck or pick-up?

B Regularly b Sometimes b Rarely/Never

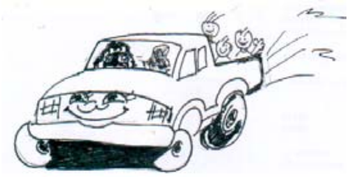

G YOUR FAMILY

G1 about your parents

**Mother**	**Father**
What is the highest grade of education completed by your mother?<br>B No formal education<br>b Primary level<br>B Secondary level<br>B Tertiary level<br>B Do not know	What is the highest grade of education completed by your father?<br>B No formal education<br>B Primary level<br>B Secondary level<br>B Tertiary level<br>B Do not know
Is your mother still alive?<br>No At what age did she die? N N years<br>B Yes What is her age? N N years	Is your father still alive?<br>b No At what age did he die? N N years<br>b Yes What is his age? N N years

If both of them are still alive → **skip to G3**

G2 What did your mother and/or father die from? *(you may tick* ☑ *more than one cause; leave column blank for the irrelevant box)*

	**Mother**	**Father**
Heart disease	b	b
High blood pressure	b	b
Stroke	b	b
Cancer	b	b
Tuberculosis	b	b
Pneumonia	b	b
Other infection	b	b

	**Mother**	**Father**
Diabetes	b	b
Kidney disease	b	b
Injury	b	b
Child birth	b	b
Old age	b	b
Other	b	b
Do not know	b	b

G3 Do you have children?

B Yes b No

If 'no' skip to G18The next section is for those who have had children

G4 How many daughters do you have now? N N

How many sons do you have now? N N

G5 Would you like to have more children?

B Yes

B No

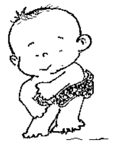

G6 Have you ever had a child who was born alive but later died?

B1 Yes

B2 No

G7 How old were you when your first child was born?

N N years

G8 Is your youngest child:

B Female

B Male

G9 How old is your youngest child now?

N N years and N N months

(ex. 3 years and 11 months) only child is considered as youngest child too.

G10 What is the size of your youngest child now?

Weight N N . N Kgs b Do not know

(eg 10.5 kgs)

Height N N N Cms b do not know

G11 What was the weight at birth of your youngest child? N N N N grams

b do not know

G12 Was this youngest child breastfed after birth?

B Yes

B No → **skip to G14**

G13 If Yes, how many months?

N N months

G14 Have any of your children ever been diagnosed with asthma?

B Yes

B No → **skip to G16**

G15 If Yes, which one(s)? *(Please tick all that apply)*

B 1st child B 3rd child

B 2nd child B 4th or later child

G16 Have any of your children ever had allergic sneezing, coughing, mucus without having a cold?

B Yes

B No → **skip to G18**

G17 If Yes, which one(s)? *(Please tick all that apply)*

B 1st child B 3rd child

B 2nd child B 4th or later child

The last question is about your pets

G18 Do you have the following pet animals at home? *(Please tick all that apply)*

B Dog b Cat

B Bird b Other

B Do not have pet animals

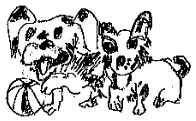

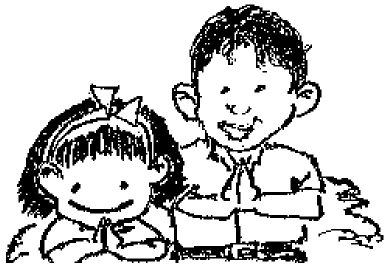

โครงการวิจัยสุขภาพ มหาวิทยาลัยสุโขทัยธรรมาธิราช ขอขอบคุณทุกท่านสำหรับความร่วมมือครั้งนี้

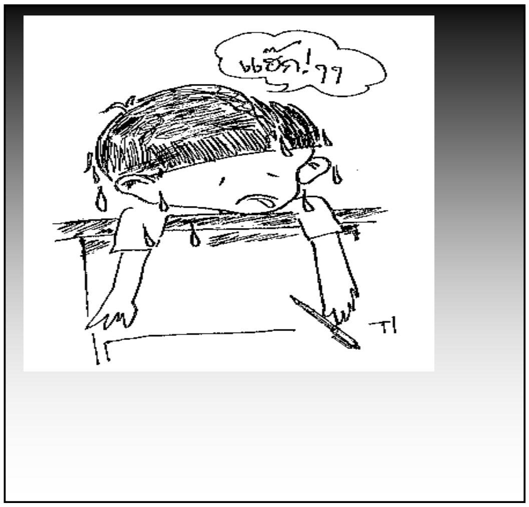

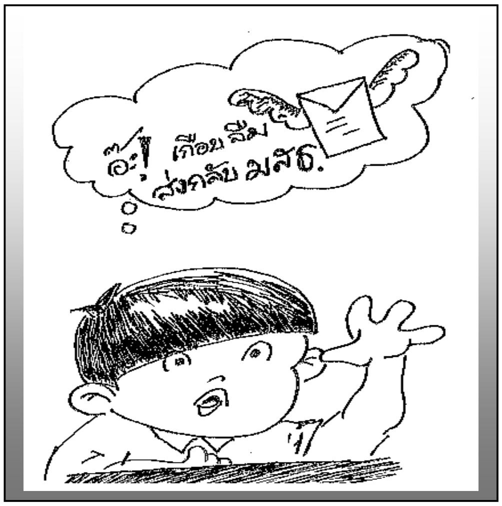

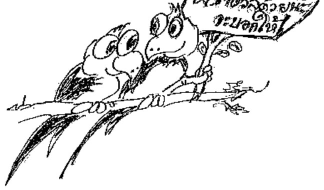

Supplement: Additional file 1 — Baseline 2005 Questionnaire (English). An English translation of the questionnaire sent to all 200,000 students enrolled at Sukhothai Thammathirat Open University in 2005 [file 1756-0500-4-166-S1.PDF]
