# Supplementary material for: Methods used for successful follow-up in a large scale national cohort study in Thailand
Source: BMC Res Notes. 2011 May 27;4:166. doi: 10.1186/1756-0500-4-166 (PMC3123220; doi:10.1186/1756-0500-4-166)

แบบสอบถาม

โครงการวิจัยสุขภาพ

รหัสประจำตัวนักศึกษา N N N N N N N N N N **SID**

รายละเอียดเกี่ยวกับผู้ตอบแบบสอบถาม

หมายเลขบัตรประจำตัวประชาชน N N N N N N N N N N N N N **PID**

ชื่อ..นามสกุล...

ที่อยู่ : เลขที่.....................................หมู่บ้าน.......................ซอย............................ถนน..............................

ตำบล/แขวง.....................................อำเภอ/เขต...จังหวัด..

รหัสไปรษณีย์ N N N N N **ZIP**

เบอร์โทรศัพท์ที่บ้าน..........................โทรศัพท์ที่ทำงาน...................โทรศัพท์มือถือ..

e-Mail……………………………………………………………………………

หน้านี้จะได้รับจัดเก็บเป็นความลับ แยกออกจากส่วนอื่น

บุคคลอื่นที่สามารถติดต่อได้ (กรณีที่ติดต่อท่านไม่ได้)

ชื่อ..นามสกุล...

ที่อยู่ : เลขที่............................หมู่บ้าน.......................ซอย................................ถนน...

ตำบล/แขวง..................................อำเภอ/เขต..................จังหวัด..................รหัสไปรษณีย์............................

เบอร์โทรศัพท์ที่บ้าน............................โทรศัพท์ที่ทำงาน..........................โทรศัพท์มือถือ................................

QID

รหัสแบบสอบถาม N N N N N N

****

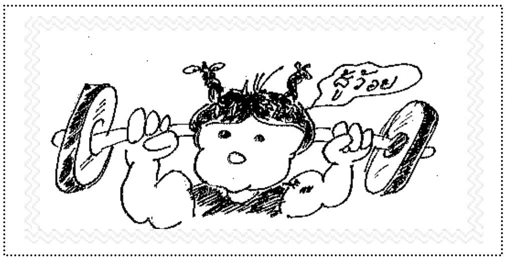

คำชี้แจง

แบบสอบถามสภาวะสุขภาพคนไทย ภายใต้โครงการ “ Thai Health-Risk Transition : A National Cohort Study” เพื่อศึกษาการเปลี่ยนผ่านของสุขภาพประชาชนไทย ให้เข้าใจถึงปัจจัยสุขภาพอันจะนำไปสู่ข้อเสนอเชิงนโยบายที่ส่งผลต่อสุขภาพที่ดีของประชาชนต่อไปในอนาคต โดยมีนักศึกษามหาวิทยาลัยสุโขทัยธรรมาธิราชเป็นตัวแทนของคนไทยทั่วประเทศไทย

ความสำเร็จของโครงการวิจัยสุขภาพนี้ ขึ้นกับการที่นักศึกษา มสธ. มีความร่วมมือและให้ข้อมูลที่ครบถ้วนสมบูรณ์ การเข้าร่วมโครงการวิจัยครั้งนี้เป็นไปโดยความสมัครใจ จะไม่มีผลใดๆ ทั้งสิ้นต่อการประเมินผลสัมฤทธิ์ทางการเรียนของนักศึกษา

ข้อมูลที่ได้ในแบบสอบถามนี้ จะถูก**รักษาไว้เป็นความลับ**และจะใช้เพื่อการวิจัยนี้เท่านั้น จะไม่เปิดเผยข้อมูลไม่ว่าบางส่วนหรือทั้งหมดโดยไม่ได้รับการอนุญาตจากผู้ตอบคำถาม

สำหรับข้อมูลเพิ่มเติมเกี่ยวกับโครงการวิจัยสุขภาพ หรือถ้าท่านมีประเด็นใดในเอกสารที่ไม่เข้าใจ โปรดติดต่อ รองศาสตราจารย์ ดร.สำอาง สืบสมาน โครงการวิจัยสุขภาพ ห้อง 101 อาคารตรีศร มสธ. โทรศัพท์/โทรสาร 02 504 7780 หรือ Email tcs@stou.ac.th หรือเยี่ยมชม website: http://www.stou.ac.th/ANU/

หากท่านยินดีเข้าร่วมเป็นสมาชิกโครงการวิจัยสุขภาพ โปรดลงชื่อในช่องข้างล่างนี้

ข้าพเจ้ายินดีเข้าร่วมโครงการวิจัยสุขภาพ “Thai Health-Risk Transition : A National Cohort Study”

ลงชื่อ ....

( )

วันที่ ..../.............../...............

สิทธิพิเศษสำหรับสมาชิกโครงการวิจัยสุขภาพ

ฟรี !

ภายใน 2 เดือนนับจากวันที่ท่านส่งแบบสอบถามชุดนี้กลับไปยัง มสธ. ท่านสามารถเข้าใช้ **MyDataBase** ซึ่งเป็นระบบฐานข้อมูลของท่านเองในอินเตอร์เน็ต ให้พื้นที่ฟรี 20MB ในนั้นท่านสามารถสร้าง Website ของตนเองได้ถึง 5 ชื่อ และเครื่องมือต่างๆ ให้ใช้อีกมากมาย เป็นระยะเวลา 1 ปี โดยเข้าไปที่ **http://www.tcsstou.net** แล้วใช้ Username เป็นรหัสประจำตัวนักศึกษาของท่าน ส่วน Password นั้น ใช้ตัวเลข 8 หลักที่เป็นวันเดือนปีเกิดของท่าน (คำตอบข้อ A1) เช่นเกิด 5 มี.ค. 2512 ใช้ Password เป็น 05032512 เป็นต้น แล้วกรุณาเปลี่ยน Password ทันทีที่เข้าใช้ครั้งแรก เพื่อรักษาความลับของท่าน

นอกจากนี้ เรายังมีรายการพิเศษให้ท่านร่วมสนุกอีก โปรดพลิกดูด้านหลังปก

คำชี้แจง คำถามที่เป็นตัวเลือก โปรดกาเครื่องหมายถูก (✓) ลงในช่อง b หน้าตัวเลือก ให้มีภาพเป็น b✓ โดยกาได้เพียงคำตอบเดียว ยกเว้นข้อที่มีระบุว่า "*(เลือกได้มากกว่าหนึ่งคำตอบ)*" ไว้ที่ท้ายคำถาม ส่วนคำถามที่ให้เขียนคำตอบเป็นตัวเลขนั้น โปรดเขียนตัวเลขบรรจงลงภายในช่อง N ช่องละหนึ่งตัวเลข โปรดใช้ปากกาสีเข้ม (ดำ หรือน้ำเงิน)

A ข้อมูลเกี่ยวกับตัวท่านและบ้านของท่าน

A1 ท่านเกิดเมื่อ (กรุณากรอกเป็นตัวเลขเท่านั้น)

A1 N N / N N / N N N N

วันที่ เดือน ปี พ.ศ.

(ตัวเลขทั้ง 8 ตัวนี้ จะใช้เป็นรหัสผ่านของท่าน เพื่อเข้าใช้ MyDataBase ฟรี ที่ http://www.tcsstou.net ในอีก 2 เดือน นับจากวันที่ท่านส่งฟอร์มนี้กลับ มสธ.)

A2 เพศ A2 b 1 ชาย b 2 หญิง

A3 ท่านมีพี่ชายน้องชายทั้งหมด (*ไม่รวมตัวท่าน*)

จำนวน N N คน A3

A4 ท่านมีพี่สาวน้องสาวทั้งหมด (*ไม่รวมตัวท่าน*)

จำนวน N N คน A4

A5 ในพี่น้องทั้งหมด ท่านเป็นบุตรลำดับที่ A5 N N

A.6 การศึกษาสูงสุดของท่าน ***(ไม่รวมการศึกษา มสธ. ขณะนี้)***

A6
- B 1 ม3. หรือเทียบเท่า
- B 2 ม.6 /ปวช. หรือเทียบเท่า
- b 3 ป.วิชาชีพเทคนิค/ชั้นสูง/อนุปริญญา
- b 4 ปริญญาตรี หรือสูงกว่า

A7 ท่านคิดว่าท่านมีเชื้อสายหรือวัฒนธรรมที่เชื่อมโยงกับกลุ่มใดบ้าง (*เลือกได้มากกว่าหนึ่งคำตอบ*)

- A7_1 b จีน
- A7_2 b มอญ
- A7_3 b ไทยกลาง
- A7_4 b ไทยอีสาน
- A7_5 b ชาวเหนือ
- A7_6 b ชาวใต้
- A7_7 b อื่นๆ

ค่าของตัวแปร (0 = ไม่เลือก; 1 = เลือก)

A8 สถานภาพของท่านในปัจจุบัน

A8
- b 1 โสด ⟶ **ข้ามไปตอบข้อ A11**
- b 2 อยู่กับคู่ครอง (ยังไม่แต่งงาน) ⟶ **ข้ามไปตอบข้อ A11**
- b 3 มีคู่สมรสแล้ว

A9 สถานภาพสมรสของท่านในปัจจุบัน (*เลือกเพียง 1 ข้อ*)

A9
- B 1 แต่งงานครั้งแรกและครั้งเดียว
- b 2 แต่งงานครั้งที่ 2 หรือมากกว่า
- b 3 แยกกันแล้ว (แต่ไม่ได้หย่า)
- b 4 หย่า
- b 5 หม้าย (คู่สมรสเสียชีวิต)

A10 ท่านแต่งงานครั้งแรกเมื่ออายุ A10 N N ปี

คำถามต่อไปนี้ (ข้อ **A11**-เป็นต้นไป) เกี่ยวกับ**"บ้าน"** หมายถึง กรณีใดกรณีหนึ่งต่อไปนี้

- ถ้าท่านมีบ้านหลักเพียงแห่งเดียว หรือถ้ากลับมาอยู่บ้านเดือนละ 4 วันขึ้นไป โปรดตอบเกี่ยวกับ**บ้านหลักของครอบครัว**ของท่าน
- **แต่**ถ้าท่านกลับมาเยี่ยมบ้านนานๆครั้ง หรือน้อยกว่า เดือนละ 4 วัน โปรดตอบเกี่ยวกับ**ที่พักอาศัย**สำหรับเวลาไปทำงานของท่าน

รหัสแบบสอบถาม QID N N N N N N

A11 นอกจาก**บ้านหลักของครอบครัว**ของท่านแล้ว ท่านยังมีที่พักอาศัยอื่น เวลาไปทำงานหรือไม่

A11
- b 1 มี
- b 2 ไม่มี → **ข้ามไปตอบข้อ A13**

A12 ท่านพักอยู่ที่บ้านหลักของครอบครัวโดยเฉลี่ย เดือนละ N N วัน **A12**

A13 *ในช่วง <u>5 ปีที่ผ่านมา</u>* ท่านเคยย้ายบ้านที่เป็นบ้านหลักของครอบครัวของท่านหรือไม่

A13 b 1 เคย b 2 ไม่เคย → **ข้ามไปตอบข้อ A 15**

A14 การย้ายบ้านครั้งล่าสุดของท่านเป็นการย้ายที่ตรงกับข้อใดมากที่สุด

A14
- b 1 จากชนบท ไปอยู่ ในชนบท
- b 2 จากชนบท ไปอยู่ ในเมือง
- b 3 จากเมือง ไปอยู่ ในเมือง
- b 4 จากเมือง ไปอยู่ ในชนบท

A15 หัวหน้าครอบครัวในบ้านของท่านคือ

A15
- b 1 ท่านเอง
- b 2 ภรรยา (หรือสามี) ของท่าน
- b 3 บิดาของท่าน
- b 4 มารดาของท่าน
- b 5 บิดาของคู่สมรสของท่าน
- b 6 มารดาของคู่สมรสของท่าน
- b 7 ญาติ/บุคคลอื่นที่เป็นชาย
- b 8 ญาติ/บุคคลอื่นที่เป็นหญิง

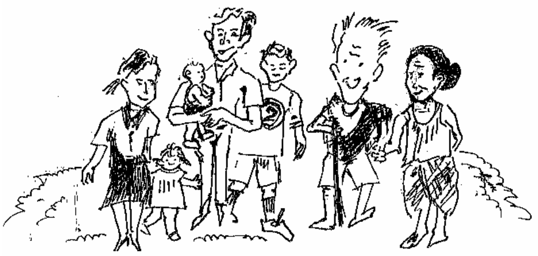

A16 สมาชิกในบ้านของท่าน (*รวมทั้งตัวท่าน*)
มีทั้งหมด จำนวน N N คน **A16**

A17 สมาชิกในบ้านของท่าน ที่มีอายุ 15 ปีหรือต่ำกว่า
มีกี่คน จำนวน N N คน **A17**

ถ้าไม่มี โปรดใส่เลข 0 ในช่อง N N *ที่ให้ไว้ ดังนี้* N N

A18 สมาชิกในบ้านของท่าน มีบุคคลต่อไปนี้อาศัยอยู่ ด้วยหรือไม่ (*เลือกได้มากกว่าหนึ่งคำตอบ*)

- **A18_1** b คู่สมรส/คู่ครอง
- **A18_2** b บุตร (อายุตั้งแต่ 16 ปีขึ้นไป)
- **A18_3** b พี่น้อง (อายุตั้งแต่ 16 ปีขึ้นไป)
- **A18_4** b บิดามารดา
- **A18_5** b ปู่ ย่า ตา ยาย
- **A18_6** b ญาติอื่นๆ/เขย/สะใภ้
- **A18_7** b บุคคลผู้อื่นที่ไม่ใช่ญาติ

ค่าของตัวแปร (0 = ไม่เลือก; 1 = เลือก)

A19 ลักษณะบ้านหรือที่พักอาศัยของท่าน ตรงกับข้อใดมากที่สุด

A19
- b 1 บ้านเดี่ยว
- b 2 บ้านแฝด
- b 3 ตึกแถว/บ้านที่มีหน้าร้านขายของ
- b 4 ทาวน์เฮ้าส์
- b 5 หอพัก แฟลต/อพาร์ตเมนท์/คอนโด
- b 6 อื่นๆ

A20 ลักษณะความเป็นเจ้าของบ้านหรือที่พักอาศัยของท่าน

A20
- b 1 ท่านหรือคู่สมรสเป็นเจ้าของ
- b 2 เช่าซื้อ/กู้ยืม/ติดจำนอง
- b 3 ไม่ได้เป็นเจ้าของ (*แต่อยู่โดยไม่ต้องเสียค่าเช่า เช่นอาศัยบ้านบิดามารดา*)
- b 4 เช่า
- b 5 อื่น ๆ

A21 ในรัศมี 1 กิโลเมตรจากที่พักอาศัยของท่าน สิ่งเหล่านี้เป็นปัญหามากน้อยเพียงใด

(*กาเครื่องหมายถูก* ✓ *ลงช่องที่ตรงตามความคิดเห็นของท่าน*)

	เป็นปัญหามาก	เป็นปัญหาเล็กน้อย	ไม่เป็นปัญหา	ไม่ทราบ
มลพิษทางอากาศ **A21N1**	1	2	3	4
มลพิษทางน้ำ/น้ำเสีย **A21N2**	1	2	3	4
เสียงรบกวน **A21N3**	1	2	3	4
สารเคมีอุตสาหกรรม **A21N4**	1	2	3	4
สารเคมีทางการเกษตร **A21N5**	1	2	3	4
สารเคมีป้องกันกำจัดศัตรูพืชและสัตว์ **A21N6**	1	2	3	4
กองขยะทิ้งเกลื่อนกลาด **A21N7**	1	2	3	4
กลิ่นเหม็นรบกวน **A21N8**	1	2	3	4
การบุกรุกเข้าไปในบ้าน/ การลักขโมย **A21N9**	1	2	3	4
การเดินทางด้วยระบบขนส่งมวลชนไม่ **A21N10**	1	2	3	4
ขาดทางเดินเท้า **A21N11**	1	2	3	4
ขาดพื้นที่พักผ่อนหย่อนใจ **A21N12**	1	2	3	4
โอกาสเสี่ยงกับการถูกสุนัขกัด **A21N13**	1	2	3	4

คำถามต่อไปนี้เกี่ยวข้องกับสภาพแวดล้อมในการดำเนินชีวิตของท่านใน**ปัจจุบัน**และสภาพแวดล้อมที่ท่านอาจจำได้เมื่อยังอยู่ใน**วัยเด็กช่วงอายุ 10 - 12** ซึ่งจะส่งผลทำให้เข้าใจการเปลี่ยนแปลงในช่วงเวลาที่ผ่านไป

A22 ที่พักอาศัยของท่านทั้งปัจจุบัน และในวัยเด็ก

ที่พักอาศัยถาวรของท่านใน<u>ปัจจุบัน</u>อยู่บริเวณใด þ1 ในชนบท þ2 ในเมือง **A22A**

ที่พักอาศัยถาวรใน<u>วัยเด็ก</u>ของท่านอยู่บริเวณใด þ1 ในชนบท þ2 ในเมือง **A22B**

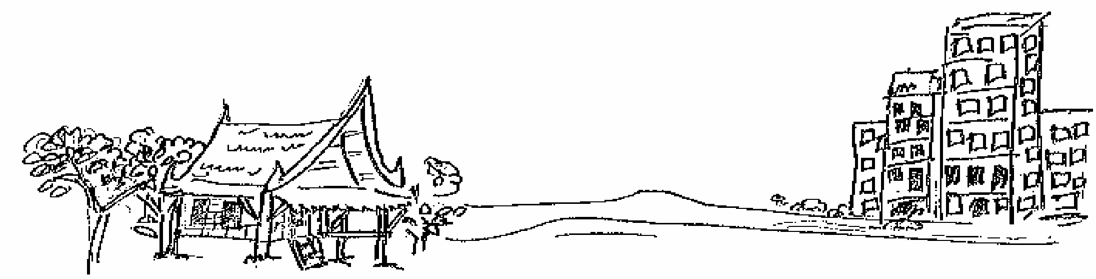

คำถามเกี่ยวกับช่วงวัยเด็ก

A23 ในช่วง**วัยเด็ก10-12 ขวบ (ชั้นประถม 4 - 6)** ที่บ้านของท่านมีอุปกรณ์เครื่องใช้ต่อไปนี้หรือไม่

(เลือกได้มากกว่าหนึ่งคำตอบ) **ค่าของตัวแปร (0 = ไม่เลือก; 1 = เลือก)**

**A23_1**	B	ไฟฟ้า- ผลิตเอง (เครื่องปั่นไฟ)	B	วิทยุ	**A23_9**
**A23_2**	B	ไฟฟ้า-สายไฟจากภายนอก	B	คอมพิวเตอร์	**A23_10**
**A23_3**	B	เตาไมโครเวฟ	B	โทรศัพท์	**A23_11**
**A23_4**	B	ตู้เย็น	B	โทรศัพท์มือถือ	**A23_12**
**A23_5**	B	พัดลม	B	เครื่องทำน้ำอุ่น	**A23_13**
**A23_6**	B	เครื่องปรับอากาศ	B	เครื่องซักผ้า	**A23_14**
**A23_7**	B	โทรทัศน์	B	มุ้ง (มุ้งกันยุงสำหรับที่นอน)	**A23_15**
**A23_8**	B	วีดีโอ/ เครื่องเล่น เทป/ซีดี	B	มุ้งลวด	**A23_16**
			B	ไม่มีอุปกรณ์ใดตามที่กล่าวข้างต้น	**A23_17**

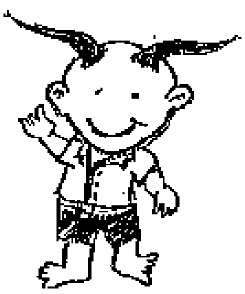

A24 ในช่วง**วัยเด็ก10-12 ขวบ (ชั้นประถม 4 - 6)** บ้านของท่านใช้**น้ำดื่มจากแหล่งใด** <u>มากที่สุด</u> (แหล่งน้ำที่ท่านใช้ จะต่อเชื่อมกับอุปกรณ์กรองน้ำหรือไม่ก็ตาม)

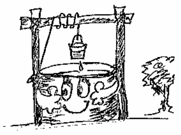

A24

B 1	น้ำประปา	B 4	น้ำจากแม่น้ำ ลำธาร คลอง บึง
B 2	น้ำบ่อ/น้ำใต้ดิน	B 5	น้ำบรรจุขวด
B 3	น้ำฝน	B 6	น้ำจากแหล่งกระจายน้ำอื่นๆ (เช่นตู้หยอดเหรียญ)

คำถามเกี่ยวกับปัจจุบันของท่าน

A25 **ปัจจุบัน**ในบ้านของท่านมีอุปกรณ์เครื่องใช้ต่อไปนี้หรือไม่ *(เลือกได้มากกว่าหนึ่งคำตอบ)*

**A25_1**	B	ไฟฟ้า- ผลิตเอง (เครื่องปั่นไฟ)	B	วิทยุ	**A25_9**
**A25_2**	B	ไฟฟ้า-สายไฟจากภายนอก	B	คอมพิวเตอร์	**A25_10**
**A25_3**	B	เตาไมโครเวฟ	B	โทรศัพท์	**A25_11**
**A25_4**	B	ตู้เย็น	B	โทรศัพท์มือถือ	**A25_12**
**A25_5**	B	พัดลม	B	เครื่องทำน้ำอุ่น	**A25_13**
**A25_6**	B	เครื่องปรับอากาศ	B	เครื่องซักผ้า	**A25_14**
**A25_7**	B	โทรทัศน์	B	มุ้ง (มุ้งกันยุงสำหรับที่นอน)	**A25_15**
**A25_8**	B	วีดีโอ/ เครื่องเล่นเทป/ ซีดี	B	มุ้งลวด	**A25_16**
		**ค่าของตัวแปร (0 = ไม่เลือก; 1 = เลือก)**	B	ไม่มีอุปกรณ์ที่กล่าวมาทั้งหมด	**A25_17**

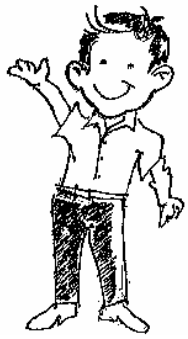

A26 **ปัจจุบัน** บ้านของท่านใช้**น้ำดื่มจากแหล่งใด** <u>มากที่สุด</u> (แหล่งน้ำนี้จะต่อเชื่อมกับอุปกรณ์กรองน้ำหรือไม่ก็ตาม)

A26

B 1	น้ำประปา	B 4	น้ำจากแม่น้ำ ลำธาร คลอง บึง
B 2	น้ำบ่อ/น้ำใต้ดิน	B 5	น้ำบรรจุขวด
B 3	น้ำฝน	B 6	น้ำจากแหล่งกระจายน้ำอื่นๆ (เช่นตู้หยอดเหรียญ)

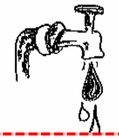

B รายได้ และอาชีพการงาน

B1 ท่านมีรายได้ประมาณเดือนละเท่าไร

B1

B 1 น้อยกว่า 3,000 บาท	B 2 3,001 -7,000 บาท	B 3 7,001 -10,000 บาท
B 4 10,001 -20,000 บาท	B 5 20,001 -30,000 บาท	B 6 มากกว่า 30,000 ขึ้นไป

B2 งานที่ท่านทำในปัจจุบัน เป็นงานที่ก่อให้เกิดรายได้หรือไม่

B2 B 1 งานที่มีรายได้ → **ข้ามไปตอบข้อ B4** b 2 งานที่ไม่มีรายได้

B3 กรณีเป็นงานที่ไม่มีรายได้ ข้อใดตรงกับท่านมากที่สุด

B3

B 1 ดูแลจัดการบ้าน/แม่บ้าน	B 5 กำลังหางานทำเป็นครั้งแรก
B 2 ช่วยธุรกิจในครอบครัวโดยไม่ได้รับค่าจ้าง	B 6 ว่างงาน
B 3 เกษียณแล้ว (และไม่ได้ทำงานที่มีรายได้)	B 7 เป็นนักศึกษา (ไม่ได้ทำงาน)
B 4 ทำงานไม่ได้เนื่องจากการเจ็บป่วย/ทุพลภาพ	B 8 อื่นๆ

สำหรับท่านที่ทำงานที่ไม่มีรายได้ เมื่อตอบข้อ B3 แล้ว ข้ามไปตอบข้อ C1

B4 ลักษณะงานหลักของท่านตรงกับข้อใดมากที่สุด

B4

- b 1 ข้าราชการ/ลูกจ้าง/พนักงานของรัฐ/รัฐวิสาหกิจ
- b 2 ลูกจ้างเอกชน
- b 3 นายจ้าง → **ข้ามไปตอบข้อ B7**
- b 4 ทำงานส่วนตัว (ไม่มีลูกจ้างและไม่เกี่ยวกับเกษตรกรรม) → **ข้ามไปตอบข้อ B7**
- b 5 ทำงานในที่นา/ไร่/สวน ของตนเอง → **ข้ามไปตอบข้อ B7**
- b 6 อื่นๆ → **ข้ามไปตอบข้อ B7**

B5 การทำงานเป็นข้าราชการ/ลูกจ้าง/พนักงานของรัฐ/รัฐวิสาหกิจ หรือลูกจ้างเอกชนของท่าน มีลักษณะใด

B5

- B 1 เป็นการจ้างที่มีลักษณะถาวร
- b 2 เป็นการจ้างเป็นสัญญาระยะยาว (เช่น 1 ปี หรือมากกว่า)
- b 3 เป็นการจ้างชั่วคราว
- b 4 อื่นๆ

B6

B6 งานที่ก่อรายได้ที่ท่านทำในปัจจุบัน ใช้เวลาทำงานประมาณสัปดาห์ละ N N ชั่วโมง

B7 งานที่ก่อรายได้มีลักษณะการทำงานที่เป็นกะกลางคืนหรือกะวันเสาร์/อาทิตย์หรือไม่

B7 b 1 เป็น b 2 ไม่เป็น

B8 ท่านรู้สึกมั่นคงในอนาคตการทำงานหรืออาชีพที่ทำอยู่ขณะนี้เพียงใด

B8
b 1 ไม่มั่นคงเลย b 2 มั่นคงเล็กน้อย
b 3 มั่นคง b 4 มั่นคงมาก

B9 ท่านต้องทำงานหลัง 6 โมงเย็น บ่อยครั้งแค่ไหน *(ที่เป็นงานล่วงเวลาทั้งที่ได้หรือไม่ได้ค่าตอบแทน)*

B9
b 1 5-7 วันต่อสัปดาห์ b 2 2-4 วันต่อสัปดาห์
b 3 1-4 ครั้งต่อเดือน b 4 น้อยกว่าข้างต้น
b 5 ไม่เคย

B10 ท่านใช้เวลาในการเดินทางระหว่างบ้านกับที่ทำงานโดยเฉลี่ยแล้ว (เที่ยวเดียว) นานเท่าใด

B10
b 1 น้อยกว่า 30 นาที b 2 30 นาที - 1 ชั่วโมง
b 3 1-2 ชั่วโมง b 4 2 -3 ชั่วโมง
b 5 มากกว่า 3 ชั่วโมง b 6 ไม่ต้องเดินทาง

B11 ท่านคิดว่าข้อความใดอธิบายลักษณะงานที่ก่อให้เกิดรายได้หลักของท่าน *(เลือกได้มากกว่าหนึ่งคำตอบ)*

B11_1 B นักวิชาการ (เช่น นักบัญชี แพทย์) b ช่างชำนาญงาน (เช่น ช่างไม้ **B11_2**
B11_3 b ผู้บริหารอาวุโส b ผู้บริหารระดับกลาง **B11_4**
B11_5 b เจ้าหน้าที่ในสำนักงาน b ผู้ใช้แรงงาน **B11_6**
B11_7 b อื่นๆ **ค่าของตัวแปร (0 = ไม่เลือก; 1 = เลือก)**

B12 สถานการณ์ในการทำงานของท่านต่อไปนี้ *(กาเครื่องหมายถูก ✓ ลงช่องที่ตรงตามที่ท่านทำจริง)*

สถานการณ์การทำงาน	บ่อยครั้ง	บางครั้ง	น้อยครั้ง	ไม่เคย	
ท่านมีส่วนร่วมในการตัดสินใจเกี่ยวกับงาน	1	2	3	4	B12N1
เวลาทำงานของท่านสามารถปรับเปลี่ยนได้	1	2	3	4	B12N1
ท่านต้องทำงานซ้ำซากเป็นประจำ	1	2	3	4	B12N1
ท่านมีเวลาพอเพียงในการทำงานทุกอย่าง	1	2	3	4	B12N1
ท่านต้องทำงานอย่างรวดเร็ว	1	2	3	4	B12N1

B13 ในช่วง 12 เดือนที่ผ่านมา ท่านมีประสบการณ์ดังต่อไปนี้บ่อยแค่ไหน ในที่ทำงานของท่าน

(กาเครื่องหมายถูก ✓ ลงช่องที่ตรงตามที่ท่านประสบจริง)

สภาพแวดล้อม	บ่อยครั้ง	บางครั้ง	น้อยครั้ง	ไม่เคย	ไม่ทราบ	
การสั่นสะเทือนจากการใช้เครื่องมือ เครื่องจักร	1	2	3	4	5	B13N1
เสียงดังจนต้องตะเบ็งเสียงพูดกับผู้อื่น	1	2	3	4	5	B13N2
ร้อนมากจนทำให้อึดอัด	1	2	3	4	5	B13N3
อากาศเย็นเกินไป	1	2	3	4	5	B13N4
มีละออง ควัน ฝุ่น/สารอันตราย (เช่น สารเคมี/ติดเชื้อ)	1	2	3	4	5	B13N5
จับต้อง หรือสัมผัสผลิตภัณฑ์หรือสารอันตราย	1	2	3	4	5	B13N6

C สุขภาวะ การบาดเจ็บ และการใช้บริการทางการแพทย์

C1 ท่านมีน้ำหนัก N N N กิโลกรัม C1

C2 ท่านมีส่วนสูง N N N เซ็นติเมตร *(โดยไม่ใส่รองเท้า)* C2

C3 จากการบอกเล่าของญาติผู้ใหญ่ ข้อความใด ตรงกับขนาดหรือน้ำหนักเมื่อแรกเกิดของท่านมากที่สุด

C3
- B 1 ตัวเล็ก หรือน้ำหนักน้อย
- b 2 ขนาดปกติ หรือน้ำหนักปกติ
- b 3 ตัวใหญ่ หรือน้ำหนักมาก
- b 4 ไม่ทราบ → **ข้ามไปตอบข้อ C5**

C4 หากท่านทราบ น้ำหนักแรกเกิดของท่านคือ N N N N กรัม
C4

C5 จากการบอกเล่าของญาติผู้ใหญ่ของท่าน ขณะที่เป็นเด็กทารก ท่านได้ดื่มนมมารดาหรือไม่

C5
- b 1 ได้ดื่ม
- b 2 ไม่ได้ดื่ม → **ข้ามไปตอบข้อ C7**
- b 3 ไม่ทราบ → **ข้ามไปตอบข้อ C7**

C6 ถ้าได้ดื่มนมมารดา ท่านได้ดื่มนานประมาณ N N เดือน
C6

C7 ข้อความใด ตรงกับสภาพทางสายตาในปัจจุบันของท่านที่สุด

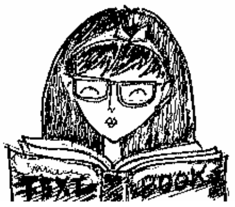

C7
- b 1 ไม่จำเป็นต้องใช้แว่นสายตา
- b 2 จำเป็นต้องใช้แว่นสายตาหรือคอนแทคเลนส์ตั้งแต่เด็ก (อายุต่ำกว่า13ปี)
- b 3 จำเป็นต้องใช้แว่นสายตาหรือคอนแทคเลนส์ตั้งแต่เป็นวัยรุ่น (อายุ 13-19 ปี)
- b 4 จำเป็นต้องใช้แว่นสายตาหรือคอนแทคเลนส์ตั้งแต่เป็นผู้ใหญ่ (อายุ 20 ปีขึ้นไป)

C8 ท่านมีปัญหาเกี่ยวกับสายตาที่ไม่สามารถแก้ไขได้ด้วยการใส่แว่น/คอนแทคเลนส์หรือไม่ *(เช่น ตาต้อ ฯลฯ)*

C8 b 1 มี b 2 ไม่มี

C9 ข้อความใด ตรงกับการได้ยินในปัจจุบันของท่านมากที่สุด (โดยไม่ต้องใช้เครื่องช่วยฟัง)

C9
- B 1 ได้ยินชัดเจน
- b 2 มีปัญหาในการได้ยินบ้าง เริ่มมีอาการตั้งแต่เด็ก (อายุต่ำกว่า13ปี)
- b 3 มีปัญหาในการได้ยินบ้าง เริ่มมีอาการตั้งแต่เป็นวัยรุ่น/ผู้ใหญ่ (อายุ 13 ปีขึ้นไป)
- b 4 หูหนวกตั้งแต่เด็ก (อายุต่ำกว่า13ปี)
- b 5 หูหนวกตั้งแต่เป็นวัยรุ่น/ผู้ใหญ่ (อายุ 13 ปีขึ้นไป)

C10 ผู้ใหญ่มีฟันแท้ทั้งสิ้น 32 ซี่ ขณะนี้ท่านมีฟันแท้จำนวนกี่ซี่

C10
- b 1 ไม่มีเลย
- b 2 1-5 ซี่
- b 3 6-19 ซี่
- b 4 20 ซี่หรือมากกว่า

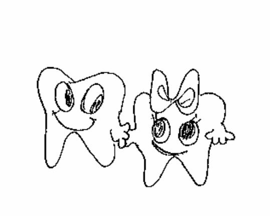

C11 ในปัจจุบัน ฟันแท้หรือฟันปลอมของท่านทำให้ท่านมีอาการต่อไปนี้หรือไม่ *(เลือกได้มากกว่า 1 ข้อ)*

- **C11_1** b ไม่สะดวกเวลาพูด
- **C11_2** b กลืนไม่สะดวก
- **C11_3** b เคี้ยวไม่สะดวก
- **C11_4** b ขาดความมั่นใจเมื่อเข้าสังคม
- **C11_5** b รู้สึกเจ็บปวด
- **C11_6** b ไม่มีอาการดังกล่าว

ค่าของตัวแปร (0 = ไม่เลือก; 1 = เลือก)

C12 ในช่วง 4 สัปดาห์ที่ผ่านมา ท่านเกิดความรู้สึกต่อไปนี้บ่อยแค่ไหน *(กาเครื่องหมายถูก ✓ ลงช่องที่ตรงตามที่ท่านทำจริง)*

ความรู้สึก		ตลอดเวลา	เกือบตลอดเวลา	บางเวลา	น้อยครั้งมาก	ไม่เคยเลย
รู้สึกกังวล	**C12N1**	1	2	3	4	5
รู้สึกกระวนกระวายหรือหงุดหงิด	**C12N2**	1	2	3	4	5
รู้สึกว่าต้องใช้ความพยายามในการทำทุกสิ่งทุก	**C12N3**	1	2	3	4	5

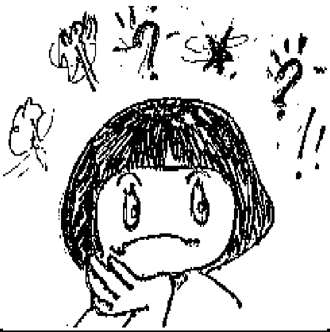

ค่าของตัวแปร (0 = ไม่เลือก; 1 = เลือก)

C13 ท่านเคยได้รับการ**วินิจฉัยโดยแพทย์**ว่าป่วยเป็นโรคใดต่อไปนี้ *(เลือกได้มากกว่าหนึ่งคำตอบ)*

C13_1	B	เบาหวาน (ต้องใช้อินซูลิน)	C13_14	B	โรคเกี่ยวกับตับ (ไม่ใช่มะเร็ง)
C13_2	B	เบาหวาน (ไม่ต้องใช้อินซูลิน)	C13_15	B	โรคเกี่ยวกับไต
C13_3	B	โคเลสเตอรอลสูงหรือไขมันในเลือ	C13_16	B	ซึมเศร้า/วิตกกังวล
C13_4	B	ความดันโลหิตสูง	C13_17	B	ข้ออักเสบ
C13_5	B	โรคหัวใจขาดเลือด	C13_18	B	ปอดบวม(นิวมอเนีย)
C13_6	B	โรคหลอดเลือดในสมอง (Stroke)	C13_19	B	หลอดลมอักเสบเรื้อรัง/โรคปอดอื่นๆ
C13_7	B	มะเร็งตับ	C13_20	B	หอบหืด
C13_8	B	มะเร็งปอด	C13_21	B	มาเลเรีย (ไข้จับสั่น)
C13_9	B	มะเร็งของระบบทางเดินอาหาร	C13_22	B	ไข้เลือดออก
C13_10	B	มะเร็งเต้านม	C13_23	B	วัณโรค
C13_11	B	มะเร็งอวัยวะอื่นๆ	C13_24	B	โรคติดเชื้อเรื้อรังอื่นๆ
C13_12	B	คอพอก/ต่อมไทรอยด์ผิดปกติ	C13_25	B	เป็นโรคอื่นที่นอกเหนือจากที่กล่าวข้างต้น
C13_13	B	ลมบ้าหมู	C13_26	B	ไม่เคยไปพบแพทย์เลย

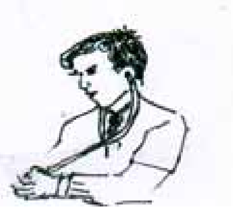

C14 ในช่วง 4 สัปดาห์ที่ผ่านมา โดยทั่วไปท่านประเมินสุขภาพของท่าน ว่าอย่างไร

C14
- B 1 ดีที่สุด
- B 2 ดีมาก
- B 3 ดี
- B 4 พอใช้
- B 5 แย่
- B 6 แย่มาก

C15 ในช่วง 4 สัปดาห์ที่ผ่านมา ปัญหาสุขภาพกายจำกัดกิจกรรมทางกายตามปกติของท่าน (*เช่น การ เดิน หรือการขึ้นลงบันได*) มากน้อยแค่ไหน

C15
- B 1 ไม่เลย
- B 2 น้อยมาก
- B 3 พอสมควร
- B 4 ค่อนข้างมาก
- B 5 ไม่สามารถทำกิจกรรมทางกายต่างๆ ได้

C16 ในช่วง 4 สัปดาห์ที่ผ่านมา ท่านมีความลำบากในการทำงานประจำวันทั้งงานในบ้าน และนอกบ้าน ที่เป็นผลมาจากสุขภาพทางกายของท่านมากน้อย แค่ไหน

C16
- B 1 ไม่มีเลย
- B 2 มีน้อยมาก
- B 3 มีพอสมควร
- B 4 มีค่อนข้างมาก
- B 5 ไม่สามารถทำงานประจำวันได้

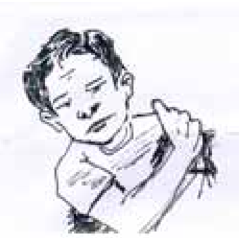

C17 ในช่วง 4 สัปดาห์ที่ผ่านมา ท่านมีความเจ็บปวดทางร่างกายมากน้อยแค่ไหน

C17
- B 1 ไม่มีเลย
- B 2 น้อยมาก
- B 3 น้อย
- B 4 ปานกลาง
- B 5 รุนแรง
- B 6 รุนแรงมาก

C18 ในช่วง 4 สัปดาห์ที่ผ่านมา ท่านมีความรู้สึกกระฉับกระเฉง มากน้อย แค่ไหน

C18

- 1 มีมาก
- 2 มีค่อนข้างมาก
- 3 มีบ้าง
- 4 มีเล็กน้อย
- 5 ไม่มีเลย

C19 ในช่วง 4 สัปดาห์ที่ผ่านมา สุขภาพทางกายหรือ ปัญหาด้านอารมณ์ของท่าน จำกัดกิจกรรมทางสังคมตามปกติของท่านที่มีกับครอบครัว หรือเพื่อนมากน้อยแค่ไหน

C19

- 1 ไม่เลย
- 2 น้อยมาก
- 3 พอสมควร
- 4 ค่อนข้างมาก
- 5 ไม่สามารถทำกิจกรรมทางสังคมต่างๆได้

C20 ในช่วง 4 สัปดาห์ที่ผ่านมา ปัญหาด้านอารมณ์ (*เช่นรู้สึกกังวล,ซึมเศร้า,หรือหงุดหงิด*) รบกวนท่านมากน้อยแค่ไหน

C20

- 1 ไม่เลย
- 2 เล็กน้อย
- 3 ปานกลาง
- 4 ค่อนข้างมาก
- 5 มากที่สุด

C21 ในช่วง 4 สัปดาห์ที่ผ่านมา ปัญหาส่วนตัวหรือปัญหาด้านอารมณ์ ทำให้ท่านไม่สามารถทำงาน เรียนหนังสือ หรือทำกิจกรรมประจำวันอื่นๆ ของท่านได้ตามปกติ มากน้อยเพียงใด

C21

- 1 ไม่เลย
- 2 น้อยมาก
- 3 พอสมควร
- 4 ค่อนข้างมาก
- 5 ไม่สามารถทำกิจกรรมประจำวันต่างๆได้

C22 ในช่วง 12 เดือนที่ผ่านมา ท่านได้รับบาดเจ็บร้ายแรงจนมีผลต่อการใช้ชีวิตประจำวัน และ/หรือ ถึงขั้นต้องได้รับการรักษาพยาบาล จำนวนกี่ครั้ง

C22

- 0 ไม่เคยได้รับบาดเจ็บ ⟶ **ข้ามไปตอบข้อ C28**
- 1 1 ครั้ง
- 2 2 ครั้ง
- 3 3 ครั้ง
- 4 4 ครั้งขึ้นไป

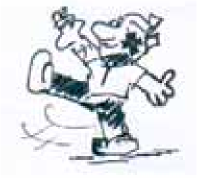

คำถามเกี่ยวกับการบาดเจ็บต่อไปนี้ (ข้อ C23-C27) โปรดให้คำตอบที่เกี่ยวข้องกับการบาดเจ็บ เฉพาะครั้งที่ท่านได้รับบาดเจ็บร้ายแรงที่สุดเพียงครั้งเดียว และอยู่ในช่วง 12 เดือนที่ผ่านมา

C23 การบาดเจ็บครั้งร้ายแรงนั้น เกิดขึ้นที่ไหน

C23

- 1 ที่พักอาศัย
- 2 ถนน/ตรอก/ซอย
- 3 สถานที่เล่นกีฬาหรือออกกำลังกาย
- 4 สถานที่ทำงาน (*ด้านเกษตรกรรมเช่นไร่ นา ฟาร์ม*)
- 5 สถานที่ทำงานอื่นๆ (*ที่ไม่เกี่ยวกับเกษตรกรรม*)
- 6 ที่อื่นๆ

C24 การบาดเจ็บครั้งร้ายแรงนั้น เกี่ยวข้องกับการจราจรใช่หรือไม่

C24

- 1 ใช่
- 2 ไม่ใช่ ⟶ **ข้ามไปตอบข้อ C26**

C25 หากเกี่ยวข้องกับการจราจร การบาดเจ็บครั้งนั้นเกิดขึ้นโดย

C25A ท่านมีบทบาทอย่างไร

C25A B 1 เป็นผู้ขับขี่ B 2 เป็นผู้โดยสาร B 3 เป็นผู้สัญจร → **ข้ามไปตอบข้อ C25C**

C25B ขณะเกิดเหตุ ยานพาหนะที่ท่านขับขี่/โดยสารคือ

C25B
B 1 รถจักรยาน B 2 รถจักรยานยนต์ B 3 รถโดยสาร/รถตู้/รถทัวร์
B 4 รถยนต์/รถปิ๊กอัพ B 5 อื่นๆ เช่นรถไฟ เรือ เครื่องบิน

C25C คู่กรณี ในการเกิดอุบัติเหตุ ครั้งร้ายแรงนั้น คือ

C25C
B 1 รถจักรยาน B 2 รถจักรยานยนต์ B 3 รถโดยสาร/รถตู้/รถทัวร์
B 4 รถยนต์/รถปิ๊กอัพ B 5 อื่นๆ เช่นรถไฟ เรือ เครื่องบิน
B 6 อื่น ๆ ที่ไม่ใช่ยานพาหนะเช่น ชนต้นไม้ สิ่งกีดขวาง ฯลฯ

C26 ถ้าไม่เกี่ยวข้องกับการจราจร การบาดเจ็บครั้งร้ายแรงนั้น มีสาเหตุหลักจากอะไร

C26
B 1 ถูกทำร้ายโดยไม่ใช้อาวุธ เช่น เตะ ต่อย B 6 ถูกยิง
B 2 พลัดตกหรือหกล้มเอง B 7 ของมีคมบาด/แทง
B 3 ถูกกระแทก/ของตกใส่หรืออื่นๆ B 8 ถูกไฟไหม้หรือความร้อน
B 4 จมน้ำ B 9 ถูกสารเคมีเป็นพิษ
B 5 ถูกแมลง/สัตว์ กัดหรือต่อย B 10 อื่นๆ

C27 การบาดเจ็บร้ายแรงครั้งนั้น เกิดขึ้นในลักษณะใด

C27
B 1 เป็นอุบัติเหตุ B 2 ไม่ใช่อุบัติเหตุ (มีบุคคลอื่นเกี่ยวข้อง)
B 3 ไม่ใช่อุบัติเหตุ (ไม่มีบุคคลอื่นเกี่ยวข้อง)

C28 ในช่วง 12 เดือนที่ผ่านมา ท่านได้ไปใช้บริการด้านสุขภาพสำหรับตัวท่านเอง จากสถานบริการเหล่านี้หรือไม่ *(เลือกได้มากกว่าหนึ่งคำตอบ)* **ค่าของตัวแปร (0 = ไม่เลือก; 1 = เลือก)**

C28_1 B สถานีอนามัย **C28_2** B โรงพยาบาลชุมชน
C28_3 B ศูนย์บริการสาธารณสุขของรัฐ **C28_4** B โรงพยาบาลจังหวัด/โรงพยาบาลของรัฐอื่นๆ
C28_5 B คลินิกเอกชน **C28_6** B โรงพยาบาลเอกชน
C28_7 B แพทย์แผนโบราณ *(แผนไทย/จี* **C28_8** B สถานบริการอื่นๆ
C28_9 B ไม่เคยใช้บริการต่าง ๆ ดังกล่าว → **ข้ามไปตอบ ข้อ C 30**

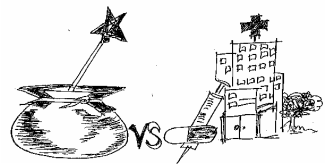

C29 ในช่วง12 เดือนที่ผ่านมา ท่านจ่ายค่าบริการด้านสุขภาพสำหรับตัวท่านเองโดยวิธีใด *(เลือกได้มากกว่าหนึ่งคำตอบ)*

C29_1 B ค่าประกันสังคม **C29_2** B ค่ารักษาพยาบาล*(ข้าราชการ/พนักงานของรัฐ/รัฐวิสาหกิจ)*
C29_3 B การประกันสุขภาพส่วนบุคค **C29_4** B โครงการ 30 บาท
C29_5 B จ่ายด้วยเงินของตนเอง **C29_6** B อื่นๆ **ค่าของตัวแปร (0 = ไม่เลือก; 1 = เลือก)**

C30 ในช่วง 12 เดือนที่ผ่านมา เคยมีหรือไม่ กรณีที่ท่านคิดว่า**ควรใช้**บริการรักษาพยาบาล **แต่ไม่ได้ใช้**

C30 B 1 มี B 2 ไม่มี ⟶ **ข้ามไปตอบข้อ C32**

C31 ถ้ามีกรณีที่ควรใช้บริการ แต่ไม่ได้ใช้เพราะเหตุใด *(เลือกได้มากกว่าหนึ่งคำตอบ)*

C31_1 B ค่าใช้จ่ายแพงเกินไป

C31_2 B ไม่สะดวกในการเดินทางไปรับบริการ

C31_3 B ต้องรอนานเกินไป

C31_4 B ไม่พอใจกับการบริการ

C31_5 B กลัวที่จะไป

C31_6 B ไม่ชอบหมอ/ พยาบาล/เจ้าหน้าที่สาธารณสุขอื่นๆ

C31_7 B ไม่สามารถลางานได้/งานยุ่ง

C31_8 B ไม่สามารถปลีกตัวจากภาระทางครอบครัว

C31_9 B อื่นๆ **ค่าของตัวแปร (0 = ไม่เลือก; 1 = เลือก)**

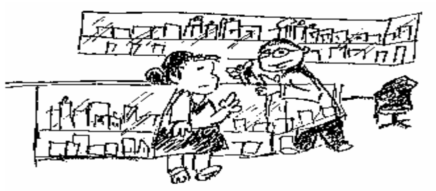

C32 ในช่วง 12 เดือนที่ผ่านมา ท่านเคยปฏิบัติดังข้อต่อไปนี้หรือไม่

(กาเครื่องหมายถูก ✓ ลงช่องที่ตรงตามที่ท่านทำจริง)

	เคย	ไม่เคย	
เคยใช้ยา(*กิน/ ฉีด/ ทา/ฯลฯ*) โดยแพทย์หรือเจ้าหน้าที่สาธารณสุขเป็นผู้สั่ง	1	2	C32N1
เคยซื้อยาที่ไม่ต้องใช้ใบสั่งยาจากแพทย์	1	2	C32N2
เคยใช้การรักษาแผนโบราณ /ยาสมุนไพร	1	2	C32N3

D เครือข่ายทางสังคม และคุณภาพชีวิต

D1 ท่านใช้เวลาทำกิจกรรมเหล่านี้บ่อยแค่ไหน *(กาเครื่องหมายถูก ✓ ลงช่องที่ตรงตามที่ท่านทำจริง)*

		ทุกวัน	ทุก/เกือบทุกสัปดาห์	1-2 ครั้งต่อเดือน	นานๆครั้งต่อปี	ไม่เคย
ใช้เวลากับบิดามารดาหรือเครือญาติ	D1N1	1	2	3	4	5
ใช้เวลาสังสรรค์กับเพื่อนบ้าน	D1N2	1	2	3	4	5
ใช้เวลาสังสรรค์กับเพื่อนร่วมงานหรือร่วมอาชีพ	D1N3	1	2	3	4	5
ใช้เวลาสังสรรค์กับเพื่อนอื่นๆ(เพื่อนเก่าฯลฯ)	D1N4	1	2	3	4	5
ใช้เวลาร่วมกับผู้อื่นในสถานที่ศักดิสิทธ์ เช่น โบสถ์ วัด	D1N5	1	2	3	4	5
ใช้เวลาร่วมกับผู้อื่นตามสถานที่พักผ่อน/งานอาสาสมัค	D1N6	1	2	3	4	5
ร่วมงานพรรคการเมือง/สมาคมการค้า/กลุ่มพิทักษ์สิ่งแ	D1N7	1	2	3	4	5

D2 โดยทั่วไป ท่านรู้สึกว่าสามารถวางใจผู้อื่นได้เพียงใด

D2 ☐1 สามารถวางใจได้เกือบทุกคน ☐2 ต้องระมัดระวังผู้อื่นตลอดเวลา

D3 ท่านคิดว่าบุคคล/องค์กรเหล่านี้ให้การสนับสนุนท่านมากน้อยแค่ไหน

(กาเครื่องหมายถูก ✓ ลงช่องที่ตรงตามความคิดเห็นของท่าน)

	น้อยมาก	ค่อนข้างน้อย	ค่อนข้างมาก	มากที่สุด	ไม่มีบุคคลดังกล่าว
ครอบครัวของท่าน **D3N1**	1	2	3	4	5
เพื่อนบ้าน/ผู้คนในท้องถิ่น **D3N2**	1	2	3	4	5
องค์กรปกครองส่วนท้องถิ่น **D3N3**	1	2	3	4	5
กลุ่มศาสนา **D3N4**	1	2	3	4	5
เพื่อน **D3N5**	1	2	3	4	5
นายจ้าง/เจ้านาย **D3N6**	1	2	3	4	5
ผู้อื่นในที่ทำงาน **D3N7**	1	2	3	4	5

D4 ท่านนับถือศาสนาอะไร

D4 ☐1 พุทธ ☐2 อิสลาม ☐3 คริสต์ ☐4 ฮินดู ☐5 อื่นๆ ☐6 ไม่มีศาสนา

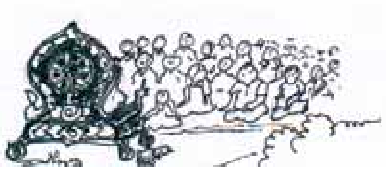

D5 โปรดให้ระดับคะแนนกับข้อความต่อไปนี้ ที่เกี่ยวข้องในชีวิตของท่าน

(กาเครื่องหมายถูก ✓ ลงช่องเพื่อให้ค่าคะแนนที่ตรงตามความคิดเห็นของท่าน)

	ไม่เลย ⟵									⟶ มากที่สุด
	0	1	2	3	4	5	6	7	8	9	10
เมื่อเผชิญกับปัญหา ศาสนามีส่วนสำคัญในการช่วยให้จิตใจท่านสงบลงมากน้อยในระดับใด **D5N1**											
พิธีกรรมต่างๆ (เช่น สะเดาะเคราะห์ สวดภาวนา ฯลฯ)มีความสำคัญต่อท่านมากน้อยในระดับใด **D5N2**											
ท่านคิดว่ากรรม มีความสำคัญมากน้อยในระดับใด **D5N3**											

D6 เมื่อคิดถึงชีวิตของท่านและสภาพแวดล้อมของท่าน ท่านมีความพอใจกับสิ่งต่อไปนี้ในระดับใด

(กาเครื่องหมายถูก ✓ ลงช่องเพื่อให้ค่าคะแนนที่ตรงตามความคิดเห็นของท่าน)

		ไม่พอใจอย่างยิ่ง ◄										► พอใจที่สุด
		0	1	2	3	4	5	6	7	8	9	10
มาตรฐานการดำรงชีวิต	D6N1											
สุขภาพของท่าน	D6N2											
ความสำเร็จในชีวิตของท่าน	D6N3											
ความสัมพันธ์กับผู้อื่น	D6N4											
ความรู้สึกปลอดภัย	D6N5											
ความรู้สึกเป็นส่วนหนึ่งของชุมชนที่ท่านอยู่	D6N6											
ความมั่นคงในอนาคต	D6N7											
สิ่งแวดล้อมที่ท่านอยู่อาศัย	D6N8											
ศาสนาหรือด้านจิตวิญญาณของท่าน	D6N9											
ชีวิตโดยรวมของท่าน	D6N10											

D7 ในช่วง <u>12 เดือนที่ผ่านมา</u> เคยมีหรือไม่ที่ท่านไม่สามารถมีสิ่งที่ต้องการมาก เนื่องจากมีเงินไม่พอ

D7 ☐ 1 เคย บ่อยครั้ง ☐ 2 เคย บางครั้ง ☐ 3 ไม่เคยเลย

E อาหารและกิจกรรมการเคลื่อนไหวร่างกาย

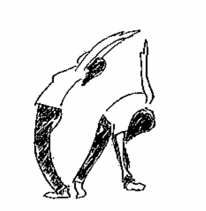

E1 โดยเฉลี่ยแล้ว ท่านรับประทานอาหารต่อไปนี้ บ่อยครั้งแค่ไหน

(กาเครื่องหมายถูก ✓ ลงช่องที่ตรงตามที่ท่านทำจริง)

		ไม่เคย/น้อยกว่าเดือนละครั้ง	1-3 ครั้ง/เดือน	1-2 ครั้ง/สัปดาห์	3- 6 ครั้ง/สัปดาห์	วันละครั้งหรือมากกว่า
อาหาร/ขนมหวานที่ประกอบด้วยกะทิ	E1N1	1	2	3	4	5
อาหารประเภททอด	E1N2	1	2	3	4	5
อาหารหมักดอง (ดิบ) เช่น แหนม ปลาร้า	E1N3	1	2	3	4	5
อาหารย่าง/รมควัน เช่น ไส้กรอก / ไก่ย่าง	E1N4	1	2	3	4	5
อาหารปรุงดิบ เช่น ลาบดิบ ก้อยฯลฯ	E1N5	1	2	3	4	5
ผักผลไม้ดอง	E1N6	1	2	3	4	5
อาหารกึ่งสำเร็จรูป เช่นบะหมี่	E1N7	1	2	3	4	5
อาหารกระป๋อง	E1N8	1	2	3	4	5
เครื่องดื่มประเภทน้ำอัดลม	E1N9	1	2	3	4	5
นม เช่น นมสด นมกล่อง นมผง ฯลฯ	E1N10	1	2	3	4	5
ผลิตภัณฑ์จากถั่วเหลืองเช่น น้ำเต้าหู้ เต้	E1N11	1	2	3	4	5
อาหารเสริมบำรุงสุขภาพเช่นวิตามิน เก	E1N12	1	2	3	4	5

E2 โดยปกติ ท่านรับประทานอาหารจากแหล่ง/สถานที่จำหน่ายอาหารต่อไปนี้บ่อยเพียงใด

(กาเครื่องหมายถูก ✓ ลงช่องที่ตรงตามที่ท่านทำจริง)

แหล่ง/สถานที่จำหน่ายอาหาร	ไม่เคยหรือน้อยกว่า 1 ครั้ง/เดือน	1-2 ครั้ง/เดือน	1 ครั้ง/สัปดาห์ หรือมากกว่า	ทุกวัน หรือ เกือบทุกวัน
ภัตตาคาร (ที่จ่ายมากกว่า 200 บาท/คน/ครั้ง) **E2N1**	1	2	3	4
ร้านอาหารทั่วไป (ที่จ่ายน้อยกว่า 100 บาท/คนครั้ **E2N2**	1	2	3	4
ร้านอาหารฟาสต์ฟู้ดแบบตะวันตก เช่น เบอร์เกอร์ **E2N3**	1	2	3	4
ร้านอาหารในที่ทำงาน **E2N4**	1	2	3	4
หาบเร่/แผงลอย/ตลาด **E2N5**	1	2	3	4
บริการส่งถึงบ้าน เช่น พิซซ่า **E2N6**	1	2	3	4
อาหารที่ทำที่บ้าน **E2N7**	1	2	3	4

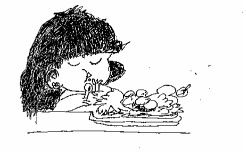

E3 ข้อความต่อไปนี้ มีอิทธิพลมากน้อยเพียงใด ต่อการเลือกรับประทานอาหารของท่าน

(กาเครื่องหมายถูก ✓ ลงช่องที่ตรงตามความคิดเห็นของท่าน)

	สำคัญมาก	สำคัญ	สำคัญน้อย	ไม่สำคัญ
ราคา- อาหารที่สามารถจ่ายเพื่อซื้อหาได้ **E3N1**	1	2	3	4
สุขภาพ- อาหารที่มีประโยชน์ต่อสุขภาพ **E3N2**	1	2	3	4
รสชาติ- อาหารที่อร่อย ถูกปาก **E3N3**	1	2	3	4
ความสะดวก-อาหารที่สะดวกในการบริโภค **E3N4**	1	2	3	4
อาหารที่ปรุงง่ายๆ ไม่ต้องอาศัยเครื่องครัวยุ่งยาก **E3N5**	1	2	3	4
อาหารที่ง่ายในการเคลื่อนย้าย หอบหิ้ว **E3N6**	1	2	3	4
อาหารตามวัฒนธรรมแบบดั้งเดิม **E3N7**	1	2	3	4
ความเคยชิน- อาหารที่ชอบรับประทาน **E3N8**	1	2	3	4
อาหารตามข้อกำหนดทางศาสนา **E3N9**	1	2	3	4
ความปลอดภัยของอาหาร **E3N10**	1	2	3	4

**E4** ท่านรับประทานผักจำนวนกี่หน่วยต่อวัน *(ผัก 1 หน่วย = ผักปรุงสุกครึ่ง ถ้วยหรือ ผักดิบ 1 ถ้วย)*	**E4**	N N หน่วย
**E5** ท่านรับประทานผลไม้จำนวนกี่หน่วยต่อวัน *(ผลไม้ 1 หน่วย = ผลไม้หั่นเป็นชิ้น ประมาณ 6 คำ) เช่น กล้วย 1 ผล นับเป็น 1 หน่วย หรือผลไม้ หั่นเป็นสี่เหลี่ยมลูกเต๋าแล้วตวงได้ 1 ถ้วย*	**E5**	N N หน่วย

E6 โดยปกติ ท่านใช้เวลาเพื่อออกกำลังกายดังต่อไปนี้ ประมาณกี่ครั้งต่อสัปดาห์ (*ถ้า**ไม่ได้**ออกกำลังกายประเภทนั้นๆ โปรดใส่เลข 0 ในช่อง* N N *ที่ให้ไว้ ดังนี้* N N)

		**จำนวนครั้ง/สัปดาห์**
ออกกำลังกายที่ใช้แรงมาก (หัวใจเต้นแรงและเร็ว) เป็นเวลา**นานกว่า 20 นาที**ต่อครั้ง *เช่น การยกน้ำหนัก การขุดดิน แอโรบิค ขี่จักรยานความเร็วสูง วิ่ง เตะตะกร้อ ฯลฯ*	**E6A**	N N
ออกกำลังกายที่ใช้แรงระดับปานกลาง (ไม่เหนื่อยมากแต่หายใจแรงกว่าปกติ) เป็นเวลา**นานกว่า 20 นาที**ต่อครั้ง *เช่น ยกน้ำหนักที่เบา ขี่จักรยานโดยความเร็วคงที่ ฯลฯ*	**E6B**	N N
ออกกำลังกายเบา ๆ (ใช้แรงเล็กน้อย) เป็นเวลา**นานกว่า 20 นาที**ต่อครั้ง เช่น โยคะ ไทเก๊ก โบ	**E6C**	N N
เดินอย่างต่อเนื่อง (ไม่หยุดพัก) **<u>อย่างน้อย 10 นาที</u>** *เช่น เดินในที่ทำงาน ที่บ้าน หรือเดินออกกำลัง*	**E6D**	N N

E7 ท่านทำงานบ้าน เช่นทำความสะอาด หรือทำสวน บ่อยครั้งเพียงใด

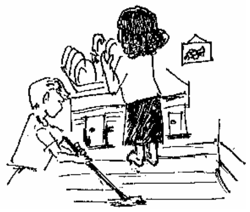

E7
- b 1 น้อยมากหรือไม่เคยเลย
- b 2 1–3 ครั้ง / เดือน
- b 3 1-2 ครั้ง / สัปดาห์
- b 4 3 – 4 ครั้ง / สัปดาห์
- b 5 เกือบทุกวัน

E8 โดยปกติ ท่านใช้เวลาในการทำสิ่งเหล่านี้ โดยรวมประมาณวันละกี่ชั่วโมง

**กิจกรรม**	**ระยะเวลา**	
นอน *(หากนอนกลางวันเป็นประจำให้รวมด้วย)*	N N ชั่วโมง/วัน	**E8A**
ดูโทรทัศน์ <u>และ/หรือ</u> **เล่น**เกมคอมพิวเตอร์	N N ชั่วโมง/วัน	**E8B**
นั่งในทุกๆกรณี (*เช่นนั่งอ่านหนังสือ นั่งพักผ่อน นั่งทำงาน นั่งคิด ฯลฯ*)	N N ชั่วโมง/วัน	**E8C**

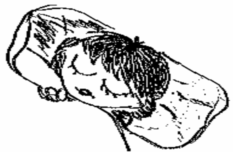

F บุหรี่ แอลกอฮอล์ และการสัญจร

F1 ท่านเคยสูบบุหรี่หรือไม่

F1 b 1 เคยสูบบุหรี่ b 2 ไม่เคยสูบบุหรี่ ⟶ **ข้ามไปตอบข้อ F5**

F2 ท่าน **เริ่ม** สูบบุหรี่ครั้งแรกเมื่ออายุ **F2** N N ปี

F3 หากท่าน **เลิก** สูบแล้ว ท่านเลิกสูบเมื่อ **F3B** N N ปี b ปัจจุบันยังสูบบุหรี่อยู่ **F3A**

(0 = ไม่เลือก; 1 = เลือก)

F4 ท่านสูบหรือเคยสูบ โดยเฉลี่ยบุหรี่วันละ **F4** N N มวน

F5 โดยปกติ ท่านมักได้รับสัมผัสกับควันบุหรี่จากการที่ผู้อื่นสูบบุหรี่ ในสถานที่ใดบ้าง (*เลือกได้มากกว่าหนึ่งคำตอบ*)

F5_1 b ที่บ้าน **F5_2** b ในสถานที่พักผ่อน

F5_3 b ที่ทำงาน **F5_4** b สถานขนส่งมวลชน เช่น สถานีรถไฟ รถทัวร์

F5_5 b ที่อื่น ๆ **F5_6** b ไม่เคย ค่าของตัวแปร **(0 = ไม่เลือก; 1 = เลือก)**

F6 ท่านเคยดื่ม เครื่องดื่มแอลกอฮอล์หรือไม่

F6
b 1 ดื่มเป็นครั้งคราวเมื่อเข้าสังคม b 3 ไม่เคยดื่ม ⟶ **ข้ามไปตอบข้อ F11**
b 2 ดื่มเป็นประจำ b 4 เคยดื่มแต่ปัจจุบัน **เลิก** ดื่มแล้ว

F7 ท่านเริ่มดื่มเครื่องดื่มแอลกอฮอล์เมื่ออายุ **F7** N N ปี

F8 หากท่าน **เลิก** ดื่มเครื่องดื่มแอลกอฮอล์แล้ว ท่านเลิกดื่มเมื่ออายุ **F8B** N N ปี **F8A** b ปัจจุบันยังดื่มอยู่

F9 โดยทั่วไป ท่านดื่ม/เคยดื่มเครื่องดื่มแอลกอฮอล์ประมาณกี่แก้ว (ในการดื่มแต่ละครั้ง)

F9
b 1 น้อยกว่า 2 แก้ว b 2 2-3 แก้ว
b 3 4-5 แก้ว b 4 6 แก้วหรือมากกว่า

F10 ในช่วง 12 เดือนที่ผ่านมา ท่านเคยขับขี่ยานพาหนะหลังจากดื่มเครื่องดื่มแอลกอฮอล์ตั้งแต่ 3 แก้วหรือมากกว่า หรือไม่

F10 b 1 เคย b 2 ไม่เคย b 3 ปกติไม่ได้ขับขี่ยานพาหนะ

F 11 ท่านหรือบุคคลในบ้านท่านเป็นเจ้าของยานพาหนะใดบ้าง (*เลือกได้มากกว่าหนึ่งคำตอบ*)

F11_1 b จักรยาน **F11_2** b จักรยาน **F11_3** b รถยนต์/ปิ๊กอัพ/รถตู้

F11_4 b รถบรรทุก **F11_5** b เรือ **F11_6** b ไม่มียานพาหนะ

ค่าของตัวแปร **(0 = ไม่เลือก; 1 = เลือก)**

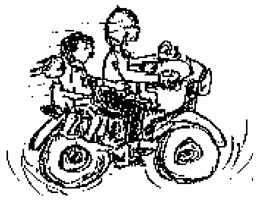

F12 ท่านใช้เข็มขัดนิรภัยขณะขับขี่หรือนั่งในรถยนต์บ่อยแค่ไหน

(*โปรดตอบทั้งสำหรับที่นั่งด้านหน้าและด้านหลัง โดยกาเครื่องหมายถูก ✓ ลงช่องที่ตรงตามที่ทำจริง*)

การใช้เข็มขัดนิรภัย		ใช้เข็มขัดนิรภัยเป็นประจำ	ใช้เข็มขัดนิรภัยเป็นบางครั้ง	ไม่เคยใช้เข็มขัดนิรภัย	รถยนต์ไม่มีเข็มขัดนิรภัย
ที่นั่งด้านหน้า	**F12N1**	**1**	**2**	**3**	**4**
ที่นั่งด้านหลัง	**F12N2**	**1**	**2**	**3**	**4**

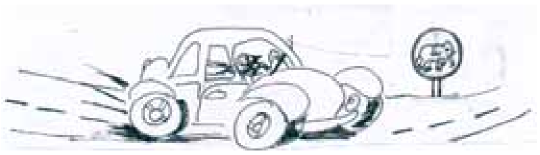

F13 ท่านสวมหมวกนิรภัยในขณะขับขี่หรือนั่งรถจักรยานยนต์บ่อยแค่ไหน

F13

b 1 ประจำ b 2 บางครั้ง

b 3 น้อยครั้งหรือไม่เคย b 4 ไม่ใช้รถจักรยานยนต์

F14 ท่านนั่งหลังรถกระบะหรือรถบรรทุก/ปิ๊กอัพที่ไม่มีหลังคาบ่อยครั้งหรือไม่

F14 B 1 เป็นประจำ b 2 เป็นบางครั้ง b 3 น้อยมาก /ไม่เคย

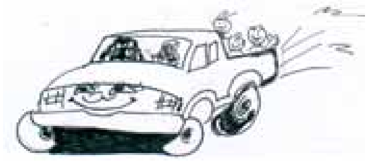

G ครอบครัวของท่าน

G1 คำถามต่อไปนี้เกี่ยวกับมารดาและ/หรือบิดาของท่าน

มารดา	บิดา
ระดับการศึกษาสูงสุดของมารดาของท่าน (**G1M1**)<br>B 1 ไม่ได้เรียนในโรงเรียน<br>b 2 ระดับประถมศึกษา<br>b 3 ระดับมัธยมศึกษา<br>b 4 ระดับปริญญา<br>b 5 ไม่ทราบ	ระดับการศึกษาสูงสุดของบิดาของท่าน (**G1F1**)<br>B 1 ไม่ได้เรียนในโรงเรียน<br>b 2 ระดับประถมศึกษา<br>b 3 ระดับมัธยมศึกษา<br>b 4 ระดับปริญญา<br>b 5 ไม่ทราบ
มารดาของท่านยังมีชีวิตอยู่หรือไม่ (**G1M2**) **G1MAGE**<br>b 1 ถึงแก่กรรมแล้ว เมื่ออายุ N N ปี<br>B 2 ยังมีชีวิต ขณะนี้อายุ N N ปี	บิดาของท่านยังมีชีวิตอยู่หรือไม่ (**G1F2**) **G1FAGE**<br>b 1 ถึงแก่กรรมแล้ว เมื่ออายุ N N ปี<br>b 2 ยังมีชีวิต ขณะนี้อายุ N N ปี

หากทั้งมารดาและบิดามีชีวิตอยู่ทั้งคู่ ⟶ **ข้ามไปตอบข้อ G 3**

G2 หากมารดาหรือบิดาของท่านถึงแก่กรรมแล้ว ท่านถึงแก่กรรมเพราะสาเหตุใด *(เลือกได้มากกว่าหนึ่งคำตอบ)*

โปรดกาเครื่องหมายถูกลงในช่องที่เหมาะสม ☑ และโปรด**เว้นว่าง** สำหรับช่องที่ไม่เกี่ยวข้อง

	มารดา	บิดา		มารดา	บิดา
โรคหัวใจ **G2M_1**	b	b **G2F_1**	เบาหวาน **G2M_8**	b	b **G2F_8**
ความดันโลหิตสู **G2M_2**	b	b **G2F_2**	โรคไต **G2M_9**	b	b **G2F_9**
โรคหลอดเลือดใ **G2M_3**	b	b **G2F_3**	บาดเจ็บ **G2M_10**	b	b **G2F_10**
มะเร็ง **G2M_4**	b	b **G2F_4**	เสียเนื่องจากคล **G2M_11**	b	
วัณโรค **G2M_5**	b	b **G2F_5**	สูงอายุ **G2M_12**	b	b **G2F_11**
ปอดบวม **G2M_6**	b	b **G2F_6**	อื่นๆ **G2M_13**	b	b **G2F_12**
โรคติดเชื้ออื่นๆ **G2M_7**	b	b **G2F_7**	ไม่ทราบ **G2M_14**	b	b **G2F_13**

ค่าของตัวแปร **(0 = ไม่เลือก; 1 = เลือก)**

G3 ตัวท่านเองมีบุตรหรือไม่

G3 B1 มี b2 ไม่มี ──► **ข้ามไปตอบข้อ G18**

คำถามต่อไปนี้เกี่ยวกับบุตรของท่าน

G4 ปัจจุบันท่านมีบุตรสาว **G4A** N N คน

และบุตรชาย **G4B** N N คน

G5 ท่านต้องการมีบุตร เพิ่มอีกหรือไม่

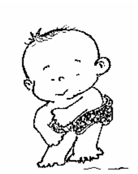

G5
B1 ต้องการ
b2 ไม่ต้องการ

G6 ท่าน**เคย**มีบุตรที่เกิดมาแล้ว และภายหลังเสียชีวิต หรือไม่

G6
B1 มี
b2 ไม่มี

G7 เมื่อมีบุตรคนแรก ตัวท่านเองในขณะนั้นอายุ เท่าไร

G7 อายุ N N ปี

G8 บุตรคนเล็กของท่านเป็นบุตรสาวหรือบุตรชาย

G8
B1 บุตรสาว
b2 บุตรชาย

G9 บุตรคนเล็กของท่านขณะนี้ อายุเท่าไร

G9A อายุ N N ปี **G9B** N N เดือน

(เช่น 3 ปี 11 เดือน) บุตรคนเดียวนับเป็นคนเล็กด้วย

G10 บุตรคนเล็กของท่านมีน้ำหนักและส่วนสูงเท่าไร

G10B น้ำหนัก N N . N กิโลกรัม **G1A** b ไม่ทราบ

(เช่น 10.5 กิโลกรัม) **(0 = ไม่เลือก; 1 = เลือก)**

G10D ส่วนสูง N N N เซนติเมตร **G10C** b ไม่ทราบ

G11 บุตรคนเล็กของท่าน มีน้ำหนักแรกเกิดเท่าไร

น้ำหนักแรกเกิด N N N N **G11B** กรัม

B ไม่ทราบ **G11A**

(0 = ไม่เลือก; 1 = เลือก)

G12 บุตรคนเล็กของท่าน ได้ดื่มนมมารดาหรือไม่

B1 ได้ดื่ม **G12**
b2 ไม่ได้ดื่ม ──► **ข้ามไปตอบข้อ G14**

G 13 บุตรคนเล็กของท่านดื่มนมมารดานานกี่เดือน

ได้ดื่มนาน N N เดือน **G13**

G14 ท่านมีบุตรที่แพทย์วินิจฉัยว่าเป็นหอบหืด หรือไม่

B1 มี **G14**
b2 ไม่มี ──► **ข้ามไปตอบข้อ G16**

G15 ถ้ามี บุตรลำดับที่เท่าไรที่เป็นหอบหืด

(เลือกได้มากกว่าหนึ่งคำตอบ)

B **G15_1** b คนที่ 3 **G15_3**
b **G15_2** b คนที่ 4 **G15_4**

ค่าของตัวแปร (0 = ไม่เลือก; 1 = เลือก)

G16 ท่านมีบุตรที่เป็นภูมิแพ้หรือไม่ *(เช่น ไอ จาม น้ำมูกไหล **ที่ไม่ใช่** เกิดจากการเป็นหวัด)*

B1 มี **G16**
b2 ไม่มี ──► **ข้ามไปตอบข้อ G18**

G17 ถ้ามี บุตรลำดับที่เท่าไรที่เป็นภูมิแพ้

(เลือกได้มากกว่าหนึ่งคำตอบ)

B **G17_1** b คนที่ 3 **G17_3**
b **G17_2** b คนที่ 4 **G17_4**

ค่าของตัวแปร (0 = ไม่เลือก; 1 = เลือก)

คำถามสุดท้ายเกี่ยวกับสัตว์เลี้ยงของท่าน

G18 บ้านท่านมีสัตว์เลี้ยงเหล่านี้หรือไม่

(เลือกได้มากกว่าหนึ่งคำตอบ)

B **G18_1** b แมว **G18_2**
b **G18_3** b อื่น ๆ **G18_4**
b ไม่มีสัตว์เลี้ยง **G18_5**

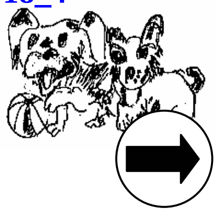

ค่าของตัวแปร (0 = ไม่เลือก; 1 = เลือก)

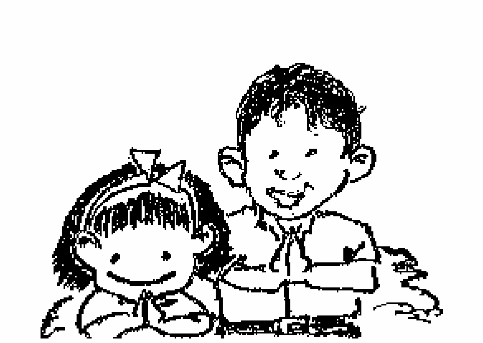

โครงการวิจัยสุขภาพ มหาวิทยาลัยสุโขทัยธรรมาธิราช ขอขอบคุณทุกท่านสำหรับความร่วมมือครั้งนี้

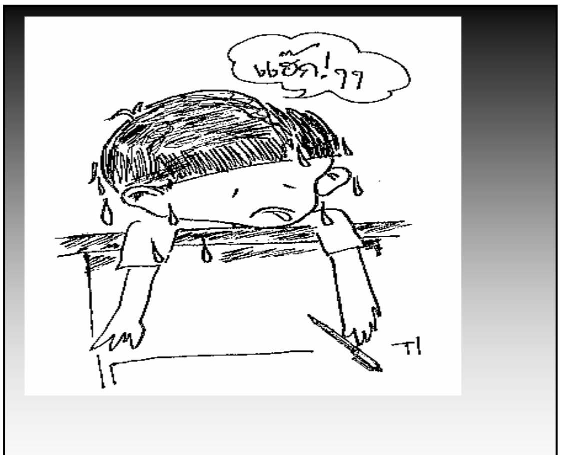

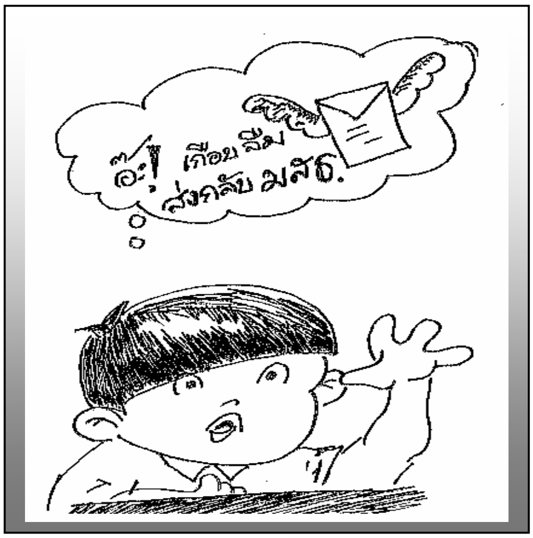

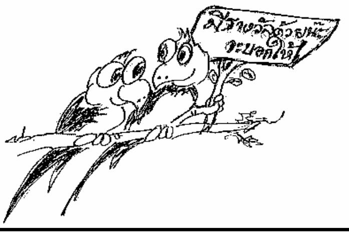

Supplement: Additional file 2 — Baseline 2005 Questionnaire (Thai). The actual Thai language questionnaire sent to all 200,000 students enrolled at Sukhothai Thammathirat Open University in 2005 [file 1756-0500-4-166-S2.PDF]
