# Supplementary material for: Methods used for successful follow-up in a large scale national cohort study in Thailand
Source: BMC Res Notes. 2011 May 27;4:166. doi: 10.1186/1756-0500-4-166 (PMC3123220; doi:10.1186/1756-0500-4-166)
Supplement: Additional file 4 — 2007 short follow-up questionnaire (English). An English translation of the short 2 year follow-up questionnaire sent to a random 10% sample of respondents to the 2005 baseline questionnaire [file 1756-0500-4-166-S4.PDF]

**First name**…………………...... …**Family Name**………………..<br><br>**Contact phone number**………	**ID** ☐☐☐☐☐

A1. What is your current weight ☐☐☐ kg. **A2.** Height ☐☐☐ cm. (please put in numbers)

Please answer the following questions by putting a tick mark ✓ <u>in 1 box</u> ☐ that fits best

A3. Do you think you are

☐ underweight ☐ normal ☐ overweight

B1. <u>In the last 12 months</u> have you had any injury that was serious enough to interfere with daily activities and/or required medical treatment? ☐ Yes ☐ No ➔ **please skip to question C below**

Please answer the following questions for the most serious injury <u>in the last 12 months</u>

B2. Did you see a doctor ☐ Yes ☐ No

B3. Were you admitted to hospital? ☐ Yes ☐ No

B4. Where were you when you were injured?

☐ home ☐ agricultural workplace

☐ road ☐ non-agricultural workplace

☐ sports facility ☐ Other

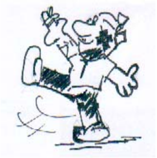

If the injury in B4 is not involved with traffic ➔ please skip to question C below

B5. If a traffic injury, what was your role?

☐ Driver ☐ Passenger ☐ Pedestrian ➔ **please skip to question C below**

B6. What type of vehicle were you driving or a passenger in?

☐ Bicycle ☐ Motorcycle ☐ Bus/van/coach

☐ Car/pickup ☐ Others e.g. train, boat, airplane

**C.** In the past 4 week, how often …	**All the time**	**Most of the time**	**Some of the time**	**A little of the time**	**None**
**C1.** did you feel nervous?	☐	☐	☐	☐	☐
**C2.** did you feel restless or fidgety?	☐	☐	☐	☐	☐
**C3.** did you fee that everything was an effort	☐	☐	☐	☐	☐
**C4.** did you feel sad?	☐	☐	☐	☐	☐
**C5.** did you feel happy?	☐	☐	☐	☐	☐

D1. Do you have a current email address? ☐ Yes ☐ No

D2. If yes, please write it clearly down here………………………………………………………..…………………

D3. How often do you get on the internet? ☐ Almost daily ☐ At least once a week

☐ Less than once a week ☐ Never

D4. In the future, if the TCS post questionnaire on the internet would you be willing to answer online/ by email?

☐ Yes ☐ No

E. Have you ever been diagnosed by a doctor of the following diseases? (you can tick more than 1 box)

Please put a check mark ✓ in boxes that apply and please put <u>the age, hospital name/ institute/ clinic etc. when you were first diagnosed.</u>

**Diseases**	**Yes (please check ✓)**	**Age when first diagnosed**	**Hospital/institute/clinic giving the dianosis**
Ex. Measles (diagnosed by a doctor at age 14)	☑	1 4	**Krabi Hospital**
**E1.** Diabetes (needing insulin)	❑		
**E2.** Diabetes (do not need insulin)	❑		
**E3.** High cholesterol/high blood lipids	❑		
**E4.** High blood pressure	❑		
**E5.** Ischemic (coronary) heart disease	❑		
**E6.** Cerebrovascular disease (Stroke)	❑		
**E7.** Liver cancer	❑		
**E8.** Lung cancer	❑		
**E9a.** Cancer of the digestive system	❑		
**E9b.** Colon cancer	❑		
**E10.** Breast cancer	❑		
**E11.** Other cancers	❑		
**E12.** Goiter/Thyroid abnormality	❑		
**E13.** Epilepsy	❑		
**E14.** Liver disease (not cancer)	❑		
**E15.** Kidney disease	❑		
**E16.** Depression/anxiety	❑		
**E17.** Arthritis	❑		
**E18.** Pneumonia	❑		
**E19.** Chronic bronchitis/lung disease	❑		
**E20.** Asthma	❑		
**E21.** Malaria	❑		
**E22.**Dengue fever	❑		
**E23.** Tuberculosis	❑		
**E24.** Other chronic infection	❑		
