# Supplementary material for: Methods used for successful follow-up in a large scale national cohort study in Thailand
Source: BMC Res Notes. 2011 May 27;4:166. doi: 10.1186/1756-0500-4-166 (PMC3123220; doi:10.1186/1756-0500-4-166)
Supplement: Additional file 5 — 2007 short follow-up questionnaire (Thai). The Thai language short 2 year follow-up questionnaire sent to a random 10% sample of respondents to the 2005 baseline questionnaire [file 1756-0500-4-166-S5.DOC]

tcsId ………………………………………………….. รหัสนักศึกษา nnnnnnn

ชื่อ _________________________ นามสกุล _________________________

**A1.** ปัจจุบันท่านมีน้ำหนัก   nnn  ก.ก.  **A2.** ความสูง nnn ซ.ม.

**A3**. ท่านคิดว่าตนเองมีรูปร่างเป็นอย่างไร (โปรดเลือกข้อที่เห็นเหมาะสมที่สุดเพียง 1 ข้อ)

B ผอม B หนักปกติ B อ้วน

B1. ในช่วง **12 เดือนที่ผ่านมา** ท่านเคยได้รับบาดเจ็บที่ส่งผลกระทบต่อการใช้ชีวิตประจำวัน และ/หรือ ต้องได้รับการรักษาพยาบาลบ้างหรือไม่

**B** ไม่เคย     (ข้ามไปตอบข้อ C)

**B** เคย    B2. ถ้าเคย บาดเจ็บกี่ครั้ง nn ครั้ง

สำหรับการบาดเจ็บครั้งที่ร้ายแรงที่สุดตามคำตอบข้อ B2

ท่านได้รับการตรวจจากแพทย์หรือไม่ **❑** ได้  **❑** ไม่ได้

ท่านได้เข้าพักรักษาตัวในโรงพยาบาลหรือไม่  **❑** ได้  **❑** ไม่ได้

**B3.**  การบาดเจ็บครั้งที่ร้ายแรงที่สุดตามคำตอบข้อ B2 นั้นเกิดขึ้นที่ไหน (โปรดเลือกข้อที่เหมาะสมที่สุด 1 ข้อ)

**B** บ้าน  **B** ถนนหนทาง **B** สถานที่ออกกำลังกาย

**B** ที่ทำงาน (ทางการเกษตร) **B** ที่ทำงาน(ไม่ใช่การเกษตร) **B** ที่อื่น

 B4**.** หากเป็นการบาดเจ็บเกี่ยวกับการจราจรในถนนหนทาง ท่านมีบทบาทอย่างไร (เลือกข้อที่เหมาะสมที่สุด 1 ข้อ) **B** ไม่เกี่ยวกับการจราจร(ข้ามไปตอบข้อ C)

**B** เป็นผู้ขับขี่  **B** เป็นผู้โดยสาร **B** เป็นผู้สัญจร(ข้ามไปตอบข้อ C)

B5. ขณะเกิดเหตุ ยานพาหนะที่ท่านขับขี่/โดยสารคือ

**B** รถจักรยาน **B** รถจักรยานยนต์ **B** รถโดยสาร/รถตู้/รถทัวร์

**B** รถยนต์/รถปิ๊กอัพ **B** อื่นๆ เช่นรถไฟ เรือ เครื่องบิน

**C.**ช่วง 4 สัปดาห์ที่ผ่านมา ท่านมีความรู้สึกดังต่อไปนี้บ่อยมากแค่ไหน (กรุณากาเครื่องหมายถูก ✓

**ในช่องที่เหมาะสมที่สุด)**

|  | ตลอด  เวลา | เกือบ  ตลอดเวลา | บางเวลา | น้อยครั้งมาก | ไม่เคยเลย |
| --- | --- | --- | --- | --- | --- |
| **C1.** รู้สึกกังวล | **B** | **B** | **B** | **B** | **B** |
| **C2.**    รู้สึกกระวนกระวาย หรือหงุดหงิด | **B** | **B** | **B** | **B** | **B** |
| **C3.**    รู้สึกต้องใช้ความพยายามในการทำทุกสิ่งทุกอย่าง | **B** | **B** | **B** | **B** | **B** |
| **C4.**    รู้สึกเศร้า | **B** | **B** | **B** | **B** | **B** |
| **C5.** รู้สึกมีความสุข | **B** | **B** | **B** | **B** | **B** |

**ข้อมูลการใช้เทคโนโลยีอินเตอร์เน็ต**

**D1**. ท่านมีที่อยู่อีเมลล์ที่เป็นปัจจุบันหรือไม่ **B** มี **B** ไม่มี

**D2**.   หากมี อีเมลล์ของท่านคือ (โปรดเขียนตัวบรรจง) : ....................................................................

ท่านมีอินเตอร์เน็ตใช้หรือไม่  **D3**.  ที่บ้าน **B** มี **B** ไม่มี

**D4.**  ที่ทำงาน **B** มี **B** ไม่มี

**D5,**  ที่อื่นๆ **B** มี **B** ไม่มี

**D6.** ในอนาคตหากโครงการฯจัดทำแบบสอบถามทางอินเตอร์เน็ตหรืออีเมลล์ ท่านยินดีใช้บริการหรือไม่ **B** ใช้ **B** ไม่ใช้

E. ท่านเคยได้รับการ**วินิจฉัยโดยแพทย์**ว่าป่วยเป็นโรคใดต่อไปนี้

**B** ไม่เคยเข้ารับการตรวจวินิจฉัย

**B** ไม่เป็นโรคใดๆ

**B** เคยได้รับการวินิจฉัยว่าเป็นป่วยด้วยโรค...... (*เลือกได้มากกว่าหนึ่งคำตอบ* โดยใส่เครื่องหมาย ✓ ในช่องเป็นและโปรดระบุอายุของท่านเมื่อได้รับการวินิจฉัยว่าเป็นโรคนั้นๆและชื่อ รพ./สถาบัน/คลีนิค ฯลฯ ที่ให้การวินิจฉัยด้วย)

|  | โรค | เป็น(โปรดใส่เครื่องหมาย ✓) | อายุที่ตรวจพบ (ปี) | ชื่อ รพ/สถาบัน/คลีนิคที่ให้การวินิจฉัยโรค |
| --- | --- | --- | --- | --- |
| 1 | เบาหวาน (ต้องใช้อินซูลิน) |  |  |  |
| 2 | เบาหวาน (ไม่ต้องใช้อินซูลิน) |  |  |  |
| 3 | โคเลสเตอรอลสูงหรือไขมันในเลือดสูง |  |  |  |
| 4 | ความดันโลหิตสูง |  |  |  |
| 5 | โรคหัวใจขาดเลือด |  |  |  |
| 6 | โรคหลอดเลือดในสมอง (Stroke) |  |  |  |
| 7 | มะเร็งตับ |  |  |  |
| 8 | มะเร็งปอด |  |  |  |
| 9 | มะเร็งของระบบทางเดินอาหาร |  |  |  |
| 10 | มะเร็งเต้านม |  |  |  |
| 11 | มะเร็งอวัยวะอื่นๆ |  |  |  |
| 12 | คอพอก/ต่อมไทรอยด์ผิดปกติ |  |  |  |
| 13 | ลมบ้าหมู |  |  |  |
| 14 | โรคเกี่ยวกับตับ (ไม่ใช่มะเร็ง) |  |  |  |
| 15 | โรคเกี่ยวกับไต |  |  |  |
| 16 | ซึมเศร้า/วิตกกังวล |  |  |  |
| 17 | ข้ออักเสบ |  |  |  |
| 18 | ปอดบวม( นิวมอเนีย) |  |  |  |
| 19 | หลอดลมอักเสบเรื้อรัง/โรคปอดอื่นๆ |  |  |  |
| 20 | หอบหืด |  |  |  |
| 21 | มาเลเรีย (ไข้จับสั่น) |  |  |  |
| 22 | ไข้เลือดออก |  |  |  |
| 23 | วัณโรค |  |  |  |
| 24 | โรคติดเชื้อเรื้อรังอื่นๆ |  |  |  |
| 25 | เป็นโรคอื่นที่นอกเหนือจากที่กล่าวข้างต้น |  |  |  |
