# Supplementary material for: Methods used for successful follow-up in a large scale national cohort study in Thailand
Source: BMC Res Notes. 2011 May 27;4:166. doi: 10.1186/1756-0500-4-166 (PMC3123220; doi:10.1186/1756-0500-4-166)
Supplement: Additional file 6 — 2009 4-year follow up questionnaire (English). An English translation of the 4-year follow-up questionnaire mailed out to all cohort members (n = 85,217) in 2009. [file 1756-0500-4-166-S6.PDF]

The Thai Health-Risk Transition: A National Cohort Study

Name: ____

St. No.: ____ Moo Ban:______ Soi:______ Rd. _____

Tambol/Kwang: ________________________________

District/Khet _________ Province:________________

Postcode:_______________________________________

0 1 5 7 7

MEMBER CODE TCSID | 0 | 1 | 5 | 7 | 7 |

Dear STOU cohort member,

Thank you for participating in the Thai Health-Risk Transition study. The information you provided 3 years ago shows that there are many factors affecting our health as we reported back to you last year when we posted the 'Love Your Health' booklet.

We are now sending you a *first follow-up questionnaire* which will explore the changes you have experienced in the last 3 years. This new information will enrich our understanding of health and well-being in Thailand. With these insights we can help develop national responses to help Thais live healthy and fulfilling lives.

It is a privilege to work with you on this health project. We hope you find time to complete the questions attached and return by post in the stamped envelope provided.

We understand that your participation is voluntary and that you may withdraw from the study at any time. We certify that all your information will remain confidential. Your name and other details will never be released, and will only be used to make further contact with you. For any analyses and reports all identifying information will be removed from the data.

If you are the person to whom this document was addressed above, and you are willing to continue participating, please sign below.

Your Signature .... Date .... / ...... / ......

Mr/Miss/Mrs ....

If you changed your address or contact details please fill in the information on the next page and return it with the questionnaire in the envelope provided.

For any assistance regarding this study please call our help line toll free 02-5047780 (working hours). Thank-you to all our members.

Associate Professor Sam-ang Seubsman
Principal Investigator, Thai Health-Risk Transition Study
Sukhothai Thammathirat Open University, Nonthaburi

This page will be separated and treated as confidential

It is important for our study that we can maintain contact with you. If your name, address or other details have changed from those shown on the front of the envelope, please place a cross in one of the following boxes. Please use a blue or black pen

☐ **Have not changed any personal information** → **Please go to the instructions at the top of the next page**

☐ **There has been a change to my personal information as follows**

☐ Name ☐ Address ☐ Telephone number

Please give details below

First Name.. Family Name…..

Address: No.............. Moo Ban.............….……. Soi..........….....……... Road……………….……………

Tambol/Kwang................................. District/Khet............................. Province……………………….

Postcode ☐☐☐☐☐

Home Tel......................................…Office Tel…………..………….. Mobile…………….…………….

Email..…………………………………………………………………….

Other contact person (if we cannot contact you)

First Name.. Family Name…..

Address: No.............. Moo Ban.............….……. Soi..........….....……... Road……………….……………

Tambol/Kwang................................. District/Khet............................. Province……………………….

Postcode ☐☐☐☐☐

Home Tel......................................…Office Tel…………..………….. Mobile…………….…………….

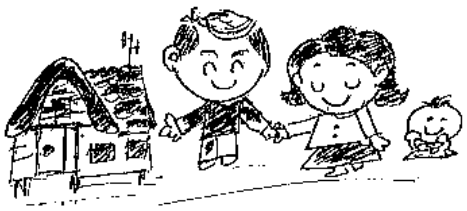

Please keep a record of your member code (TCSID) from the front of the envelope to use as a reference in any future communication with the Thai Health Risk Transition Study

This page will be separated and treated as confidential

Instructions: Please use a blue or black pen to put a cross (**X**) in the ❑ in front of the selected choice to get to this image ☒ **.** Select only **one answer** except when instructed that ***more than one answer can be given.*** For questions that ask for numeric answers, write number(s) **clearly** in the box(es) placing only one number in each box. eg [2][4]

A General Information about you, work, and home

A1 When were you born? *(according to Citizen ID card; please use numbers only)*

[ ][ ] / [ ][ ] / [ ][ ][ ][ ]

Day **Month** **Year** (B.E)

Example [1][5]/[0][1]/[2][5][0][1]

A2 Where is your current residence located?

❑ Countryside ❑ City/Town

A3 How many people live in your home, including yourself

[ ][ ] People *(if you live alone please put* [0][1] *)*

A4 What is your current work status?
(you can choose more than one option)

❑ Doing paid work
❑ Self employed
❑ Help family business but no wage
❑ Doing unpaid work
❑ Look after home/homemaker
❑ Student
❑ Retired (do not work for income)
❑ Seeking work for the first time
❑ Unemployed
❑ Cannot work due to temporary sickness/disability
❑ Cannot work due to permanent sickness/disability
❑ Other

A5 How many hours per week do you work in all paid jobs?

[ ][ ] total hours *(If not working for income put* [0][0]*)*

A6 What is your personal average monthly income *(Baht)*

❑ Up to 3000
❑ 3,001–7,000
❑ 7,001–10,000
❑ 10,001–20,000
❑ 20,001–30,000
❑ 30,001–50,000
❑ 50,001–70,000
❑ 70,001–100,000
❑ Over 100,000

A7 What is your household's average monthly income *(Baht)*

❑ Up to 3000
❑ 3,001–7,000
❑ 7,001–10,000
❑ 10,001–20,000
❑ 20,001–30,000
❑ 30,001–50,000
❑ 50,001–70,000
❑ 70,001–100,000
❑ Over 100,000

A8 What is your weight now? [ ][ ][ ] **kgs**

Example [0][6][2]

A9 What is your height *(no shoes)*? [ ][ ][ ] **cms**

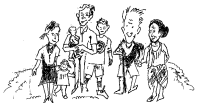

Use enclosed tape measure and instructions on use for the next questions

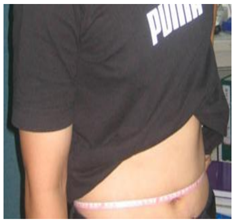

Waist measurement

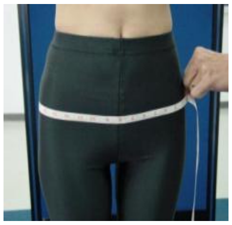

Hip measurement

If you are pregnant please go to question A12

A10 What is your waist measurement? ☐☐☐ **cms**

A11 What is your hip measurement? ☐☐☐ **cms**

A12 What is your current marital status? *(choose only one answer)*

- ☐ First marriage
- ☐ Remarried

Skip to question A14 (First marriage, Remarried)

- ☐ Separated from someone you have been married to (but not divorced)
- ☐ Divorced ☐ Widowed ☐ Never married

A13 If not currently married, do you have a partner? *(choose only one answer)*

☐ Have a partner and live together ☐ Have a partner but do not live together ☐ Do not have a partner

A14 How frequently do you spend time doing each of the following activities? *(cross box that fits best)*

Activities	every day	every week	1-2 times a month	rarely	never
Spend time socially with colleagues from work/your profession					
Spend time socially with friends not connected to work					
Spend time socially with neighbours					
Spend time with parents/other relatives					

A15 Do you regularly care for a sick or disabled family member

☐ No ☐ Yes, part-time ☐ Yes, full-time

A16 In the past 4 weeks, have you had pain in your lower back?
(in the area shown on the diagram)

☐ Yes ☐ No → **Go to B1**

Lower back

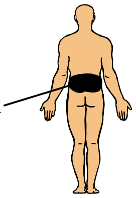

A17 If yes, was this pain bad enough to limit your usual activities or change your daily routine for more than one day?

☐ Yes ☐ No

B Your general health over the past four weeks

B1 Overall how would you rate your health during the past 4 weeks?

- ❑ Excellent
- ❑ Very good
- ❑ Good
- ❑ Fair
- ❑ Poor
- ❑ Very poor

B2 During the past 4 weeks, how much did physical health problems limit your usual physical activities (such as walking or climbing stairs)?

- ❑ Not at all
- ❑ Very little
- ❑ Some
- ❑ Quite a lot
- ❑ Could not do physical activities

B3 During the past 4 weeks, how much difficulty did you have doing your daily work, both at home and away from home, because of your physical health?

- ❑ None at all
- ❑ A little bit
- ❑ Some
- ❑ Quite a lot
- ❑ Could not do daily work

B4 How much bodily pain have you had during the past 4 weeks?

- ❑ None
- ❑ Very mild
- ❑ Mild
- ❑ Moderate
- ❑ Severe
- ❑ Very severe

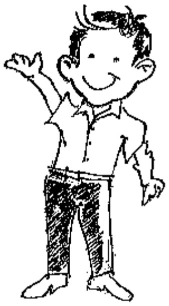

B5 During the past 4 weeks, how much energy did you have?

- ❑ Very much
- ❑ Quite a lot
- ❑ Some
- ❑ A little
- ❑ None

B6 During the past 4 weeks, how much did your physical health or emotional problems limit your usual social activities with family or friends?

- ❑ Not at all
- ❑ Very little
- ❑ Somewhat
- ❑ Quite a lot
- ❑ Could not do social activities

B7 During the past 4 weeks, how much have you been bothered by emotional problems (such as feeling anxious, depressed or irritable)?

- ❑ Not at all
- ❑ Slightly
- ❑ Moderately
- ❑ Quite a lot
- ❑ Extremely

B8 During the past 4 weeks, how much did personal or emotional problems keep you from doing your usual work, school or other daily activities?

- ❑ Not at all
- ❑ Very little
- ❑ Somewhat
- ❑ Quite a lot
- ❑ Could not do daily activities

C Your Life

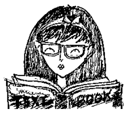

C1 In the past 4 weeks, how much of the time did you feel…
(Please cross box that fits best for each question)

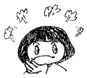

Feeling	all of the time	most of the time	some of the time	a little of the time	none of the time
…so sad nothing could cheer you up?					
…nervous?					
…restless or fidgety?					
…hopeless					
…everything was an effort?					
…worthless?					
…happy?					

C2 Thinking about your own life and personal circumstances, how satisfied are you with…
(Cross box on 0-10 scale that fits best for each question)

Feelings for your life and circumstances	Completely Dissatisfied ◄——► Completely Satisfied										
	0	1	2	3	4	5	6	7	8	9	10
…your standard of living?											
…how safe you feel											
…feeling part of your community											
…your life as a whole											

C3 Have you had to go without things you really needed in the last 12 months because you were short of money?

❑ Yes, often ❑ Yes, sometimes ❑ No, never

C4 Generally speaking, would you say that most people can be trusted or that you can't be too careful in dealing with people?

❑ most people can be trusted ❑ You must be wary of people at all times

C5 Compared to other Thais your age how modern are you?

Modernity	Not modern ◄——► Very modern										
	0	1	2	3	4	5	6	7	8	9	10
Your level of modernity											

C6 What are your views/feelings on the following statements? *(cross the box that corresponds with your feelings)*

Feelings	never	a little	a lot	very much
I am contented with life				
I feel relaxed				
I feel bored and discouraged with my daily life				
I feel disappointed in myself				
I feel life is full of miseries				
I can face and accept problems which are difficult to solve				
I am confident I can control my emotions in case of crisis				
I feel confident in facing life's crises				
I feel sympathetic towards others' suffering				
I feel happy in helping out others when they have problems				
I help others when I have the chance				
I have self esteem				
I feel secure when I stay with my family				
If I become seriously ill I trust my family will take good care of me				
My family members love and care for each other				

D Your food and physical activity

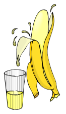
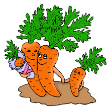

D1 How often, on average, do you eat each of the following food stuffs? *(Cross box that fits best for each question)*

Type of food	Never or < 1/mth	1-3/mth	1-2/wk	3-6/wk	Daily or more
Food/dessert with coconut milk					
Deep fried food					
Instant noodles					
Fermented food eg. Crab, fish					
Soft drink					
Milk – fresh, carton or powder					
Food supplements, vitamins					
Fast food (Western style/farang) eg. hamburger, pizza					
Western bakery products eg. cake, cookies					

D2 How many serves of vegetables do you usually eat each day? ☐☐ **no. of serves per day**

Eg. If you eat 3 serves put in the numbers like this 0 3 *(one serve = ½ cup cooked or 1 cup uncooked)*

D3 How many serves of fruit do you usually eat each day? ☐☐ **no. of serves per day**

Eg. If you eat 5 serves please put the numbers in like this 0 5 *(one serve, equivalent to 1 cup of diced pieces)*

D4 In what way is rice important for you?

	Strongly agree	Agree	Neither agree nor disagree	Disagree	Strongly disagree
I never feel full unless I have eaten rice					
No meal is complete without rice					
Rice is the perfect food					

D5 During a typical week (7-day period) how many times on average do you do each of these physical activities? *(if your answer is 0 times per week mark boxes* 0 0 *if it is 3 times per week mark boxes* 0 3 *)*

Walking continuously, for at least 10 minutes<br>*(for work, recreation or exercise or to get to or from places)*	☐☐ **Times per week**
Vigorous physical activities for more than 20 minutes<br>*(that made you breathe harder or puff and pant)*	☐☐ **Times per week**
Moderate physical activities for more than 20 minutes<br>*(like social tennis, golf, gentle swimming or work around the house or other work)*	☐☐ **Times per week**

D6 How often do you do household cleaning or gardening work?

❑ Seldom or never ❑ 1-3 times/month ❑ Once or twice/week
❑ 3-4 times/week ❑ Most days

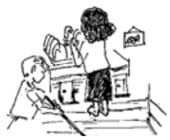

D7 How many hours per day (ie. per 24 hours) do you usually spend on the following activities?

Activities	Duration
**Standing for any purpose at all (eg. for work, while socializing etc.)**	☐☐ **hours/days**
**Sleeping (if you regularly sleep during the day include this also)**	☐☐ **hours/days**
**Watching TV and/or playing computer games?**	☐☐ **hours/days**
**Sitting for any purpose? (eg. reading, resting, writing, thinking, TV, or computer)**	☐☐ **hours/days**

E About the Weather

E1 How often did the hot period this year interfere with the following activities? *(mark the last box if you have air conditioning)*

Activity	Never	1-3 times per month	1-6 times per week	Everyday	Not Applicable- Use Air conditioning
Sleeping					
Housework					
Daily travel					
Work					
Exercise					

F Your injuries

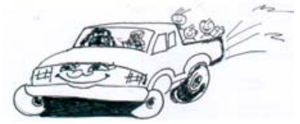

Your injuries – traffic related

F1 In the last 12 months how many times did you get injured in a traffic crash

- ❑ Never → ..go to F7
- ❑ One
- ❑ Three
- ❑ Two
- ❑ Four or more

F2 When you experienced your most serious traffic related injury did you receive medical care?

- ❑ Yes
- ❑ No

F3 Did this injury limit your normal activities for one day or more?

- ❑ Yes
- ❑ No

F4 When this injury occurred what was your role?

- ❑ Driver
- ❑ Passenger
- ❑ Pedestrian → ..go to F6

F5 Type of vehicle you were in as driver or passenger?

- ❑ Bicycle
- ❑ Motorbike
- ❑ Bus, van tour coach
- ❑ Car/pick-up
- ❑ Other (eg. Train, plane, boat)

F6 What was the other party in the collision causing the traffic related injury?

- ❑ Bicycle
- ❑ Motorbike
- ❑ Bus, van, tour coach
- ❑ Car/pick-up
- ❑ Other vehicle (eg. train, boat)
- ❑ Pedestrian
- ❑ Animal (eg. Dog)
- ❑ Other object not vehicle (eg. tree, road surface, wall)

Your injuries – non-traffic related

F7 In the last 12 months how many times did you have a NON-TRAFFIC injury?

- ❑ Never → ..go to G1
- ❑ One
- ❑ Three
- ❑ Two
- ❑ Four or more

F8 When you experienced your most serious non-traffic related injury did you receive medical care?

- ❑ Yes
- ❑ No

F9 Did this injury limit your normal activities for one day or more?

- ❑ Yes
- ❑ No

F10 How were you injured?

- ❑ Assault (punch, push or kick)
- ❑ Other blunt (non-sharp) force
- ❑ Gun shot
- ❑ Stab/cut
- ❑ Fall (not pushed)
- ❑ Fire, heat, scald
- ❑ Near-drowning
- ❑ Poisoning
- ❑ Bite or sting (animal, insect)
- ❑ Choking
- ❑ Other

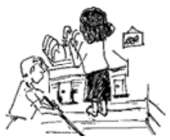

F11 What was the location at which your most serious non-traffic related injury occurred?

- ❑ Home
- ❑ Sports facility
- ❑ Workplace (agricultural)
- ❑ Workplace (non-agricultural)
- ❑ Other

F12 What was the nature of your non-traffic injury?
(you can choose more than one response)

- ❑ Fracture
- ❑ Sprain, strain or dislocation
- ❑ Cut, bite or open wound
- ❑ Bruise or superficial injury
- ❑ Burn/scald
- ❑ Concussion
- ❑ Organ system (internal) injury
- ❑ Other

F13 Was this non-traffic injury?
(cross one box that fits best)

- ❑ Unintentional
- ❑ Intentional by another person
- ❑ Intentional (not involving another person)

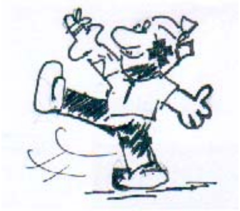

G Your lifetime health history

G1 In your life have you ever experienced a fracture to the areas of your body mentioned below? If so please place a cross in the Yes box and indicate the age at which the fracture occurred.
(for bones broken multiple times put your age for the most recent time)

Don't answer this question if you have never had a fracture ⟶ ..go to G2

Bone Broken	Yes	Age when broken
Example: Wrist (fractured at age 12)	✕	1 2 Years
Finger/Toe		Years
Wrist		Years
Arm		Years
Collarbone		Years
Rib		Years
Skull		Years
Face/Jaw/Nose		Years
Neck		Years
Back		Years
Pelvis		Years
Leg		Years
Ankle		Years
Other		Years

G2 As a result of a diagnosis by a doctor, have you ever been told that you have any of the conditions listed below. If so cross the box and write in your age when the diagnosis was first made *(you can chose more than1answer)*

If you have never been diagnosed with this disease do not place a cross in the box

Disease	Yes	Age when broken	Hospital/Institution/Clinic where you were diagnosed
Example: Measles (diagnosed by Doctor when you were 14)	X	1 4 Years	Krabi Hospital
Diabetes (need to use insulin)		Years	
Diabetes (do not need insulin)		Years	
High cholestrol/high blood lipids		Years	
High blood pressure		Years	
Ischemic (coronary) heart disease		Years	
Cerebrovascular disease (stroke)		Years	
Liver cancer		Years	
Lung cancer		Years	
Stomach cancer		Years	
Colon-rectum cancer		Years	
Breast cancer		Years	
Other cancers		Years	
Goiter/Thyroid disease		Years	
Epilepsy		Years	
Liver disease (not cancer)		Years	
Kidney disease		Years	
Depression/anxiety		Years	
Arthritis		Years	
Chronic bronchitis/lung disease		Years	
Asthma		Years	
Malaria		Years	
Dengue fever		Years	
Tuberculosis		Years	
Other disease (specify)………………………………		Years	

H Tobacco, alcohol and transport

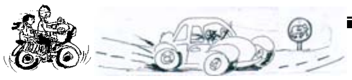

H1 Are you a current smoker

- ❑ Yes..if yes, how many cigarettes per day ☐☐
- ❑ No, I used to smoke but have stopped completely
- ❑ No, I have never smoked

H2 In a typical week how many alcoholic drinks would you have?

☐☐ **glasses per week**

H3 On how many days a week do you usually drink?

☐ **days per week**

(put the number zero 0 *if you don't drink)*

H4 During the last 12 months have you driven a motor vehicle after consuming 3 or more glasses of alcohol

- ❑ Yes
- ❑ No
- ❑ Do not normally drive

H5 Which of the following do you, or any member of your household, own? *(you can choose more than one response)*

- ❑ Bicycle
- ❑ Motorbike
- ❑ Car/pick-up/van
- ❑ Truck
- ❑ Boat
- ❑ No vehicle owned

H6 For personal transport during the last 12 months how often do you…?

	Always	Sometimes	Never	Not applicable	
Use car safety belt Front Seat					No safety belt
Use car safety belt Back Seat					No safety belt
Ride back step of 'song taew'					Don't use 'song taew'
Ride in back of open truck or pick-up					Don't ride that way
Use motorcycle helmet					Don't use motorcycle
Ride on motorcycle with 3 or more people					Don't use motorcycle

R The following questions are to be answered by females only

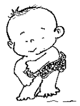

R1 How many babies have you given birth to?

☐☐ *(If none put* 0 0 *)*

R2 Please cross the appropriate box if you have EVER used any of the following hormonal contraceptives

- ❑ Never used any of these
- ❑ Injections every month
- ❑ Injections every 3 months
- ❑ Contraceptive implant eg. Implanon, Norplant
- ❑ Oral contraceptive pill
- ❑ Other

R3 Please cross the appropriate box if you are CURRENTLY using any of the following hormonal contraceptives

- ❑ Not currently using any of these
- ❑ Injections every month
- ❑ Injections every 3 months
- ❑ Contraceptive implant eg. Implanon, Norplant
- ❑ Oral contraceptive pill
- ❑ Other

R4 If you are currently using a contraceptive pill, implant or injections, how long have you used it for?

☐☐ **years** *(if less than one year please put zero* 0 0 *)*

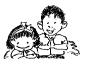

We would like to thank all respondents for their cooperation and assistance
