# Supplementary material for: Methods used for successful follow-up in a large scale national cohort study in Thailand
Source: BMC Res Notes. 2011 May 27;4:166. doi: 10.1186/1756-0500-4-166 (PMC3123220; doi:10.1186/1756-0500-4-166)
Supplement: Additional file 7 — 2009 4-year follow-up questionnaire (Thai). The Thai language 4-year follow-up questionnaire mailed out to all cohort members (n = 85,217) in 2009. [file 1756-0500-4-166-S7.PDF]

แบบสอบถาม

โครงการวิจัยสุขภาพ ปี 2551

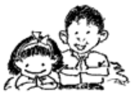

เรียน สมาชิกโครงการวิจัยสุขภาพทุกท่าน

ความก้าวหน้าของโครงการวิจัยสุขภาพที่ท่านมีส่วนสำคัญยิ่งต่อความสำเร็จนั้น ได้ก้าวมาสู่ขั้นการดำเนินงานวิจัยทางสุขภาพอย่างเต็มรูปแบบ ข้อมูลที่ท่านได้ตอบแบบสอบถามกลับมา โครงการฯ ได้นำมาวิเคราะห์ปัจจัยที่ส่งผลต่อสุขภาพและได้จัดทำรายงานในรูป **บันทึกรักษ์สุขภาพ กับโครงการวิจัยสุขภาพ** ส่งให้ทุกท่านแล้ว โดยหวังว่าจะเกิดประโยชน์ต่อท่านและครอบครัวในการดำเนินชีวิตแบบวิถีสุขภาพและมีคุณภาพชีวิตที่ดี

บัดนี้ เป็นช่วงเวลาการติดตามสภาวะสุขภาพของสมาชิกโครงการฯ ข้อมูลที่ท่านตอบกลับมาจะเป็นประโยชน์อย่างยิ่งต่อการสร้างองค์ความรู้ด้านสุขภาพในบริบทของสังคมไทย และ โครงการฯ จะได้ดำเนินการตามแนวจริยธรรมของการวิจัยอย่างเคร่งครัด ข้อมูลรายบุคคลจะถูกเก็บเป็นความลับ สำหรับชื่อและที่อยู่ทางโครงการฯ จะใช้สำหรับการติดต่อกับท่านเท่านั้น โดยตระหนักดีว่าท่านได้ให้ความร่วมมือตอบแบบสอบถามด้วยความสมัครใจ อย่างไรก็ตาม หากท่านไม่มีความประสงค์จะเป็นสมาชิกของโครงการฯ ท่านสามารถแจ้งให้โครงการฯ ทราบทุกเมื่อ

หาก**ท่านคือบุคคลที่ปรากฏชื่อตามเอกสารด้านบน**นี้ และยินดีให้ความร่วมมือกับโครงการวิจัยสุขภาพ มหาวิทยาลัยสุโขทัยธรรมาธิราช โปรดลงชื่อในช่องว่างข้างล่างแล้วกรุณาตอบแบบสอบถาม ส่งกลับมาในซองที่แนบมาพร้อมนี้ โดยไม่ต้องติดแสตมป์

(ลงชื่อ)... วันที่........./........../........

(นาย/นาง/นางสาว ....)

หากมีข้อสงสัย สอบถามรายละเอียดเพิ่มเติมได้ที่หมายเลขโทรศัพท์ 02-5047780 ในเวลาราชการ ขอขอบคุณทุกท่านมา ณ โอกาสนี้

(รองศาสตราจารย์ ดร. สำอาง สืบสมาน)
ผู้อำนวยการศูนย์วิจัยและสร้างเสริมสุขภาวะคนไทย

หน้านี้จะได้รับจัดเก็บเป็นความลับแยกออกจากส่วนอื่น

สิ่งที่สำคัญสูงสุดต่อความสำเร็จของโครงการวิจัยสุขภาพ คือการที่สามารถติดต่อกับสมาชิกทุกท่าน หากท่าน **เปลี่ยนแปลง** ชื่อ-สกุล ที่อยู่ เบอร์โทรศัพท์ ไปจากหน้าปกแบบสอบถาม โปรดตอบในหน้านี้ **โดยใส่เครื่องหมาย × ลงช่องสี่เหลี่ยม โดยใช้ปากกาสีน้ำเงินหรือดำ**

❑ **ไม่มีการเปลี่ยนแปลงข้อมูล** ทั้งชื่อ - สกุล ที่อยู่ และเบอร์โทรศัพท์ → **โปรดข้ามไปอ่านคำชี้แจงหน้าถัดไป**

❑ **มีการเปลี่ยนแปลงข้อมูล** ในรายการต่อไปนี้ ❑ **ชื่อ - สกุล** ❑ **ที่อยู่** ❑ **เบอร์โทรศัพท์**
โดยมีรายละเอียดที่เปลี่ยนแปลงดังนี้

ชื่อ.. นามสกุล ....
ที่อยู่ : เลขที่.. หมู่บ้าน .... ซอย ....
ถนน.. ตำบล/แขวง .... อำเภอ/เขต ....
จังหวัด.. รหัสไปรษณีย์ ☐☐☐☐☐
เบอร์โทรศัพท์บ้าน.. โทรศัพท์ที่ทำงาน..
โทรศัพท์มือถือ.. e-mail..

บุคคลอื่นที่สามารถติดต่อได้ (กรณีที่ติดต่อท่านไม่ได้)

ชื่อ.. นามสกุล ....
ที่อยู่ : เลขที่.. หมู่บ้าน .... ซอย ....
ถนน.. ตำบล/แขวง .... อำเภอ/เขต ....
จังหวัด.. รหัสไปรษณีย์ ....
เบอร์โทรศัพท์บ้าน.. โทรศัพท์ที่ทำงาน..
โทรศัพท์มือถือ..

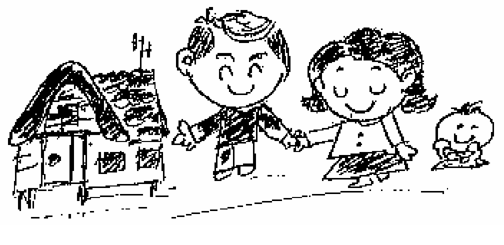

กรุณาจดเลขหมายรหัสสมาชิก TCSID จากหน้าปกเก็บไว้ใช้อ้างอิงในอนาคต เพื่อความสะดวกของท่านในการติดต่อกับโครงการวิจัยสุขภาพ

หน้านี้จะได้รับจัดเก็บเป็นความลับแยกออกจากส่วนอื่น

<u>คำชี้แจง</u> โปรดใช้**ปากกาสีน้ำเงินหรือดำ ใส่เครื่องหมาย × ลงช่องสี่เหลี่ยม** ☐ หน้าตัวเลือกที่ต้องการดังรูป ☒ โดยเลือกเพียงคำตอบเดียว **ยกเว้น**ข้อที่มีระบุว่า "เลือกได้**มากกว่า** 1 คำตอบ" ส่วนคำถามที่ให้เขียนคำตอบเป็นตัวเลขนั้น โปรดใส่ตัวเลขลงภายในช่อง ☐ ช่องละหนึ่งตัวเลข เช่น 24 ให้แยกตัวเลขลงในช่องดังนี้ [2][4]

A ข้อมูลเกี่ยวกับตัวท่านและการงานของท่าน

A1 วัน เดือน ปีเกิดของท่าน (ตามบัตรประจำตัวประชาชน)

[ ][ ] / [ ][ ] / [ ][ ][ ][ ]

วันที่ เดือน ปี พ.ศ.ที่เกิด

(เช่น เกิด 15 มกราคม 2513 โปรดใส่ตัวเลข [1][5]/[0][1]/[2][5][1][3])

A2 บ้านที่ท่านพักอาศัยใน<u>ปัจจุบัน</u>อยู่ที่ไหน

☐ ในชนบท ☐ ในเมือง

A3 บ้านของท่านมีผู้อยู่อาศัยกี่คน (รวมทั้ง*ตัวท่าน*)

จำนวน [ ][ ] **คน** *(ถ้าอยู่คนเดียวโปรดใส่ตัวเลข [0][1])*

A4 งานที่ท่านทำใน<u>ปัจจุบัน</u>มีลักษณะเป็นอย่างไร

(เลือกได้มากกว่า 1 คำตอบ)

- ☐ งานที่มีรายได้
- ☐ อาชีพส่วนตัว
- ☐ ช่วยธุรกิจครอบครัวโดยไม่ได้รับค่าจ้าง
- ☐ งานที่ไม่มีรายได้
- ☐ ดูแลบ้าน/แม่บ้าน/พ่อบ้าน (ให้กับครอบครัว)
- ☐ เป็นนักศึกษา
- ☐ เกษียณแล้ว (และไม่ได้ทำงานที่มีรายได้)
- ☐ กำลังหางานทำเป็นครั้งแรก
- ☐ ว่างงาน
- ☐ ไม่ได้ทำงานเนื่องจากเจ็บป่วย/ทุพพลภาพ**<u>ชั่วคราว</u>**
- ☐ ไม่ได้ทำงานเนื่องจากเจ็บป่วย/ทุพพลภาพ**<u>อย่างถาวร</u>**
- ☐ อื่นๆ

A5 ปัจจุบันท่านใช้เวลาทำงานที่สร้างรายได้ประมาณ สัปดาห์ละกี่ชั่วโมง [ ][ ] **ชั่วโมง**

*(ถ้าเป็นงานที่**<u>ไม่มีรายได้</u>** โปรดใส่ ตัวเลข [0][0])*

A6 ตัวท่านเองมีรายได้ประมาณ<u>เดือนละ</u>เท่าไร (บาท)

- ☐ น้อยกว่าหรือ= 3,000
- ☐ 3,001 - 7,000
- ☐ 7,001 - 10,000
- ☐ 10,001 - 20,000
- ☐ 20,001 - 30,000
- ☐ 30,001 - 50,000
- ☐ 50,001 - 70,000
- ☐ 70,001 - 100,000
- ☐ มากกว่า 100,000

A7 ครอบครัวท่านมีรายได้ประมาณ<u>เดือนละ</u>เท่าไร (บาท)

- ☐ น้อยกว่าหรือ= 3,000
- ☐ 3,001 - 7,000
- ☐ 7,001 - 10,000
- ☐ 10,001 - 20,000
- ☐ 20,001 - 30,000
- ☐ 30,001 - 50,000
- ☐ 50,001 - 70,000
- ☐ 70,001 - 100,000
- ☐ มากกว่า 100,000

A8 ปัจจุบันท่านมีน้ำหนักเท่าไร [ ][ ][ ] **กิโลกรัม**

(เช่น ท่านมีน้ำหนัก 62 กิโลกรัม โปรดใส่ตัวเลข [0][6][2])

A9 ท่านสูงเท่าไร [ ][ ][ ] **เซ็นติเมตร**

(เมื่อไม่สวมรองเท้า)

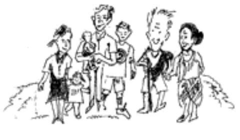

กรุณาใช้สายวัดและคำแนะนำในการวัดที่แนบมาพร้อมนี้ประกอบการตอบคำถามด้านล่าง

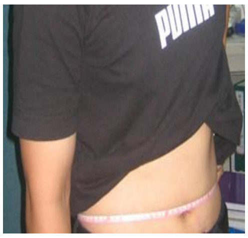

วัดรอบเอว

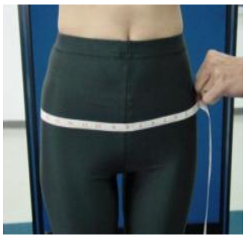

วัดรอบสะโพก

(เช่น ถ้ารอบเอววัดได้ 75 ซ.ม. โปรดใส่ ตัวเลข 0 7 5 *)*

กรณีท่านกำลังตั้งครรภ์ โปรดข้ามไปตอบข้อ A12

A10 รอบเอวของท่านวัดได้ ☐☐☐ **เซ็นติเมตร**

A11 รอบสะโพกของท่านวัดได้ ☐☐☐ **เซ็นติเมตร**

A12 สถานภาพสมรสของท่านในปัจจุบัน *(เลือกเพียง 1 คำตอบ)*

❑ แต่งงานครั้งแรก
❑ แต่งงานครั้งที่ 2 ขึ้นไป } **ข้ามไปตอบข้อ A14**
❑ แยกทางกัน/เลิกกันแล้ว (แต่ยังไม่ได้หย่า)
❑ หย่า ❑ หม้าย (คู่สมรสเสียชีวิต) ❑ ไม่เคยแต่งงาน

A13 หากปัจจุบันยังไม่แต่งงานหรือยังไม่ได้แต่งงานใหม่ ท่านมีคนรัก หรือไม่ *(เลือกเพียง 1 คำตอบ)*

❑ มีคนรัก และอยู่ด้วยกัน ❑ มีคนรัก แต่ไม่ได้อยู่ด้วยกัน ❑ ปัจจุบันยังไม่มีคนรัก

A14 ท่านใช้เวลาทำกิจกรรมเหล่านี้บ่อยแค่ไหน *(โปรดใส่เครื่องหมาย ✕ ลงช่องที่ตรงกับท่านมากที่สุด)*

กิจกรรม	ทุกวัน	ทุกสัปดาห์	1-2 ครั้งต่อเดือน	นานๆ ครั้ง	ไม่เคย
ใช้เวลาสังสรรค์กับเพื่อนร่วมงานหรือร่วมอาชีพ					
ใช้เวลาสังสรรค์กับเพื่อนกลุ่มอื่นๆ ที่ไม่ใช่เพื่อนร่วมงาน					
ใช้เวลาสังสรรค์กับเพื่อนบ้าน					
ใช้เวลากับครอบครัว บิดามารดา หรือเครือญาติ					

A15 ท่านต้องดูแลสมาชิกในครอบครัว หรือคนรู้จักที่ป่วย/ทุพพลภาพ หรือไม่

❑ ไม่ต้อง ❑ ต้องดูแลเป็นบางเวลา ❑ ต้องดูแลตลอดเวลา

A16 ในช่วง 4 สัปดาห์ที่ผ่านมา ท่านปวดหลังบริเวณบั้นเอว หรือไม่ *(ดังในรูป)*

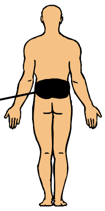

❑ ใช่ ❑ ไม่ใช่ → **ข้ามไปตอบข้อ B1**

A17 อาการปวดหลังนั้นถึงขั้นทำให้ท่านไม่สามารถทำงาน หรือทำกิจกรรมประจำวันอื่นๆ ได้ตามปกติ มากกว่าหนึ่งวันขึ้นไป หรือไม่

❑ ใช่ ❑ ไม่ใช่

B สุขภาพโดยรวมของท่านในช่วง 4 สัปดาห์ที่ผ่านมา

B1 ในช่วง <u>4 สัปดาห์ที่ผ่านมา</u> โดยทั่วไปท่านประเมินสุขภาพของท่านว่าอย่างไร

❑ ดีที่สุด ❑ ดีมาก ❑ ดี
❑ พอใช้ ❑ แย่ ❑ แย่มาก

B2 ในช่วง <u>4 สัปดาห์ที่ผ่านมา</u> ปัญหาสุขภาพกาย จำกัดกิจกรรมทางกายตามปกติของท่าน *(เช่น การเดิน หรือการขึ้นลงบันได)* **มากน้อยแค่ไหน**

❑ ไม่เลย ❑ น้อยมาก ❑ พอสมควร
❑ ค่อนข้างมาก ❑ ไม่สามารถทำกิจกรรมทางกายต่างๆ ได้

B3 ในช่วง <u>4 สัปดาห์ที่ผ่านมา</u> ท่านมีความลำบากในการทำงานประจำวัน ทั้งงานในบ้านและนอกบ้านที่เป็นผลมาจากสุขภาพทางกายของท่าน มากน้อยแค่ไหน

❑ ไม่มีเลย ❑ มีน้อยมาก ❑ มีพอสมควร
❑ มีค่อนข้างมาก ❑ ไม่สามารถทำงานประจำวันได้

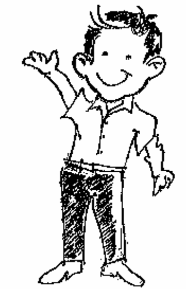

B4 ในช่วง <u>4 สัปดาห์ที่ผ่านมา</u> ท่านมีความเจ็บปวด<u>ทางร่างกาย</u> มากน้อยแค่ไหน

❑ ไม่มีเลย ❑ น้อยมาก ❑ น้อย
❑ ปานกลาง ❑ รุนแรง ❑ รุนแรงมาก

B5 ในช่วง <u>4 สัปดาห์ที่ผ่านมา</u> ท่านมีความรู้สึกกระฉับกระเฉง มากน้อยแค่ไหน

❑ มีมาก ❑ มีค่อนข้างมาก ❑ มีบ้าง
❑ มีเล็กน้อย ❑ ไม่มีเลย

B6 ในช่วง <u>4 สัปดาห์ที่ผ่านมา</u> สุขภาพทางกายหรือ ปัญหาด้านอารมณ์ของท่าน จำกัดกิจกรรมทางสังคมตามปกติของท่านที่มีกับครอบครัว หรือเพื่อน มากน้อยแค่ไหน

❑ ไม่เลย ❑ น้อยมาก ❑ พอสมควร
❑ ค่อนข้างมาก ❑ ไม่สามารถทำกิจกรรมทางสังคมต่างๆได้

B7 ในช่วง <u>4 สัปดาห์ที่ผ่านมา</u> ปัญหาด้านอารมณ์ *(เช่น รู้สึกกังวล ซึมเศร้า หรือหงุดหงิด)* **รบกวนท่าน มากน้อยแค่ไหน**

❑ ไม่เลย ❑ เล็กน้อย ❑ ปานกลาง
❑ ค่อนข้างมาก ❑ มากที่สุด

B8 ในช่วง <u>4 สัปดาห์ที่ผ่านมา</u> ปัญหาส่วนตัวหรือปัญหาด้านอารมณ์ ทำให้ท่านไม่สามารถทำงาน เรียนหนังสือ หรือทำกิจกรรมประจำวันอื่นๆ ของท่านได้ตามปกติ มากน้อยเพียงใด

❑ ไม่เลย ❑ น้อยมาก ❑ พอสมควร
❑ ค่อนข้างมาก ❑ ไม่สามารถทำกิจกรรมประจำวันต่างๆได้

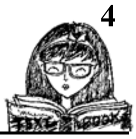

C ชีวิตของท่าน

C1 ในช่วง <u>4 สัปดาห์ที่ผ่านมา</u> ท่านมีความรู้สึกต่อไปนี้บ่อยแค่ไหน

(โปรดใส่เครื่องหมาย ✕ ลงในช่องที่ตรงกับความรู้สึกของท่านในแต่ละข้อ)

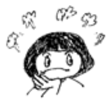

ความรู้สึก	ตลอดเวลา	เกือบตลอดเวลา	บางเวลา	น้อยครั้งมาก	ไม่เคยเลย
รู้สึกเศร้ามาก จนไม่มีอะไรช่วยให้ดีขึ้นได้					
รู้สึกกังวล					
รู้สึกกระวนกระวายหรือหงุดหงิด					
รู้สึกหมดหวัง					
รู้สึกว่าต้องใช้ความพยายามในการทำทุกสิ่งทุกอย่าง					
รู้สึกไร้ค่า					
รู้สึกมีความสุข					

C2 เมื่อคิดถึงชีวิตและสภาพแวดล้อมของท่าน ท่านมีความพอใจกับสิ่งต่อไปนี้ในระดับใด *(โปรดใส่เครื่องหมาย ✕ ลงช่องเพื่อให้ค่า 0-10 คะแนนที่ตรงตามความคิดเห็นของท่านมากที่สุด)*

ความรู้สึกต่อชีวิตและสภาพแวดล้อม	ไม่พอใจอย่างยิ่ง ◄									► พอใจอย่างยิ่ง
	0	1	2	3	4	5	6	7	8	9	10
มาตรฐานการดำรงชีวิต											
ความรู้สึกปลอดภัย											
ความรู้สึกเป็นส่วนหนึ่งของชุมชนที่ท่านอยู่											
ชีวิตโดยรวมของท่าน											

C3 ในช่วง <u>12 เดือนที่ผ่านมา</u> เคยมีหรือไม่ที่ท่านไม่สามารถมีสิ่งที่ต้องการมาก เนื่องจากมีเงินไม่พอ

❑ เคย บ่อยครั้ง ❑ เคยบ้างบางครั้ง ❑ ไม่เคย

C4 โดยทั่วไป ท่านรู้สึกว่าสามารถวางใจผู้อื่นได้เพียงใด

❑ สามารถวางใจได้เกือบทุกคน ❑ ต้องระมัดระวังผู้อื่นตลอดเวลา

C5 เมื่อเทียบกับคนไทยที่มีอายุรุ่นราวคราวเดียวกันแล้ว ท่านคิดว่าตนเองทันสมัยในระดับใด

ความทันสมัย	ไม่เลย ◄									► มากที่สุด
	0	1	2	3	4	5	6	7	8	9	10
ระดับความทันสมัยโดยรวมของท่าน											

C6 ท่านมีความเห็น/รู้สึกอย่างไรในข้อต่อไปนี้ *(โปรดใส่เครื่องหมาย* ✕ *ในช่องที่ตรงกับความรู้สึกของท่าน)*

ความรู้สึก	ไม่เลย	เล็กน้อย	มาก	มากที่สุด
ท่านรู้สึกพึงพอใจในชีวิต				
ท่านรู้สึกสบายใจ				
ท่านรู้สึกเบื่อหน่ายท้อแท้กับการดำเนินชีวิตประจำวัน				
ท่านรู้สึกผิดหวังในตัวเอง				
ท่านรู้สึกว่าชีวิตของท่านมีแต่ความทุกข์				
ท่านสามารถทำใจยอมรับได้ สำหรับปัญหาที่ยากจะแก้ไข (เมื่อมีปัญหา)				
ท่านมั่นใจว่าสามารถควบคุมอารมณ์ได้ เมื่อมีเหตุการณ์คับขันหรือร้ายแรงเกิดขึ้น				
ท่านมั่นใจที่จะเผชิญเหตุการณ์ร้ายแรงที่เกิดขึ้นในชีวิต				
ท่านรู้สึกเห็นอกเห็นใจเมื่อผู้อื่นมีทุกข์				
ท่านรู้สึกเป็นสุขในการช่วยเหลือผู้อื่นที่มีปัญหา				
ท่านให้ความช่วยเหลือแก่ผู้อื่นเมื่อมีโอกาส				
ท่านรู้สึกภูมิใจในตนเอง				
ท่านรู้สึกมั่นคง ปลอดภัยเมื่ออยู่ในครอบครัว				
หากท่านป่วยหนัก ท่านเชื่อว่าครอบครัวจะดูแลท่านเป็นอย่างดี				
สมาชิกในครอบครัวของท่านมีความรักใคร่ปรองดองกัน				

D อาหารและกิจกรรมการเคลื่อนไหวร่างกาย

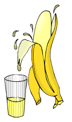

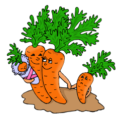

D1 <u>โดยเฉลี่ยแล้ว</u> ท่านรับประทานอาหารต่อไปนี้ บ่อยครั้งแค่ไหน *(โปรดใส่เครื่องหมาย* ✕ *ลงช่องต่อไปนี้)*

ชนิดของอาหาร	ไม่เคย/ น้อยกว่า เดือนละครั้ง	1-3 ครั้ง/ เดือน	1-2 ครั้ง/ สัปดาห์	3- 6 ครั้ง/ สัปดาห์	วันละครั้ง หรือมากกว่า
อาหาร/ขนมหวานที่ประกอบด้วยกะทิ					
อาหารประเภททอด					
บะหมี่กึ่งสำเร็จรูป					
อาหารหมักดอง เช่น ปลาร้า หรือปูดอง (ดิบ)					
เครื่องดื่มประเภทน้ำอัดลม					
นม เช่น นมสด นมกล่อง นมผง ฯลฯ					
ผลิตภัณฑ์เสริมอาหาร เช่น วิตามิน					
อาหารจานด่วนแบบตะวันตก เช่น แฮมเบอร์เกอร์ พิซซ่า					
ขนมแบบตะวันตก เช่น เค้ก คุกกี้ โดนัท					

D2 ท่านรับประทานผักจำนวนกี่ส่วนต่อวัน เช่น ถ้ากิน 3 ส่วน ใส่ตัวเลข [0][3] [ ][ ] **ส่วนต่อวัน**

(ผัก 1 ส่วน = ผักปรุงสุก ครึ่งถ้วยตวง หรือ ผักดิบ 1 ถ้วยตวง)

D3 ท่านรับประทานผลไม้จำนวนกี่ส่วนต่อวัน เช่น ถ้ากิน 5 ส่วน ใส่ตัวเลข [0][5] [ ][ ] **ส่วนต่อวัน**

(ผลไม้ 1 ส่วน = ผลไม้ หั่นเป็นสี่เหลี่ยมลูกเต๋าแล้วตวงได้ 1 ถ้วยตวง หรือกล้วย 1 ลูก = 1 ส่วน หรือ มะละกอ 1 ชิ้น ที่หั่นได้ 5-6 คำ = 1 ส่วน)

D4 ข้าวมีความสำคัญต่อท่านอย่างไร

	เห็นด้วยอย่างยิ่ง	เห็นด้วย	เฉยๆ	ไม่เห็นด้วย	ไม่เห็นด้วยอย่างยิ่ง
กินอะไรก็ไม่รู้สึกอิ่ม ถ้าไม่ได้กินข้าว					
อาหารทุกมื้อจะขาดข้าวไม่ได้					
ข้าวคืออาหารที่สมบูรณ์แบบ					

D5 โดยปกติ ท่านใช้เวลาในการเคลื่อนไหวร่างกายดังต่อไปนี้ ประมาณกี่ครั้งใน<u>หนึ่งสัปดาห์</u>

(ถ้าไม่ได้เคลื่อนไหวร่างกาย ประเภทนั้นๆ โปรดใส่ตัวเลข 0 0 *ถ้าเคลื่อนไหวร่างกาย 3 ครั้งต่อสัปดาห์ โปรดใส่ตัวเลข* 0 3*)*

**เดินอย่างต่อเนื่อง <u>อย่างน้อย 10 นาที</u>**<br>(เช่น เดินเพื่อทำงาน เดินพักผ่อน เดินออกกำลังกาย หรือเดินเพื่อไปถึงที่ใดที่หนึ่ง)	☐☐ **ครั้งต่อสัปดาห์**
**การเคลื่อนไหวร่างกายที่ใช้แรงมาก <u>ตั้งแต่ 20 นาทีขึ้นไป</u>**<br>(ทำให้หายใจแรงและเร็ว เช่น แอโรบิค กีฬาที่ใช้แรงมาก ขี่จักรยาน วิ่ง)	☐☐ **ครั้งต่อสัปดาห์**
**การเคลื่อนไหวร่างกายที่ใช้แรงระดับปานกลาง <u>ตั้งแต่ 20 นาทีขึ้นไป</u>**<br>(เช่น การสังสรรค์ เล่นเทนนิส กอล์ฟ ว่ายน้ำช้าๆ ทำงานบ้าน หรืองานอื่นๆ)	☐☐ **ครั้งต่อสัปดาห์**

D6 ท่านทำงานบ้าน เช่นทำความสะอาด หรือทำสวน บ่อยครั้งเพียงใด

☐ น้อยมากหรือไม่เคยเลย ☐ 1 - 3 ครั้ง / เดือน ☐ 1 - 2 ครั้ง / สัปดาห์

☐ 3 - 4 ครั้ง / สัปดาห์ ☐ เกือบทุกวันหรือทุกวัน

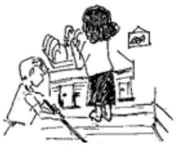

D7 โดยปกติ ในหนึ่งวัน (24 ชั่วโมง) ท่านใช้เวลาในการทำสิ่งเหล่านี้ประมาณวันละกี่ชั่วโมง

กิจกรรม	ระยะเวลา
**ยืนทุกกรณี** (เช่น ยืนทำงาน ยืนคุย ฯลฯ)	☐☐ ชั่วโมงต่อวัน
**นอน** (หากนอนกลางวันเป็นประจำให้นับรวมด้วย)	☐☐ ชั่วโมงต่อวัน
**นั่ง เฉพาะกรณีดูโทรทัศน์ และ/หรือเล่นเกมคอมพิวเตอร์**	☐☐ ชั่วโมงต่อวัน
**นั่งทุกกรณี** (เช่น นั่งอ่านหนังสือ ขีดเขียน นั่งพักผ่อน นั่งคิด รวมเวลาที่นั่งดูโทรทัศน์หรือนั่งทำงาน / เล่นคอมพิวเตอร์)	☐☐ ชั่วโมงต่อวัน

E สภาพภูมิอากาศ

E1 ช่วงอากาศร้อนของปีนี้ ส่งผลกระทบต่อกิจกรรมเหล่านี้ของท่านบ่อยครั้งแค่ไหน

กิจกรรม	ไม่เคย	1-3 ครั้งต่อเดือน	1-6 ครั้งต่อสัปดาห์	ทุกวัน	ไม่เข้าข่าย เพราะใช้เครื่องปรับอากาศ
การนอน					
การทำงานบ้าน					
การเดินทางประจำวัน					
การทำงาน/ประกอบอาชีพ					
การออกกำลังกาย					

F การบาดเจ็บ

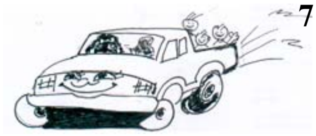

การบาดเจ็บ – ที่เกี่ยวข้องกับการจราจร

F1 ในช่วง 12 เดือนที่ผ่านมา ท่านเคยได้รับบาดเจ็บจากอุบัติเหตุที่เกี่ยวข้องกับการจราจร จำนวนกี่ครั้ง

- ❑ ไม่เคยได้รับบาดเจ็บ → ข้ามไปตอบข้อ F7
- ❑ 1 ครั้ง
- ❑ 3 ครั้ง
- ❑ 2 ครั้ง
- ❑ 4 ครั้งขึ้นไป

F2 การบาดเจ็บที่เกี่ยวกับการจราจรครั้งที่เจ็บหนักที่สุดนั้น ท่านต้องเข้ารับการรักษาพยาบาลหรือไม่

- ❑ ใช่
- ❑ ไม่ใช่

F3 การบาดเจ็บครั้งนั้นมีผลทำให้ไม่สามารถใช้ชีวิตได้ตามปกติตั้งแต่ 1 วันขึ้นไป หรือไม่

- ❑ ใช่
- ❑ ไม่ใช่

F4 การบาดเจ็บครั้งนั้นท่านมีบทบาทอย่างไร

- ❑ เป็นผู้ขับขี่
- ❑ เป็นผู้โดยสาร
- ❑ เป็นผู้สัญจร → ข้ามไปตอบข้อ F6

F5 การบาดเจ็บครั้งนั้น ยานพาหนะที่ท่านขับขี่ หรือโดยสาร คือ

- ❑ รถจักรยาน
- ❑ รถจักรยานยนต์
- ❑ รถโดยสาร/รถตู้/รถทัวร์
- ❑ รถยนต์/รถปิ๊กอัพ
- ❑ อื่นๆ เช่น รถไฟ รถอีแต๋น เรือ

F6 คู่กรณี ที่ทำให้ท่านได้รับการบาดเจ็บครั้งนั้น คือ

- ❑ รถจักรยาน
- ❑ รถจักรยานยนต์
- ❑ รถโดยสาร/รถตู้/รถทัวร์
- ❑ รถยนต์/รถปิ๊กอัพ
- ❑ ยานพาหนะอื่นๆ เช่น รถไฟ รถอีแต๋น เรือ
- ❑ ผู้สัญจร
- ❑ สัตว์ เช่น สุนัข
- ❑ สิ่งอื่นที่ไม่ใช่ยานพาหนะ เช่น ชนต้นไม้ กำแพง ผิวถนน

การบาดเจ็บ – ที่ไม่เกี่ยวข้องกับการจราจร

F7 ในช่วง 12 เดือนที่ผ่านมา ท่านเคยได้รับการบาดเจ็บที่ไม่เกี่ยวข้องกับการจราจร จำนวนกี่ครั้ง

- ❑ ไม่เคยได้รับบาดเจ็บ → ข้ามไปตอบข้อ G1
- ❑ 1 ครั้ง
- ❑ 3 ครั้ง
- ❑ 2 ครั้ง
- ❑ 4 ครั้งขึ้นไป

F8 การบาดเจ็บที่ไม่เกี่ยวกับการจราจรครั้งที่เจ็บหนักที่สุดนั้น ท่านต้องเข้ารับการรักษาพยาบาลหรือไม่

- ❑ ใช่
- ❑ ไม่ใช่

F9 การบาดเจ็บครั้งนั้นมีผลทำให้ไม่สามารถใช้ชีวิตได้ตามปกติตั้งแต่ 1 วันขึ้นไป หรือไม่

- ❑ ใช่
- ❑ ไม่ใช่

F10 การบาดเจ็บครั้งที่เจ็บหนักที่สุดนั้น เกิดขึ้นอย่างไร

- ❑ ถูกทำร้าย (เช่น ถูกชก ผลัก เตะ)
- ❑ ถูกกระแทก/ของตกใส่หรืออื่นๆ
- ❑ ถูกยิง
- ❑ ถูกของมีคมบาด/แทง
- ❑ พลัดตกหรือหกล้มเอง
- ❑ แผลไฟไหม้หรือน้ำร้อนลวก
- ❑ จมน้ำ
- ❑ ถูกสารพิษ
- ❑ ถูกแมลง/สัตว์ กัดหรือต่อย
- ❑ สำลัก
- ❑ อื่นๆ

F11 การบาดเจ็บที่ไม่เกี่ยวกับการจราจรครั้งที่เจ็บหนักที่สุดนั้น เกิดขึ้นที่ไหน

- ❑ ที่พักอาศัย
- ❑ สถานที่เล่นกีฬาหรือออกกำลังกาย
- ❑ สถานที่ทำงาน (ด้านเกษตรกรรม เช่น ไร่ นา)
- ❑ สถานที่ทำงานอื่นๆ (ที่ไม่เกี่ยวกับเกษตรกรรม)
- ❑ อื่นๆ

F12 การบาดเจ็บที่ไม่เกี่ยวกับการจราจรครั้งนั้น มีอาการแบบใด *(เลือกได้มากกว่า 1 คำตอบ)*

- ❑ กระดูกหัก แตก ร้าว
- ❑ เคล็ด ขัดยอกหรือข้อเคลื่อน
- ❑ แผลจากของมีคม แผลถูกกัด หรือแผลเปิด
- ❑ ฟกช้ำหรือบาดเจ็บบริเวณผิวหนัง
- ❑ แผลไฟไหม้ /น้ำร้อนลวก
- ❑ แผลปูด/บวมจากการถูกอัด/กระแทก
- ❑ อวัยวะภายในร่างกายได้รับบาดเจ็บ
- ❑ อื่นๆ

F13 การบาดเจ็บที่ไม่เกี่ยวกับการจราจรครั้งนั้น เกิดขึ้นอย่างไร *(เลือกเพียง 1 คำตอบ)*

- ❑ ไม่ได้ตั้งใจ เป็นอุบัติเหตุ
- ❑ ตั้งใจกระทำโดยบุคคลอื่น
- ❑ ตั้งใจกระทำ (ไม่มีบุคคลอื่นเกี่ยวข้อง)

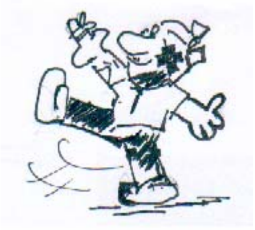

G ประวัติเกี่ยวกับสุขภาพของท่าน

G1 ในชีวิตท่าน เคยมีอาการบาดเจ็บเกี่ยวกับกระดูกหัก แตก ร้าว ในอวัยวะต่อไปนี้หรือไม่ โปรดใส่เครื่องหมาย ✕ ในช่อง **ใช่** และระบุอายุที่เกิดการบาดเจ็บ

ถ้าไม่มีประวัติกระดูกหัก แตก ร้าว ไม่ต้องตอบ ⟶ โปรดข้ามไปตอบข้อ G2

(กรณีกระดูกหักมากกว่า 1 ครั้งที่บริเวณเดิม โปรดใส่อายุในครั้งล่าสุดที่เกิดขึ้น)

อวัยวะที่กระดูกหัก แตก ร้าว	ใช่	อายุ (ที่เกิดกระดูกหัก แตก ร้าว)
ตัวอย่าง เช่น กระดูกข้อมือหักเมื่ออายุ 12 ปี ตอบดังนี้<br>ข้อมือ	✕	1 2 ปี
นิ้วมือ/นิ้วเท้า		☐☐ ปี
ข้อมือ		☐☐ ปี
แขน		☐☐ ปี
ไหปลาร้า		☐☐ ปี
ซี่โครง		☐☐ ปี
ศีรษะ		☐☐ ปี
ทุกส่วนบนใบหน้า/ขากรรไกร/จมูก		☐☐ ปี
คอ		☐☐ ปี
หลัง		☐☐ ปี
กระดูกเชิงกราน		☐☐ ปี
ขา		☐☐ ปี
ข้อเท้า		☐☐ ปี
อื่น ๆ		☐☐ ปี

G2 ท่านเคยได้รับแจ้ง <u>ผลการตรวจวินิจฉัยโดยแพทย์</u> ว่าท่านเป็นโรคต่อไปนี้หรือไม่ (เลือกตอบได้มากกว่า 1 คำตอบ)

โดยใส่เครื่องหมาย ✕ ในช่อง เป็น **และโปรดระบุอายุของท่านเมื่อได้รับทราบ<u>ผลการตรวจวินิจฉัยครั้งแรก</u> ว่าเป็นโรคนั้น**

ถ้าไม่มีประวัติเป็นโรคที่วินิจฉัยโดยแพทย์ ***ไม่ต้อง*** *ใส่เครื่องหมาย ✕ ลงในช่อง*

**รายการโรคที่ตรวจพบโดยแพทย์**	**เป็น**	**อายุที่ตรวจพบครั้งแรก**	**รพ./สถาบัน/คลินิค ฯลฯ ที่ให้การวินิจฉัย**
ตัวอย่าง โรคหัด (วินิจฉัยโดยแพทย์เมื่ออายุ 14 ปี)	✕	1 4 ปี	โรงพยาบาลกระบี่
เบาหวาน (ต้องใช้อินซูลิน)		☐☐ ปี	
เบาหวาน (ไม่ต้องใช้อินซูลิน)		☐☐ ปี	
โคเลสเตอรอลสูงหรือไขมันในเลือดสูง		☐☐ ปี	
ความดันโลหิตสูง		☐☐ ปี	
โรคหัวใจขาดเลือด		☐☐ ปี	
โรคหลอดเลือดในสมอง (Stroke)		☐☐ ปี	
มะเร็งตับ		☐☐ ปี	
มะเร็งปอด		☐☐ ปี	
มะเร็งกระเพาะอาหาร		☐☐ ปี	
มะเร็งลำไส้		☐☐ ปี	
มะเร็งเต้านม		☐☐ ปี	
มะเร็งอวัยวะอื่นๆ		☐☐ ปี	
คอพอก/ต่อมไทรอยด์ผิดปกติ		☐☐ ปี	
ลมบ้าหมู		☐☐ ปี	
โรคเกี่ยวกับตับ (ไม่ใช่มะเร็ง)		☐☐ ปี	
โรคเกี่ยวกับไต		☐☐ ปี	
ซึมเศร้า/วิตกกังวล		☐☐ ปี	
ข้ออักเสบ		☐☐ ปี	
หลอดลมอักเสบเรื้อรัง/โรคปอดอื่นๆ		☐☐ ปี	
หอบหืด		☐☐ ปี	
มาลาเรีย (ไข้จับสั่น)		☐☐ ปี	
ไข้เลือดออก		☐☐ ปี	
วัณโรค		☐☐ ปี	
โรคอื่นๆ(ระบุ)..		☐☐ ปี	

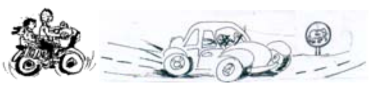

H บุหรี่ แอลกอฮอล์ และการเดินทาง

H1 ปัจจุบันท่านสูบบุหรี่หรือไม่

- ☐ สูบ โดยสูบวันละ ☐☐ มวน

(เช่น ถ้าสูบวันละ 3 มวน โปรดใส่ตัวเลข 0 3 *)*

- ☐ เคยสูบแต่ปัจจุบันเลิกสูบโดยเด็ดขาด
- ☐ ไม่เคยสูบ

H2 โดยทั่วไป ท่านดื่มเครื่องดื่มแอลกอฮอล์สัปดาห์ละกี่แก้ว ☐☐ แก้วต่อสัปดาห์

(ถ้าไม่ดื่มแอลกอฮอล์ โปรดใส่ตัวเลข 0 0 *)*

H3 โดยทั่วไป <u>ใน 1 สัปดาห์</u> ท่านดื่มเครื่องดื่มแอลกอฮอล์สัปดาห์ละกี่วัน ☐ วันต่อสัปดาห์

(ถ้าไม่ดื่มแอลกอฮอล์ โปรดใส่ตัวเลข 0 *)*

H4 ในช่วง <u>12 เดือนที่ผ่านมา</u> ท่านเคยขับขี่ยานพาหนะ <u>หลังดื่มแอลกอฮอล์ตั้งแต่ 3 แก้วขึ้นไป</u> หรือไม่

- ☐ เคย
- ☐ ไม่เคย
- ☐ ปกติไม่ได้ขับขี่ยานพาหนะ

H5 ท่านหรือบุคคลในบ้านท่านเป็นเจ้าของยานพาหนะใดบ้าง *(เลือกได้มากกว่า 1 คำตอบ)*

- ☐ จักรยาน
- ☐ จักรยานยนต์
- ☐ รถยนต์/ปิ๊กอัพ/รถตู้
- ☐ รถบรรทุก
- ☐ เรือ
- ☐ ไม่มียานพาหนะ

H6 ในช่วง 12 เดือนที่ผ่านมา ท่านกระทำสิ่งต่อไปนี้บ่อยครั้งเพียงใด ในการเดินทาง

การเดินทาง	ทำประจำ	ทำบางครั้ง	ไม่เคยทำ	ไม่เข้าข่าย เนื่องจาก	
คาดเข็มขัดนิรภัยเมื่อนั่งรถยนต์ตอนหน้า					รถไม่มีเข็มขัดนิรภัย
คาดเข็มขัดนิรภัยเมื่อนั่งรถยนต์ตอนหลัง					รถไม่มีเข็มขัดนิรภัย
นั่ง/ยืนบริเวณบันไดท้ายรถสองแถว					ไม่ใช้รถสองแถว
นั่งในกระบะท้ายไม่มีหลังคา					ไม่นั่งในกระบะท้าย
สวมหมวกนิรภัยขณะขับขี่/ซ้อนมอเตอร์ไซค์					ไม่ใช้รถมอเตอร์ไซค์
ขับขี่/ซ้อนมอเตอร์ไซค์สามคนหรือมากกว่า					ไม่ใช้รถมอเตอร์ไซค์

R คำถามต่อไปนี้เฉพาะสตรีเป็นผู้ตอบ

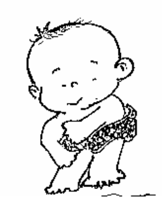

R1 ท่านเคยให้กำเนิดบุตรมาแล้วทั้งหมดกี่คน

☐☐ **คน**

(ถ้าไม่เคยให้กำเนิดบุตร โปรดใส่ตัวเลข 0 0 *)*

R2 ท่าน<u>เคย</u>ใช้ยาคุมกำเนิดประเภทใดบ้าง ดังต่อไปนี้

(ตอบได้มากกว่า 1 คำตอบ)

- ☐ <u>ไม่</u>เคยใช้ยาคุมกำเนิด
- ☐ ยาฉีดคุมกำเนิด ชนิดฉีด<u>ทุก 1 เดือน</u>
- ☐ ยาฉีดคุมกำเนิด ชนิดฉีด<u>ทุก 3 เดือน</u>
- ☐ ยาฝังคุมกำเนิด
- ☐ ยาเม็ดคุมกำเนิด ☐ อื่นๆ

R3 <u>ปัจจุบัน</u> ท่านใช้ยาคุมกำเนิดประเภทใด

- ☐ <u>ไม่</u>ใช้ยาคุมกำเนิด
- ☐ ยาฉีดคุมกำเนิด ชนิดฉีด<u>ทุก 1 เดือน</u>
- ☐ ยาฉีดคุมกำเนิด ชนิดฉีด<u>ทุก 3 เดือน</u>
- ☐ ยาฝังคุมกำเนิด
- ☐ ยาเม็ดคุมกำเนิด ☐ อื่นๆ

R4 หาก <u>ปัจจุบัน</u> ท่านใช้ยาคุมกำเนิดไม่ว่าชนิดเม็ด ฝัง หรือฉีด ท่านใช้มานานเท่าไรแล้ว ☐☐ ปี

(ถ้าใช้มาน้อยกว่า 1 ปี โปรดใส่ตัวเลข 0 0 *)*

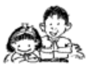

ขอขอบคุณทุกท่านสำหรับความร่วมมืออย่างดียิ่ง
